# Supplementary material for: Reproducibility of individual effect sizes in meta-analyses in psychology
Source: PLoS One. 2020 May 27;15(5):e0233107. doi: 10.1371/journal.pone.0233107 (PMC7252608; doi:10.1371/journal.pone.0233107)
Supplement: S1 File — https://osf.io/pf4x9/. (PDF) [file pone.0233107.s001.pdf]

Reproducibility of Individual Effect Sizes in Meta-Analyses in Psychology
Supplement A. Additional Results

Supplement A contains additional preregistered results that were not included in the main article. Our preregistration can be found at https://osf.io/v2m9j. Our main repository can be found at https://osf.io/7nsmd/. At the end of this document, a table is displayed with all meta-analyses, the number of primary study effect sizes each meta-analysis contained, the number of primary study effect sizes we sampled, and the reporting results displayed in this supplement summarized.

Additional Results Part 2

In Part 2 we investigated meta-analysis reproducibility based on the subset of primary study effect sizes that were sampled ($m = 33$ with $k = 500$ primary study effect sizes), instead of complete meta-analyses including all studies. Next, we will present results as in Part 2 of the paper, but now after analyzing the complete meta-analyses ($m = 33$ with $k = 1{,}978$ primary study effect sizes).

For each of the 33 meta-analyses, we conducted two analyses. The first analysis was performed on the complete meta-analysis as reported in the paper. The second analysis was also performed on the complete meta-analysis as reported in the paper, but after replacing the sampled primary study effect sizes with our recalculated primary study effect sizes. We then documented the pooled meta-analytic effect size estimate, its confidence interval, and the τ^2 parameter for both the reported and reproduced meta-analysis, and compared these outcomes for discrepancies.[1]

For meta-analytic outcomes, 7 out of 33 meta-analyses (21%) showed discrepancies in either the pooled effect size estimate, its confidence interval, or τ^2 parameter. This estimate is smaller than in the subset meta-analyses, which is expected, because in the subsets we only analyzed reproduced primary study effect sizes. In the complete meta-analyses, the proportion of number of primary study effect sizes that were not sampled (and thus do not cause any discrepancies in outcomes) is higher than in the subset meta-analyses.

We found small discrepancies in the pooled effect size estimates for three out of 33 meta-analyses (11%). We found seven meta-analyses with discrepancies in the confidence intervals (21%), of which six were small and one was moderate. In line with our hypothesis, this result shows that corrected primary study effect sizes have a larger impact on the

[1] Which discrepancies were classified as small, moderate or large depends on the type of effect size. We transformed our discrepancy measures for correlations r (small $\geq .025$ and $< .075$], moderate [$\geq .075$ and $<$ .125], and large [$\geq .125$] to other types of effect sizes based on $N = 64$, relating to the 50th percentile of the degrees of freedom of reported test statistics in eight major psychology journals (Hartgerink, Wicherts, & van Assen, 2017). For Hedges' g, classifications were small [$\geq .049$ and $< .151$], moderate [$\geq .151$ and $< .251$], and large [$\geq .250$]. For Cohen's d, classifications were small [$\geq .050$ and $< .152$], moderate [$\geq .152$ and $<$ .254] and large [$\geq .254$]. For correlations z, classifications were the same as r.

boundaries of the confidence interval than the pooled effect size estimate. The discrepancies between the τ^2 parameter estimates only exceeded 0.1 in one of the 33 meta-analyses.

In none of the meta-analyses was the statistical significance of the average effect size affected by using the recalculated primary study effect sizes. The heterogeneity estimate changed from statistically significant to non-significant in one meta-analysis, and from statistically non-significant to significant in two meta-analyses.

Additional results from meta-analyses excluding "incomplete" effect sizes

A reviewer to a previous version of this paper rightfully claimed that it is problematic to include the *incomplete* effect sizes in the tests of meta-analytic reproducibility, since these effects could not be reproduced due to a lack of information. Including these incomplete effect sizes might deflate the differences between the original and reproduced meta-analyses. As such, we decided to compare the reported and reproduced meta-analyses again, only including effect sizes we could calculate: *correct*, *incorrect*, and *ambiguous* effect sizes. Note that this procedure is the same as the one printed in the main manuscript, with the only difference being that the incomplete effect sizes are removed from all meta-analyses.

For meta-analytic outcomes, 14 out of 33 meta-analyses (42%) showed discrepancies in either the pooled effect size estimate, its confidence interval, or τ^2 parameter. This result is comparable to the subset meta-analyses in the manuscript (13 meta-analyses, 39%), but the discrepancies were notably larger when we excluded the incomplete effect sizes.

We found small discrepancies in the pooled effect size estimates for 10 out of 33 meta-analyses (30%, one more than the analyses in the manuscript), and one meta-analysis had a moderate discrepancy in the pooled effect size estimate (3%, one more than the analyses in the manuscript).

We found 14 meta-analyses with discrepancies in the confidence intervals (42%), of which nine were small, three were moderate, and two were large (analyses in manuscript: nine small, three moderate, zero large). In total, 12 of the τ^2 parameter estimates increased (same result as in the manuscript), 16 decreased (one less than in the manuscript), and five remained the same (one more than in the manuscript).

In none of the meta-analyses was the statistical significance of the average effect size affected by using the recalculated primary study effect sizes. The heterogeneity estimate changed from statistically significant to non-significant in two meta-analyses (one more than in the manuscript), and from statistically non-significant to significant in one meta-analysis (the same result as in the manuscript).

Reporting standards: heterogeneity

For each meta-analysis we reported which models the authors used for their meta-analysis, and whether they performed any additional analyses (e.g., moderator or subgroup analyses, meta-regression) to account for heterogeneity in their sample.

We found 22 out of 33 meta-analyses used random-effects models, whereas one used a fixed effect model, three used both fixed effect and random-effects models, and in seven meta-

analyses it was not reported which models were used. Regarding software, ten meta-analyses reported using Comprehensive Meta-Analysis, two used SPSS, one used the Schmidt-Le program, one used Stata, and 19 meta-analyses did not report which software they used. For estimators, six meta-analyses reported the use of the Hunter and Schmidt (HS) approach, six reported the use of the Lipsey and Wilson (LW) approach, two the Hedges and Olkin (HO) estimator, two the DerSimonian and Laird (DL) estimator, two a method-of-moments (MoM) approach, one the Restricted Maximum Likelihood (REML) estimator, one a Maximum Likelihood (ML) estimator, and 13 meta-analyses did not report which estimator they used. Overall, the information provided by the authors is not enough to ensure reproducibility of the results. At the very least it should be reported whether a fixed effect, random-effects, or mixed-effects model is used, and what software is used to estimate the results.

In total, 30 meta-analyses inspected the influence of moderators or performed subgroup analysis or meta-regression. It should be noted that in one additional meta-analyses (i.e., number 23) the authors mentioned moderator analysis, but refrained from performing it because of small sample sizes and missing values. Overall the reporting on heterogeneity is adequate, and most authors follow up with further analyses to explain possible heterogeneity.

Reporting standards: outliers

We documented for each meta-analysis whether the authors mentioned outliers, and if so, whether any subsequent or separate analyses were performed to investigate the impact of said outliers. In total, 10 out of 33 meta-analyses mention outliers, and 7 of these 10 perform separate analyses with and without outliers. This result confirms our expectation that the overall impact of outliers is undervalued and underreported on in this sample of meta-analyses.

Reporting standards: publication bias

For each meta-analysis we documented whether publication bias was mentioned by the meta-analysts, and if so, which methods were used to investigate it. In total, 22 meta-analyses reported on publication bias. Additionally, 17 meta-analyses reported finding no evidence for publication bias in their sample, 4 meta-analyses reported finding publication bias, and 12 meta-analyses did not report any findings on publication bias. The most frequently used methods were the fail-safe N and adjusted fail-safe N method (used 12 times), funnel plot inspection without specification of any test (11 times), the trim and fill method (9 times), Egger's test (6 times), and publication status was used in meta-regression a total of 5 times. The mean number of methods used per meta-analysis was 1.

Dependency of primary study effect sizes

It is often the case that in a meta-analysis several different outcomes from one study are combined, leading to multiple effect sizes from the same study being included in one meta-analysis. If these multiple effect sizes are drawn from the same sample of participants, these effect sizes will be dependent because the same sample is included for more than one outcome, and thus counted more than once (Moeyaert et al., 2016). Research has shown dependent effect sizes are common in meta-analyses, but reporting on and investigation of

the phenomenon are low (Ahn, Ames, & Myers, 2012; Cheung & Chan, 2004). We inspected whether dependency among studies is present by investigating pairs of primary studies with the same author and homogeneous effect sizes on whether their samples are independent or not.

To check for dependency in effect sizes, we first sorted the complete original meta-analyses by effect size, and a fixed effect model was fitted for each subsequent pair of studies (e.g., study 1 with 2, study 2 with 3, study 3 with 4 and so on). If the p-value of the Q statistic of a pair of studies is higher than .80, the effect sizes are relatively homogeneous. All pairs of studies with a Q statistic p-value of .80 or higher were retained. Of these pairs of homogeneous studies, we chose the studies where at least one author was the author of both studies. These pairs of studies were retrieved and we checked whether the same samples were used in both studies, by investigating where the study took place and what type of participants were included. Since most papers omit specific details of the respondents or the institution where the experiment took place, we can only give an indication of dependency, and cannot be sure whether the data is actually dependent. Over all meta-analyses combined, we found a total of 248 possible pairs of dependent primary effect sizes (i.e., homogeneous effect sizes with a p-value of more than .80 for the Q test, with at least one author being the same in both studies). Of those 248 pairs, we were unable to locate 47, leaving 201 pairs to be investigated. If a pair of primary studies *within* one paper was indicated as possibly dependent (e.g., experiment 1 with experiment 2), we assumed different samples were used and no dependency was reported. Of the 201 pairs, 19 had an indication of having used the same sample of students (e.g., psychology students) from the same institutions (e.g., a specific university). In total, 11 out of 33 meta-analyses contained pairs of primary studies with possible dependent effect sizes. We refer to the document "codebook dependent studies" for more detailed information on which pairs of studies were checked and corresponding results.

We also documented for all 33 meta-analyses whether the authors dealt with the issue of dependent effect sizes. In total, 31 out of 33 meta-analyses mentioned using independent samples or the possibility of dependent effect sizes. Of those 31 meta-analyses, 7 did not report anything related to methods to ensure effect size independence. Various methods were used by the other 24 meta-analyses: most used an average when multiple relevant correlations were reported from the same sample ($m = 10$), some chose only one publication out of multiple studies ($m = 3$), some adjusted the sample size for the groups that were included multiple times in one meta-analysis ($m = 3$), and some used aggregation methods or other methodological approaches ($m = 6$). One meta-analysis chose the effect size belonging to the largest sample, and one meta-analysis reported the use of multiple methods to account for dependency in effect sizes. Since so many different methods are used to account for effect size dependency, we recommend standards be created to account for primary study effect size dependency.

Effect of outliers on pooled effect size estimates

We investigated the impact of outlying primary studies on meta-analytic pooled effect size estimates for the subset meta-analyses. For each meta-analysis, we fitted a random-effects model and calculated the pooled effect size. Afterwards, we repeatedly fitted the model

again via the leave-one-out method (Viechtbauer, 2010), leaving out one study at a time, and we calculated new estimates. For each meta-analysis we checked how many primary studies (which we defined as outliers) have small, moderate, or large effects on the pooled effect size estimate. The discrepancy measures match those from the primary studies and meta-analyses.[2] We expected primary studies to have mostly small effects on meta-analytic outcomes.

For pooled effect size estimates, our results confirm this expectation: 75 primary studies had a small effect on the pooled effect size estimate, 3 had a moderate effect, and 1 had a large effect. We note that for two meta-analyses (i.e., number 9 and 15) it was not possible to calculate the effect of outliers on pooled effect size estimates because of different estimation methods that did not allow for leave-one-out testing (i.e., we calculated the results for meta-analysis number 9 without using the metafor package (Viechtbauer, 2010), and meta-analysis number 15 used a multivariate/multilevel linear (mixed-effects) model).

Non-retrieval of primary studies

In total, we were unable to retrieve 154 primary papers from 26 out of 33 (79%) meta-analyses. Of these 154 primary papers, 70 (45%) were journal articles, 48 (31%) were dissertations, 8 (5%) were papers presented at conferences, 7 (5%) were unpublished manuscripts, 7 (5%) were posters, 3 (2%) were unpublished master theses, 3 (2%) were unpublished raw data, and 1 (1%) was a report. For more detailed results we refer to the document “nonretrieved primary studies”.

Replication of the reported pooled meta-analytic effect size estimate

In several meta-analyses (m = 9 out of 33) we encountered problems with replicating the meta-analytic effect size estimate, based on the effect size estimates in the data table and information reported in the paper. Our results were however always similar to the results presented in the paper, with the exception of one meta-analysis (number 27), which reported an effect size of $r = 0.66$, whereas we calculated an effect size of $r = 0.38$ based on the reported data. The second largest effect size estimate difference was d = -0.22 (i.e., meta-analysis number 16 reported an effect size of $d = 2.02$, whereas we calculated an effect size of $d = 2.24$).

[2]Which discrepancies were classified as small, moderate or large depends on the type of effect size. We transformed our discrepancy measures for correlations r (small $\geq$ .025 and $<$ .075], moderate [$\geq$ .075 and $<$ .125], and large [$\geq$ .125] to other types of effect sizes based on $N = 64$, relating to the 50th percentile of the degrees of freedom of reported test statistics in eight major psychology journals (Hartgerink et al., 2017). For Hedges' g, classifications were small [$\geq$ .049 and $<$ .151], moderate [$\geq$ .151 and $<$ .251], and large [$\geq$ .250]. For Cohen's d, classifications were small [$\geq$ .050 and $<$ .152], moderate [$\geq$ .152 and $<$ .254] and large [$\geq$ .254]. For correlations z, classifications were the same as r.

Table 1. *Descriptives per meta-analysis on number of primary study effect sizes and reporting standards.*

MA	k tot	k samp	k disc	Model	Est	Subgroup	Outlier	Pub bias	Dep	Rep
1	57	20	8	NA	NA	y	y	y	n	n
2	108	20	15	FE+RE	NA	y	n	y	n	n
3	13	11	1	RE	MoM	n	n	y	n	n
4	270	21	7	FE+RE	HO	y	n	y	y	n
5	21	11	10	RE	HS	y	n	n	y	n
6	21	11	10	NA	HS	y	n	n	n	n
7	283	23	2	NA	HS	y	n	n	n	n
8	11	9	4	RE	NA	y	y	n	y	n
9	67	22	5	NA	HS	y	n	n	y	n
10	80	21	4	NA	HS	y	n	y	y	n
11	93	20	15	RE	LW	y	n	y	y	n
12	18	11	10	RE	NA	y	n	n	y	n
13	105	20	5	FE+RE	NA	y	n	y	n	n
14	94	20	7	RE	ML	y	n	y	y	n
15	154	20	0	RE	REML	y	y	y	n	n
16	16	13	8	RE	NA	y	n	y	n	n
17	43	11	8	RE	NA	y	y	y	y	n
18	20	14	7	RE	HO	y	n	n	y	n
19	48	20	12	RE	LW	y	y	y	y	n
20	16	10	9	RE	LW	y	n	n	y	n
21	16	10	3	RE	NA	y	n	y	y	n
22	8	8	0	RE	NA	y	n	y	y	n
23	15	9	8	RE	MoM	n	y	y	y	n
24	29	16	11	RE	DL	y	n	y	y	n
25	9	8	4	RE	DL	y	n	y	y	y
26	184	21	7	RE	LW	y	n	n	y	n
27	30	19	5	RE	LW	y	n	y	y	n
28	37	20	14	RE	NA	y	n	y	y	n
29	17	10	1	NA	HS	y	n	n	n	n
30	31	20	13	RE	NA	y	n	y	y	n
31	40	13	7	FE	LW	y	n	n	y	n
32	12	6	2	NA	NA	n	n	y	n	n
33	12	11	0	RE	NA	y	y	y	y	n

Note: *MA*: meta-analysis number; *k total*: the total number of primary study effect sizes included in the meta-analysis; *k samp*: the number of primary study effect sizes we sampled; *k disc*: the number of primary study effect sizes that contained a discrepancy; *Model*: the model used for the meta-analysis (FE = fixed effect, RE = random-effects); *Est*: the estimator used for the meta-analysis (HS = Hunter and Schmidt, LW = Lipsey and Wilson, HO = Hedges and Olkin, DL = DerSimonian and Laird, MoM = Method-Of-Moments, REML = Restricted

Maximum Likelihood, ML = Maximum Likelihood; *Subgroup*: whether subgroup analysis was performed; *Outliers*: whether separate analyses were performed with and without outliers; *Pub bias*: whether one or more publication bias methods were used; *Dep*: whether the authors dealt with the issue of dependent effect sizes; *Rep*: whether the authors mentioned adhering to reporting guidelines.

References

Ahn, S., Ames, A. J., & Myers, N. D. (2012). A Review of Meta-Analyses in Education. *Review of Educational Research*, *82*(4), 436–476. doi:10.3102/0034654312458162

Cheung, S. F., & Chan, D. K.-S. (2004). Dependent Effect Sizes in Meta-Analysis: Incorporating the Degree of Interdependence. *Journal of Applied Psychology*, *89*(5), 780–791. doi:10.1037/0021-9010.89.5.780

Hartgerink, C. H. J., Wicherts, J. M., & van Assen, M. A. L. M. (2017). Too Good to be False: Nonsignificant Results Revisited. *Collabra: Psychology*, *3*(1), 9. doi:10.1525/collabra.71

Moeyaert, M., Ugille, M., Beretvas, S. N., Ferron, J., Bunuan, R., & van den Noortgate, W. (2016). Methods for dealing with multiple outcomes in meta-analysis: A comparison between averaging effect sizes, robust variance estimation and multilevel meta-analysis. *International Journal of Social Research Methodology*, *20*(6), 559–572. doi:10.1080/13645579.2016.1252189

Viechtbauer, W. (2010). Conducting meta-analyses in R with the metafor package. *Journal of Statistical Software*, *36*(3), 1–48.
