# Supplementary material for: Reproducibility of individual effect sizes in meta-analyses in psychology
Source: PLoS One. 2020 May 27;15(5):e0233107. doi: 10.1371/journal.pone.0233107 (PMC7252608; doi:10.1371/journal.pone.0233107)
Supplement: S2 File — https://osf.io/43ju5/. (PDF) [file pone.0233107.s002.pdf]

# Reproducibility of Individual Effect Sizes in Meta-Analyses in Psychology
# Supplement B
# Sampling scheme and flowcharts

Supplement B contains the sampling scheme for the 33 meta-analyses (referenced at the end of this document), as well as the sampling scheme for the 500 primary study effect sizes within the meta-analyses. For both, flowcharts are included.

## Meta-analysis selection

Our sample of meta-analyses (total $m = 33$) was chosen from previous research that had coded materials readily available (Bakker, van Dijk, and Wicherts, 2012; Wijsen, 2015). Suitable meta-analyses from the first study (Wijsen, 2015) were published in 2011 or 2012 and available in the database PsycArticles with "*meta-a**" as a search criterion. The goal of the meta-analysis selection was to obtain a representative sample of psychological studies, but the inclusion criteria may have affected the representativeness of the sample. More specifically, a large number ($m = 97$) of meta-analyses could not be included because they omitted necessary basic statistics. For instance, they did not report a data table with primary studies, effect sizes and standard errors or within-study variances, the meta-analyses did not contain the appropriate effect sizes (i.e., SMDs or correlations), or did not meet the minimum requirement of ten primary studies, to ensure power of the meta-analysis was large enough to provide accurate estimation of effect sizes. The number of eligible meta-analyses remaining was 52, of which 22 were randomly sampled by Wijsen, 2015 because of feasibility constraints [1].

The final 11 meta-analyses (Bakker et al., 2012) were selected from the total number of eligible peer-reviewed articles published in 2011 from PsycArticles ($m$= 129), containing the strings "*research synthesis*", "*systematic review*", or "*meta-anal**" in the title and/or abstract. A total of 21 meta-analyses were excluded because the meta-analyses did not contain ($m$=2) or did not report ($m$=13) effect sizes of primary studies, there were fewer than 10 primary studies ($m$=3), unavailability ($m$=1), or samples were dependent, leading to biased standard errors ($m$=2). Of the remaining 108 meta-analyses, Bakker et al. randomly selected 11 (10% of the total). In 16 cases, there were multiple meta-analyses within one article, in which case we randomly selected one meta-analysis. The total number of selected meta-analytic articles (and meta-analyses) was 33, which contained 1978 primary effect sizes, of which we sampled 500 to reproduce.

[1] We note that Wijsen actually randomly sampled 23 instead of 22 meta-analyses. However, both Wijsen and Bakker et al. sampled the meta-analysis by Card et al., 2011. For clarity, we included the Card et al. article in the sample of 11 meta-analyses by Bakker et al.

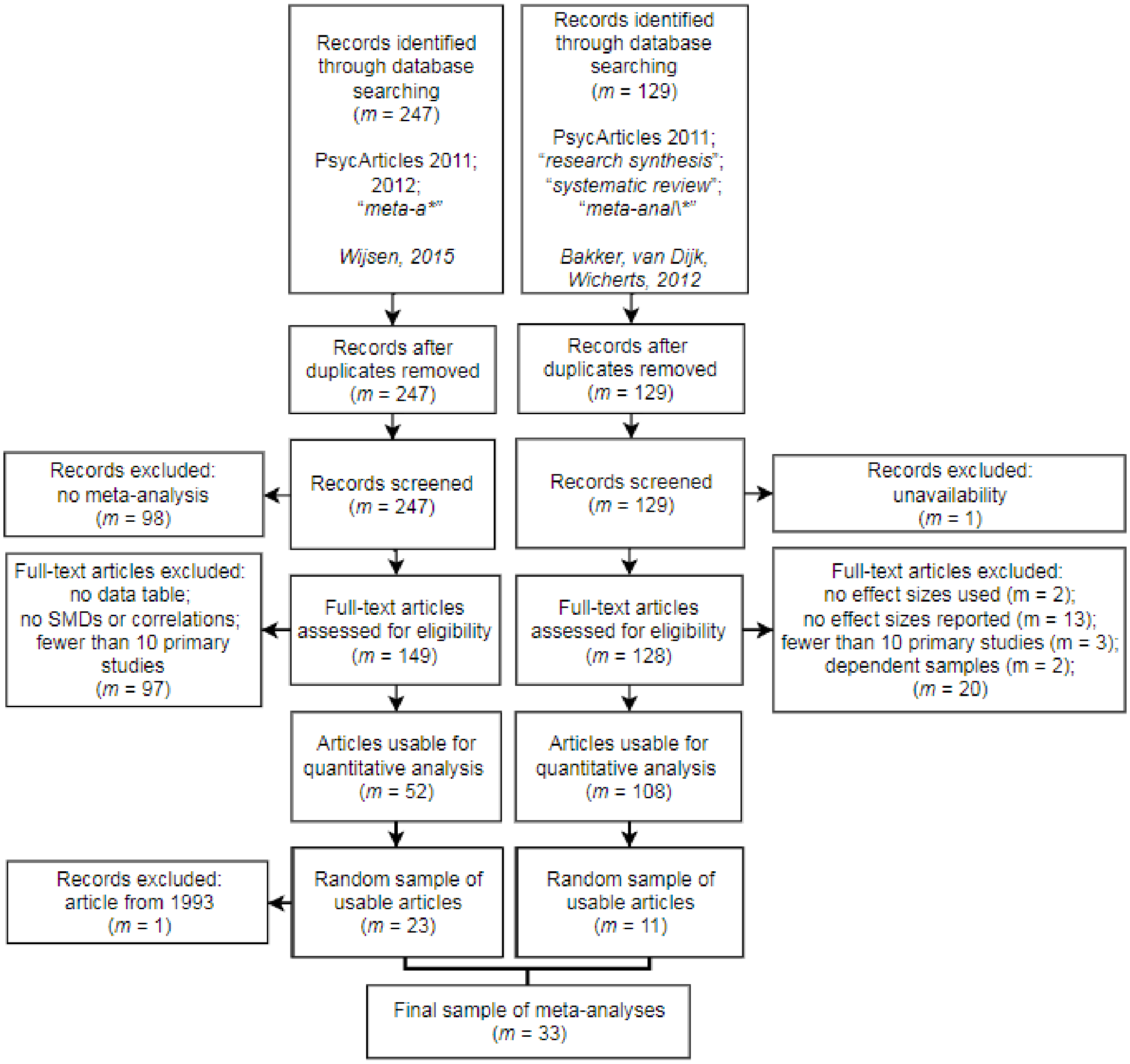


## Primary study selection

Our selection of primary studies is displayed in the flowchart above. When selecting primary studies from the meta-analyses, we wanted to sample both primary studies that could be considered outliers compared to the rest of the primary studies in the meta-analysis, as well as non-outlier primary studies. Some data errors in meta-analyses are likely to result in outliers, which inflate variance estimates (Hunter and Schmidt, 2004). Because of this, we oversampled outlier primary studies to investigate whether outliers were more likely to be erroneous compared to non-outlier primary studies. First, for each of

the 33 meta-analyses, we fitted a random-effects model in $R$ version 3.3.2 with the metafor package version 1.9-9 (**rcoreteam2018**; Viechtbauer, 2010), after which the leave-one-out function repeatedly fitted the model again, leaving out one study at a time. Outliers were detected by calculating the $Q$-statistic that is used to test the null hypothesis of homogeneity. The $Q$-statistic of the complete meta-analysis was compared to the $Q$-statistic of the complete meta-analysis minus one study. If effect sizes are homogeneous, $Q$ follows a $\chi^2$ distribution with $k$-1 degrees of freedom, where $k$ is the number of studies. Since the difference between the two random-effects models is always one study, $k$= 1. If the observed difference between the two $Q$ values exceeded the $95^{th}$ percentile of the central $\chi^2$ distribution, the left out primary study was classified as an outlier.

For all meta-analyses, we classified all primary studies as either being an outlier or non-outlier. Per meta-analysis, we first took a random sample from the collection of outlier primary studies. To ensure a fair distribution of outlier and non-outlier primary studies, we sampled 10 outlier primary studies per meta-analysis[2]. If a meta-analysis contained fewer than 10 outliers, we included all outliers. If we could not retrieve an outlier primary study due to article unavailability, we randomly selected another outlier from the same meta-analysis until none were left. In total, we included 197 primary studies that were considered outliers. After this selection, a total of 303 primary studies remained to be sampled from the non-outlier primary studies within the 33 meta-analyses. The remaining primary studies were evenly selected from the meta-analyses, meaning we randomly chose approximately 10 studies per meta-analysis. If we failed to find the article for a non-outlier primary study, we randomly selected another non-outlier primary study from the same meta-analysis. If the number of primary studies to extract exceeded the total number of primary studies in the meta-analysis, we randomly divided the remaining number of primary studies among the other meta-analyses that had studies left to be sampled.

[2]Inadvertently, in three cases, more than ten outlier primary study effect sizes were sampled; meta-analysis no. 7 and 19 have 13 non-outlier primary studies, meta-analysis no. 9 has 12 non-outlier primary studies.

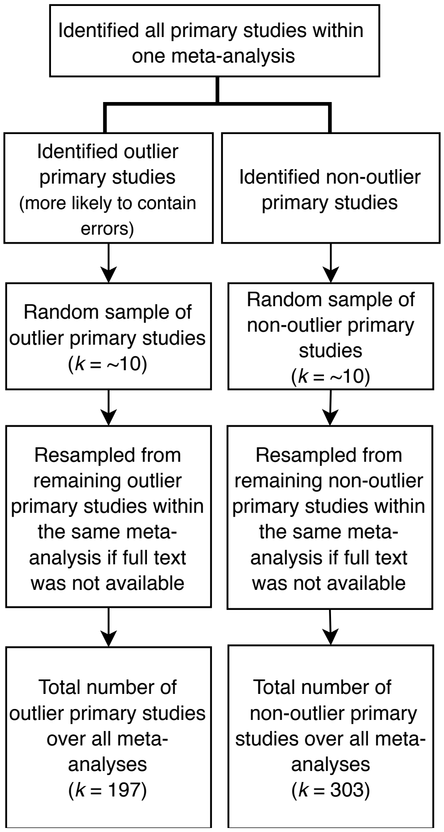


# References


Adesope, O. O., & Nesbit, J. C. (2012). Verbal redundancy in multimedia learning environments: A meta-analysis. *Journal of Educational Psychology*, *104*(1), 250–263. meta-analysis. doi:10.1037/a0026147

Alfieri, L., Brooks, P. J., Aldrich, N. J., & Tenenbaum, H. R. (2011). Does discovery-based instruction enhance learning? *Journal of Educational Psychology*, *103*(1), 1–18. meta-analysis. doi:10.1037/a0021017

Babbage, D. R., Yim, J., Zupan, B., Neumann, D., Tomita, M. R., & Willer, B. (2011). Meta-analysis of facial affect recognition difficulties after traumatic brain injury. *Neuropsychology*, *25*(3), 277–285. meta-analysis. doi:10.1037/a0021908

Bakker, M., van Dijk, A., & Wicherts, J. M. (2012). The Rules of the Game Called Psychological Science. *Perspectives on Psychological Science*, *7*(6), 543–554. doi:10.1177/1745691612459060

Balliet, D., Li, N. P., Macfarlan, S. J., & van Vugt, M. (2011). Sex differences in cooperation: A meta-analytic review of social dilemmas. *Psychological Bulletin*, *137*(6), 881–909. meta-analysis. doi:10.1037/a0025354

Benish, S. G., Quintana, S., & Wampold, B. E. (2011). Culturally adapted psychotherapy and the legitimacy of myth: A direct-comparison meta-analysis. *Journal of Counseling Psychology*, *58*(3), 279–289. meta-analysis. doi:10.1037/a0023626

Berry, C. M., Carpenter, N. C., & Barratt, C. L. (2012). Do other-reports of counterproductive work behavior provide an incremental contribution over self-reports? A meta-analytic comparison. *Journal of Applied Psychology*, *97*(3), 613–636. meta-analysis. doi:10.1037/a0026739

Berry, C. M., Clark, M. A., & McClure, T. K. (2011). Racial/ethnic differences in the criterion-related validity of cognitive ability tests: A qualitative and quantitative review. *Journal of Applied Psychology*, *96*(5), 881–906. meta-analysis. doi:10.1037/a0023222

Card, N. A., Bosch, L., Casper, D. M., Wiggs, C. B., Hawkins, S. A., Schlomer, G. L., & Borden, L. M. (2011). A meta-analytic review of internalizing, externalizing, and academic adjustment among children of deployed military service members. *Journal of Family Psychology*, *25*(4), 508–520. meta-analysis. doi:10.1037/a0024395

Crook, T. R., Todd, S. Y., Combs, J. G., Woehr, D. J., & Ketchen, D. J. (2011). Does human capital matter? A meta-analysis of the relationship between human capital and firm performance. *Journal of Applied Psychology*, *96*(3), 443–456. meta-analysis. doi:10.1037/a0022147

de Wit, F. R. C., Greer, L. L., & Jehn, K. A. (2012). The paradox of intragroup conflict: A meta-analysis. *Journal of Applied Psychology*, *97*(2), 360–390. meta-analysis. doi:10.1037/a0024844

Else-Quest, N. M., Higgins, A., Allison, C., & Morton, L. C. (2012). Gender differences in self-conscious emotional experience: A meta-analysis. *Psychological Bulletin*, *138*(5), 947–981. meta-analysis. doi:10.1037/a0027930

Farber, B. A., & Doolin, E. M. (2011). Positive regard. *Psychotherapy*, *48*(1), 58–64. meta-analysis. doi:10.1037/a0022141

Fischer, P., Krueger, J. I., Greitemeyer, T., Vogrincic, C., Kastenmüller, A., Frey, D., ... Kainbacher, M. (2011). The bystander-effect: A meta-analytic review on bystander intervention in dangerous and non-dangerous emergencies. *Psychological Bulletin*, *137*(4), 517–537. meta-analysis. doi:10.1037/a0023304

Fox, M. C., Ericsson, K. A., & Best, R. (2011). Do procedures for verbal reporting of thinking have to be reactive? A meta-analysis and recommendations for best reporting methods. *Psychological Bulletin*, *137*(2), 316–344. meta-analysis. doi:10.1037/a0021663

Freund, P. A., & Kasten, N. (2012). How smart do you think you are? A meta-analysis on the validity of self-estimates of cognitive ability. *Psychological Bulletin*, *138*(2), 296–321. meta-analysis. doi:10.1037/a0026556

Green, D., & Rosenfeld, B. (2011). Evaluating the gold standard: A review and meta-analysis of the Structured Interview of Reported Symptoms. *Psychological Assessment*, *23*(1), 95–107. meta-analysis. doi:10.1037/a0021149

Hallion, L. S., & Ruscio, A. M. (2011). A meta-analysis of the effect of cognitive bias modification on anxiety and depression. *Psychological Bulletin*, *137*(6), 940–958. meta-analysis. doi:10.1037/a0024355

Hunter, J. E., & Schmidt, F. L. (2004). *Methods of Meta-Analysis: Correcting Error and Bias in Research Findings*. New York City, NY: SAGE Publications, Inc.

Ihle, A., Bunce, D., & Kliegel, M. (2012). APOE *E*4 and cognitive function in early life: A meta-analysis. *Neuropsychology*, *26*(3), 267–277. meta-analysis. doi:10.1037/a0026769

Koenig, A. M., Eagly, A. H., Mitchell, A. A., & Ristikari, T. (2011). Are leader stereotypes masculine? A meta-analysis of three research paradigms. *Psychological Bulletin*, *137*(4), 616–642. meta-analysis. doi:10.1037/a0023557

Kolden, G. G., Klein, M. H., Wang, C.-C., & Austin, S. B. (2011). Congruence/genuineness. *Psychotherapy*, *48*(1), 65–71. meta-analysis. doi:10.1037/a0022064

Lucassen, N., Tharner, A., van IJzendoorn, M. H., Bakermans-Kranenburg, M. J., Volling, B. L., Verhulst, F. C., ... Tiemeier, H. (2011). The association between paternal sensitivity and infant–father attachment security: A meta-analysis of three decades of research. *Journal of Family Psychology*, *25*(6), 986–992. meta-analysis. doi:10.1037/a0025855

Mol, S. E., & Bus, A. G. (2011). To read or not to read: A meta-analysis of print exposure from infancy to early adulthood. *Psychological Bulletin*, *137*(2), 267–296. meta-analysis. doi:10.1037/a0021890

Morgan, R. D., Flora, D. B., Kroner, D. G., Mills, J. F., Varghese, F., & Steffan, J. S. (2012). Treating offenders with mental illness: A research synthesis. *Law and Human Behavior*, *36*(1), 37–50. meta-analysis. doi:10.1037/h0093964

Munder, T., Flückiger, C., Gerger, H., Wampold, B. E., & Barth, J. (2012). Is the allegiance effect an epiphenomenon of true efficacy differences between treatments? A meta-analysis. *Journal of Counseling Psychology*, *59*(4), 631–637. meta-analysis. doi:10.1037/a0029571

Piet, J., Würtzen, H., & Zachariae, R. (2012). The effect of mindfulness-based therapy on symptoms of anxiety and depression in adult cancer patients and survivors: A systematic review and meta-analysis. *Journal of Consulting and Clinical Psychology*, *80*(6), 1007–1020. meta-analysis. doi:10.1037/a0028329

Smith, T. B., & Silva, L. (2011). Ethnic identity and personal well-being of people of color: A meta-analysis. *Journal of Counseling Psychology*, *58*(1), 42–60. meta-analysis. doi:10.1037/a0021528

Tillman, C. M. (2011). Developmental change in the relation between simple and complex spans: A meta-analysis. *Developmental Psychology*, *47*(4), 1012–1025. meta-analysis. doi:10.1037/a0021794

Toosi, N. R., Babbitt, L. G., Ambady, N., & Sommers, S. R. (2012). Dyadic interracial interactions: A meta-analysis. *Psychological Bulletin*, *138*(1), 1–27. meta-analysis. doi:10.1037/a0025767

van Iddekinge, C. H., Roth, P. L., Putka, D. J., & Lanivich, S. E. (2011). Are you interested? A meta-analysis of relations between vocational interests and employee performance and turnover. *Journal of Applied Psychology*, *96*(6), 1167–1194. meta-analysis. doi:10.1037/a0024343

Viechtbauer, W. (2010). Conducting meta-analyses in R with the metafor package. *Journal of Statistical Software*, *36*(3), 1–48.

Webb, T. L., Miles, E., & Sheeran, P. (2012). Dealing with feeling: A meta-analysis of the effectiveness of strategies derived from the process model of emotion regulation. *Psychological Bulletin*, *138*(4), 775–808. meta-analysis. doi:10.1037/a0027600

Wijsen, L. (2015). *Do psychological effects fade away? Meta-analyzing the decline effect.* Unpublished research report. University of Amsterdam, Amsterdam, the Netherlands. https://osf.io/xswvg/.

Woodin, E. M. (2011). A two-dimensional approach to relationship conflict: Meta-analytic findings. *Journal of Family Psychology*, *25*(3), 325–335. meta-analysis. doi:10.1037/a0023791

Woodley, M. A. (2011). The cognitive differentiation-integration effort hypothesis: A synthesis between the fitness indicator and life history models of human intelligence. *Review of General Psychology*, *15*(3), 228–245. meta-analysis. doi:10.1037/a0024348

Yoon, E., Langrehr, K., & Ong, L. Z. (2011). Content analysis of acculturation research in counseling and counseling psychology: A 22-year review. *Journal of Counseling Psychology*, *58*(1), 83–96. meta-analysis. doi:10.1037/a0021128
