# Supplementary material for: Reproducibility of individual effect sizes in meta-analyses in psychology
Source: PLoS One. 2020 May 27;15(5):e0233107. doi: 10.1371/journal.pone.0233107 (PMC7252608; doi:10.1371/journal.pone.0233107)
Supplement: S3 File — https://osf.io/pqt9n/. (PDF) [file pone.0233107.s003.pdf]

Reproducibility of Individual Effect Sizes in Meta-Analyses in Psychology
Supplement C
Formulas and Methods

Supplement C contains for each meta-analysis separately the sections which contain the relevant information for effect size calculation (e.g., formulas) and meta-analytic estimation (e.g., which program was used), including the page on which the information was found. We also extracted the type of effect size, the main hypothesis, and the direction of the effect if possible. Each paragraph starting with *Note* are our comments related to the decision-making process. After our comments we state the formulas we used to calculate the effect sizes.

MA1: Adesope & Nesbit (2011)

p.253 For each study included in this meta-analysis, we obtained Cohen's d effect size, a standardized estimate of the difference in achievement scores between students who learned with spoken–written presentations compared with those who learned with either spoken-only or written-only presentations. Specifically, Cohen's d was calculated as the difference between the experimental (spoken-written presentations) and control (spoken-only or written-only presentations) mean scores divided by the pooled standard deviation of the two groups.

Because differential sample sizes across studies may bias the effect size obtained by Cohen's d, Hedges and Olkin (1985) proposed the use of Hedges's g to reduce the bias. Hedges's g (Hedges, 1981; Hedges & Olkin, 1985, p. 81) was computed and reported throughout this meta-analysis as an unbiased estimate of the standardized mean difference effect size. In a few cases where basic descriptive statistics were not provided, effect sizes were estimated from other statistics provided in the studies using conversion formulas (Cooper & Hedges, 1994; Glass, McGaw, & Smith, 1981).

Data were analyzed using Comprehensive Meta-Analysis 2.2.048 (Borenstein, Hedges, Higgins, & Rothstein, 2008) and SPSS Version 18 for Windows. The weighted mean effect sizes were aggregated to form an overall weighted mean estimate of the effect of learning with spoken–written presentations (i.e., $g+$). This approach allowed more weight to be assigned to studies with larger sample sizes.

Positive effect sizes indicate benefits of spoken–written verbal presentations over spoken-only or written-only presentations.

Note: the authors do not indicate whether they used a fixed effect or random-effects model. We estimated both. For the random-effects analysis, we used the DerSimonian and Laird (DL) estimation method in the metafor package in R, since this method is the standard estimation method in Comprehensive Meta-Analysis. When we tried to reproduce results, we assumed the meta-analytic

authors used a random-effects model.

The meta-analysis uses effect size g.

$$d = \frac{\bar{x}_1 - \bar{x}_2}{s} = \frac{\mu_1 - \mu_2}{s}, \text{ Hedges and Olkin, 1985, p.78}$$

$$s = \sqrt{\frac{(n_1 - 1)s_1^2 + (n_2 - 1)s_2^2}{n_1 + n_2 - 2}}, \text{ Hedges and Olkin, 1985, p.79}$$

$$J = 1 - \frac{3}{4N - 9}, \text{ Hedges and Olkin, 1985, p.81}$$

$$g = J \times d, \text{ Hedges and Olkin, 1985, p.81}$$

$$d = \pm\sqrt{\frac{F(n_1 + n_2)}{n_1 n_2}}, \text{ Cooper, Hedges, and Valentine, 2009, p.228}$$

$$OR = \frac{AD}{BC}, \text{ Cooper et al., 2009, p.266}$$

$$d = log(OR) \times \frac{\sqrt{3}}{\pi}, \text{ Cooper et al., 2009, p.232}$$

MA2: Alfieri et al. (2011)

p.6 Computation formulae included within the CMA program allowed for direct entry of group statistics to calculate effect sizes for each test-by-test comparison. When the only statistics available were F values and group means, DSTAT (Johnson, 1993) allowed us to convert those statistics to a common metric, g, which represents the difference in standard deviation units. More specifically, g is computed by calculating the difference of the two means divided by the pooled standard deviation of the two samples (e.g., the difference between two groups' mean reaction times, divided by the pooled standard deviation). Those g scores and other group statistics were then entered into the CMA program. For analyses at the level of studies, overall g statistics were calculated in DSTAT before entry into the CMA program. Because g values may "overestimate the population effect size" when samples are small (Johnson, 1993, p. 19), Cohen's d values are reported here as calculated by the CMA program.

Effects sizes were coded so that a negative effect size indicates that participants in the compared instructional conditions evidenced greater learning than participants in discovery conditions, whereas a positive effect size indicates that participants in the discovery conditions evidenced greater learning than participants in the compared instructional conditions.

Given the great variety of discovery learning designs and the variety of undetermined factors involved in any potential effects, a random effects model

was used in all analyses in the Comprehensive Meta-Analysis Version 2 (CMA) program (Borenstein, Hedges, Higgins, & Rothstein, 2005).

For analyses at the level of studies, overall g statistics were calculated in DSTAT before entry into the CMA program.

Note: we assumed the authors calculated Hedges' g instead of Cohen's d, since they state they corrected for small sample bias. CMA formulas can be found in Borenstein, Hedges, Higgins, and Rothstein, 2009: https://www.meta-analysis.com/pages/formulas.php. We found effects for which no transformation formulas were available- in those cases, other standard formulas from Cooper and Hedges (1994), andCohen, 1988 were used to reproduce the effect sizes. We used the DerSimonian and Laird (DL) estimation method in the metafor package in R for the random-effects analysis, since this method is the standard estimation method in Comprehensive Meta-Analysis.

The meta-analysis uses effect size g.

$$d = \frac{\bar{x}_1 - \bar{x}_2}{s} = \frac{\mu_1 - \mu_2}{s}, \text{ Borenstein et al., 2009, f.4.18}$$

$$s = \sqrt{\frac{(n_1 - 1)s_1^2 + (n_2 - 1)s_2^2}{n_1 + n_2 - 2}}, \text{ Borenstein et al., 2009, f.4.19}$$

$$J = 1 - \frac{3}{4df - 1}$$

where df for two independent groups is n1+n2-2, Borenstein et al., 2009, f.4.22

$$g = J \times d, \text{ Borenstein et al., 2009, f.4.23}$$

$$d = \pm\sqrt{\frac{F(n_1 + n_2)}{n_1 n_2}}, \text{ Cooper et al., 2009, p.228}$$

$$OR = \frac{AD}{BC}, \text{ Borenstein et al., 2009, f.5.8}$$

$$d = log(OR) \times \frac{\sqrt{3}}{\pi}, \text{ Borenstein et al., 2009, f.7.1}$$

$$d = t\sqrt{\frac{n_t + n_c}{n_t n_c}}, \text{ Cooper et al., 2009, p.228}$$

$$\phi = \sqrt{\frac{\chi^2(1)}{N}}, \text{ Cohen, 1988, f.7.2.5}$$

$$d = \frac{2r}{\sqrt{1 - r^2}}, \text{ Borenstein et al., 2009, f.7.5}$$

MA3: Babbage et al. (2011)

p.280 Standardized mean difference effect sizes were calculated for 12 of the 13 studies, using formulae 3.21 from Lipsey and Wilson (2001, p. 48). In one study (Jackson & Moffat, 1987) means and standard deviations were not reported, so the standardized mean difference was instead calculated from the F-ratio from their two-group one way analysis of variance (see Lipsey & Wilson, 2001, for the procedure.) This effect size has been demonstrated to show positive bias when based on smaller sample sizes, and in particular where the sample is fewer than 20 participants (Hedges, 1981). Given the small sample sizes in the studies examined, most of which were based on fewer than 20 participants, the use of Hedges' unbiased effect size (g) was calculated for all studies, using formulae 3.22 to 3.24 from Lipsey and Wilson (2001; see pp. 48–50, also for discussion of this correction for studies with small n).

p.278 Regardless of task, persons with TBI have been repeatedly shown to perform more poorly than healthy controls.

p.281 To determine the effect size mean and distribution, a mean weighted by the inverse variance weights was calculated. Initially a fixed effects model was examined, which assumed that all variability observed was random error only associated with participant-level sampling error (Lipsey & Wilson, 2001). However, homogeneity analysis indicated this model was not a suitable fit for the data observed (Q = 29.54, Qcrit = 22.36). Therefore, a random effects model (method of moments) was examined, allowing for variability that was beyond simple participant-sampling error, which related to random differences at a study level that could not be formally identified (see Lipsey & Wilson, 2001, pp. 115–121).

Note: we reversed the effect sizes for this meta-analysis, so positive effect sizes indicate better performance for control groups, as is hypothesized by the authors. We used the DerSimonian and Laird estimator in the metafor package in R, since this is a method of moments based approach, which coincides with the Lipsey and Wilson (2001) reference.

The meta-analysis uses effect size g.

$$d = \frac{\bar{x}_1 - \bar{x}_2}{s} = \frac{\mu_1 - \mu_2}{s}, \text{ Lipsey and Wilson, 2000, p.48}$$

$$s = \sqrt{\frac{(n_1 - 1)s_1^2 + (n_2 - 1)s_2^2}{n_1 + n_2 - 2}}, \text{ Lipsey and Wilson, 2000, p.47}$$

$$J = 1 - \frac{3}{4N - 9}, \text{ Lipsey and Wilson, 2000, p.49}$$

$$g = J \times d, \text{ Lipsey and Wilson, 2000, p.49}$$

MA4: Balliet et al. (2011)

p.887 We used the d value as the measure of effect size. The d value is the difference between two means divided by the pooled standard deviation and is corrected for sample size bias (Hedges & Olkin, 1985). The d value for each study was calculated by using the mean difference and standard deviations for men versus women, but when these descriptive statistics were unavailable we calculated d by using a t score, F score, chisquare value, or rates of cooperation. When a study included a manipulated variable, we coded the overall main effect of gender across experimental conditions.

Women were coded as 1, and men were coded as 2 so that a positive d value indicates greater cooperation by men, relative to women, whereas a negative d value is telling of greater cooperation by women compared to men. All results of resource or take-some dilemmas are reverse coded to indicate that less taking equals greater cooperation. Several articles reported a null relationship between sex and cooperation, but failed to provide the statistics necessary to calculate the effect size. We estimated that these studies had an effect size of zero.

In our analysis, we first estimate the overall effect size using a random effects model, along with both the 95% confidence interval and the 90% prediction interval.

Analyses were conducted using Hedges and Olkin's (1985) approach with the Comprehensive Meta-Analysis Software.

Note: we assumed the authors calculated Hedges' g instead of Cohen's d, since they state they corrected for small sample bias. We found effects for which no transformation formulas were available- in those cases, other standard formulas from Cooper and Hedges (1994) were used to reproduce the effect sizes. The authors do not explicitly state a hypothesized direction of the effect. They present two arguments, one in favor of women being more cooperative in same sex interactions, one in favor of men being more so. For mixed sex interactions, the authors tend to slightly lean towards women being more cooperative. Since we did not want to deviate from the original meta-analysis if not needed, we decided to not reverse code the effect, so a positive effect indicates men being more cooperative than women. For the random-effects analysis, the authors state they used the Hedges & Olkin (HE) approach with the Comprehensive Meta-Analysis (CMA) software, but CMA only offers a DerSimonian-Laird (DL) or Maximum Likelihood (ML) estimator. The difference between the HE and DL methods (which are both method of moments estimators) is that HE is based on the uneweighted variance of treatment effect estimates, whereas DL is based on their weighted variance (Veroniki et al. (2016)). Since the DL method is the standard estimation method in CMA, and it resembles the HE method, we used the DL estimator in the metafor package in R.

The meta-analysis uses effect size g.

$$d = \frac{\bar{x}_1 - \bar{x}_2}{s} = \frac{\mu_1 - \mu_2}{s}, \text{ Hedges and Olkin, 1985, p.78}$$

$$s = \sqrt{\frac{(n_1 - 1)s_1^2 + (n_2 - 1)s_2^2}{n_1 + n_2 - 2}}, \text{ Hedges and Olkin, 1985, p.79}$$

$$J = 1 - \frac{3}{4N - 9}, \text{ Hedges and Olkin, 1985, p.81}$$

$$g = J \times d, \text{ Hedges and Olkin, 1985, p.81}$$

$$d = \pm\sqrt{\frac{F(n_1 + n_2)}{n_1 n_2}}, \text{ Cooper et al., 2009, p.228}$$

$$OR = \frac{AD}{BC}, \text{ Cooper et al., 2009, p.266}$$

$$d = log(OR) \times \frac{\sqrt{3}}{\pi}, \text{ Cooper et al., 2009, p.232}$$

$$d = t\sqrt{\frac{n_t + n_c}{n_t n_c}}, \text{ Cooper et al., 2009, p.228}$$

$$d = \frac{2r}{\sqrt{1 - r^2}}, \text{ Borenstein et al., 2009, f.7.5}$$

$$r_{y1} = \beta_1 + r_{12}(r_{y2} - \beta_1 r_{12}), \text{ Peterson and Brown, 2005, p.177}$$

MA5: Benish et al. (2011)

p.283 The effect sizes (Cohen's d) and variance were calculated for each outcome measure, within each study before aggregating across outcome variables within each treatment, using standard meta-analytic procedures outlined by Hedges and Olkin (1985). If a primary study included more than two bona fide treatments, separate effect sizes were computed between comparisons of a culturally adapted treatment (Tx A) to unadapted treatments (i.e., A to B and A to C). Each direct comparison of culturally adapted psychotherapy to unadapted bona fide treatment provided one aggregate effect size in the analysis.

A random-effects model was used for the analysis, under the assumption that studies were sampled from a larger population of studies (Hedges & Olkin, 1985). Effect sizes were calculated in the standard manner by subtracting the mean of the unadapted treatments from the mean of the adapted treatment (and scaling so that a positive effect indicated superiority of the adapted treatment) and dividing by the pooled standard deviation. These effects then were corrected for bias (Hedges & Olkin, 1985).

p.282 The analysis of primary measures was conducted following an analysis incorporating all outcome measures, including both primary and secondary measures. Due to the fact that data from multiple outcome measures of the

same clients are not independent, the data from outcome measures were aggregated to account for dependence using the methods described by Wampold et al., 1997.

Note: we assumed the authors calculated Hedges' g instead of Cohen's d, since they state they corrected for (small sample) bias. We used the Hedges estimator in the metafor package in R, which is referenced in Hedges and Olkin (1985).

The meta-analysis uses effect size g.

$$d = \frac{\bar{x}_1 - \bar{x}_2}{s} = \frac{\mu_1 - \mu_2}{s}, \text{ Hedges and Olkin, 1985, p.78}$$

$$s = \sqrt{\frac{(n_1 - 1)s_1^2 + (n_2 - 1)s_2^2}{n_1 + n_2 - 2}}, \text{ Hedges and Olkin, 1985, p.79}$$

$$J = 1 - \frac{3}{4N - 9}, \text{ Hedges and Olkin, 1985, p.81}$$

$$g = J \times d, \text{ Hedges and Olkin, 1985, p.81}$$

$$d = \pm\sqrt{\frac{F(n_1 + n_2)}{n_1 n_2}}, \text{ Cooper et al., 2009, p.228}$$

$$var_d = \frac{(\frac{(n_c+n_t)}{n_c n_t})}{(\frac{d^2}{2\times(n_c+n_t)})}, \text{ Wampold et al., 1997, f.3}$$

$$d_{agg} = \frac{\Sigma(\frac{d}{var_d})}{\Sigma(\frac{1}{var_d})}, \text{ Wampold et al., 1997, f.6}$$

MA6: Berry, Carpenter, & Barrat (2012)

p.618 For each sample, the correlation between (a) self- and other-reports of CWB and/or (b) the correlation between other-reports of CWB and CWB correlates was coded. In cases in which relationships with the overall CWB construct were of interest but multiple facets of the overall construct were offered within the same sample (e.g., both interpersonal- and organizational target CWB measures, which are multiple facets of overall CWB, listed for the same sample), composite formulas (Ghiselli, Campbell, & Zedeck, 1981, pp. 163–164) were used to estimate correlations with a composite of the multiple measures. The mean and standard deviation of CWB reported by self- and other-raters was also coded for use in calculating standardized mean differences in CWB between self- and other-ratings. It should be noted that an explicit search for studies

reporting mean differences between self- and other-reported CWB was not carried out; instead, mean differences were coded in the correlational studies that provided relevant means and standard deviations.

Hunter and Schmidt's (2004) meta-analysis methods were used for meta-analyses of correlations and d values. Corrections for two statistical artifacts were made. First, point-biserial correlations involving the variable "gender" were individually corrected to what they would be if each sample had a 50–50 gender split (see Table A2 for gender splits for samples). Second, correlations and d values were corrected for unreliability in both the predictor and the criterion using the artifact distribution method (see Table 2 for predictor and criterion reliability artifact distributions and their sources).

p.619, footnote 3 So, for the sake of comparability with the previous meta-analyses in which alpha coefficients were used in CWB corrections, the results in our tables are based on the correlations corrected using alpha coefficients, and we mostly focus our discussion on those estimates.

p.615 Hypothesis 1: The correlation between self- and other-ratings of CWB will be positive and greater than zero but will not reach unity.

Note: we were unable to locate the Ghiselli et al. text. We took the average when multiple measures were combined and we could not find any of the inter-correlations between the CWB-O and CWB-I measures; this is the same strategy used in the next meta-analysis (which has the same first author). In our sample, there were no instances where multiple measures had to be combined and inter-correlations between constructs were available. We did make corrections in the correlations using the artifact distribution method. The authors do not indicate whether they used a fixed effect or random-effects model. We estimated both. For the random-effects meta-analysis, we used the Hunter & Schmidt estimation method in the metafor package in R.

The meta-analysis uses effect size r.

$$a = \sqrt{r_{xx}}, b = \sqrt{r_{yy}}, \text{ Hunter and Schmidt, 2004, p.150}$$

$$r_{corrected} = \frac{r_{uncorrected}}{a \times b}, \text{ Hunter and Schmidt, 2004, p.151}$$

MA7: Berry, Clark, & McClure (2011)

p.887 For each independent sample, the correlation between the cognitive ability test and performance criterion was coded, along with the racial/ethnic subgroup and sample size. If multiple cognitive ability tests (e.g., SAT Verbal and SAT Mathematical scores) and/or multiple related performance criteria (e.g., subjective performance ratings and an objective performance index) were included within a single sample, composite formulas (Ghiselli, Campbell, & Zedeck, 1981, pp. 163–164) were used to estimate the correlation between a composite of the multiple tests and/or the multiple criterion measures when intercorrelations among multiple predictors and/or criteria were provided. If intercorrelations

were not provided, the multipredictor– criterion correlations were combined by averaging predictor– criterion correlations across the multiple tests/criteria.

p.889 Formulas presented by Hunter and Schmidt (2004) were used to calculate meta-analytic mean correlations, standard deviations, and percentage of variance due to sampling error. Additionally, confidence intervals around mean correlations were calculated with formulas provided by Whitener (1990).

Note: we were unable to locate the Ghiselli et al. text. When multiple measures had to be combined and intercorrelations between constructs were available, we used the Hunter and Schmidt method (2004, p.435) to aggregate. The authors do not explicitly state a hypothesized direction of the effect. However, cognitive ability test scores are generally assumed to correlate positively with performance, and as such we decided to not recode the effect sizes. The authors do not indicate whether they used a fixed effect or random-effects model. We estimated both. For the random-effects meta-analysis, we used the Hunter & Schmidt estimation method in the metafor package in R.

The meta-analysis uses effect size r.

$$r_{aggregated} = \frac{\sum r_{xy}}{\sqrt{n + n(n-1)\bar{r}_{xy}}}, \text{ Hunter and Schmidt, 2004, p.435}$$

where n is the number of correlations to aggregate and

$\bar{r}_{xy}$ is the intercorrelation, Hunter and Schmidt, 2004, p.435

MA8: Card et al. (2011)

p.511 For this meta-analysis, we used the correlation coefficient,r, as an effect size that represents the association between deployment and children's adjustment. Positive values indicate that children of deployed parents have more problems than controls, whereas negative values denote that these children had fewer problems than controls.

p.512 We coded the effect sizes from included studies by either recording the correlations reported or computing these correlations from other reported data using common meta-analysis equations (see Card, 2011; Rosenthal, 1991).

Given that the correlation coefficient is skewed in the population, we transformed these effect sizes via Fisher's transformation to obtain *Zr*. Analyses were performed by using this transformed effect size. Results (e.g., average *Zr*) were backtransformed to the more familiar r for reporting.

The general analytic strategy for each of the meta-analyses reported below was to first compute the weighted random-effects mean effect size of the association between deployment and adjustment (note that the random-effects model would simplify to the fixed-effects model in the absence of heterogeneity; for information about these models see Card, in press; Hedges & Vevea, 1998 Raudenbush, 1994).

Note: we found effects for which no transformation formulas were available- in those cases, other standard formulas from Cooper and Hedges (1994) were

used to reproduce the effect sizes. The authors refer to multiple sources that describe random-effects models, but it is unclear which estimation method they used. The first estimation method referenced in both Hedges and Vevea (1998) and Raudenbush (1994) is a method of moments estimation. As such, we decided to use the DerSimonian-Laird estimator method in the metafor package in R.

The meta-analysis uses effect size r.

$$d = \frac{\bar{x}_1 - \bar{x}_2}{s} = \frac{\mu_1 - \mu_2}{s}, \text{ Card et al., 2011, p.90}$$

$$s = \sqrt{\frac{(n_1 - 1)s_1^2 + (n_2 - 1)s_2^2}{n_1 + n_2 - 2}}, \text{ Card et al., 2011, p.124}$$

$$r = \frac{d}{\sqrt{d^2 + a}}, \text{ Cooper et al., 2009, p.234}$$

$$a = \frac{(n_1 + n_2)^2}{n_1 n_2}, \text{ Cooper et al., 2009, p.234}$$

$$Z_r = \frac{1}{2} \log \frac{(1 + r)}{(1 - r)}, \text{ Cooper et al., 2009, p.231}$$

$$r = \tanh(Z_r)$$

MA9: Crook et al. (2011)

p.446 Because the study is the unit of analysis, if a study used multiple measures of one or more human capital measures or one or more performance measures and reported correlations separately, the correlations were averaged to yield a single estimate for the study (Hunter & Schmidt, 2004).

Effect size estimates were calculated as the mean of the sample size weighted correlations ($\bar{r}$) from primary studies. This estimate offers more accuracy than estimates obtained from any one study, because positive and negative sampling errors cancel out (Hunter & Schmidt, 2004). After sampling error, measurement error has the largest impact on study findings. Unfortunately, most human capital studies do not report reliability coefficients, making it impossible to correct each study individually for measurement error. Thus, we used the mean of the available reliabilities to correct $\bar{r}$ (i.e., $\bar{r}_c$) according to formulas offered by Hunter and Schmidt (2004). In particular, the average reliability for human capital ($\bar{r}_{xx}$) is .81, and the average reliability for performance ($\bar{r}_{yy}$) is .91. Following Hunter and Schmidt, we corrected $\bar{r}$ according to:

$$\bar{r}_c = \frac{\bar{r}}{\sqrt{\bar{r}_{xx}}\sqrt{\bar{r}_{yy}}}$$

wherein we took the product of the square roots of the available reliabilities. Thus, we used .82 to correct $\bar{r}$.

p.444 Hypothesis 1: Human capital is positively related to performance.

p.450 All chi-square statistics are significant; thus, we assume residual variance is heterogeneous for all results.

Note: the authors do not indicate whether they used a fixed effect or random-effects model. We estimated both. We used no specific formulas for this meta-analysis, since only single correlations or the mean of multiple correlations were extracted or calculated. To get an overall estimate, we took the mean of the sample size weighted correlations. This estimate is used as an overall effect size. Afterwards we attenuated for measurement error by dividing this estimate by the square root of the two reported reliability estimates of human capital and performance. The authors report this estimate to be 0.82, but if we follow the printed formula we multiply the square root of 0.81 with the square root of 0.91, making it 0.86. We will be using this latter estimate in our calculations for the confidence interval. Since the authors find statistically significant chi-square result, they assume the studies are heterogeneous, meaning they calculate the following standard error for the confidence interval from Whitener, 1990:

$$SE = ([(1 - \bar{r}^2)^2/(N - K)] + (SD^2_{res}/K))^{1/2}$$

where $\bar{r}$ is the sample-size weighted mean uncorrected correlation, N is the total sample size, K is the number of studies, and SD^2_{res} is the residual variance of the observed correlations after the variance for sampling error has been removed. This variance has not been corrected for other artifacts such as measurement error or range restriction:

Whitener (1990, p.316). The confidence interval generated using the standard error reflects the effects of sampling error and is therefore applied to sample-size weighted mean effect sizes that have not been corrected for research artifacts. The estimation of confidence intervals in a meta-analysis involves two steps. First, the sample-size weighted mean effect size and variance are calculated. Second, confidence intervals are generated around the uncorrected, sample-size weighted mean effect size using the standard error of the mean effect size.

Note: we calculated the variance for sampling error from Hunter & Schmidt, 2004, page 15:

$$S^2_e = \frac{\Sigma_{i=1}[N_i(1 - \bar{r}^2)^2/N_i - 1]}{\Sigma N_i}$$

The meta-analysis uses effect size r.

MA10: de Wit et al. (2012)

p.366 All the effect sizes were first corrected for sampling error. Next, we corrected for the measurement error in the independent and dependent variables. This was done according to the approach developed by Hunter and Schmidt

(1990, 2004); we divided individual effect sizes by the square root of the reliability estimates of the two correlated variables. We used internal consistency coefficients reported in the respective study as the reliability estimates. In case the authors did not report internal consistency coefficients, the internal consistency coefficient for each variable across all studies included in the meta-analysis was used. We assigned a reliability coefficient of 1.00 to objective performance indicators for which no reliability coefficient was reported (for similar procedures, see, e.g., Riketta, 2008). In case a study provided multiple estimates of a correlation between a predictor (X) and a criterion (Y), we used the formula for composites (Hunter & Schmidt, 2004) to derive a linear composite of the effect sizes to ensure the independence of effects sizes in the final data set.

The analyses were conducted using the Schmidt-Le program (Version 1.1; Schmidt & Le, 2004). The precision of the effect sizes was examined by calculating the 95% confidence interval (CI) around the effect size. Finally, we used the procedures described by Viechtbauer and Cheung (2010) to derive outlier and influence diagnostics, using the Metafor meta-analysis package for R (Version 1.4-0; Viechtbauer, 2010a, 2010b).

p.364 Hence, irrespective of the task at hand, we expect relationship and process conflict to interfere with group functioning and to be negatively related to both proximal and distal group outcomes (e.g., Jehn, 1995).

Note: the authors state they correct the effect sizes for sampling error, but do not state how- we did not make any corrections in our reproduced effect sizes related to sampling error. We did correct for measurement error if internal consistency estimates coefficients were reported. We aggregated effect sizes when multiple estimates were found, for which we used the Hunter and Schmidt method (2004, p.435). Since the authors expect relationship conflict to be negatively correlated with performance and other group outcomes, we inversed all effect sizes so that a positive effect size is in line with the expectation of the meta-analysts. The authors do not indicate whether they used a fixed effect or random-effects model. We estimated both. For the random-effects meta-analysis, we used the Hunter & Schmidt estimation method in the metafor package in R, since methods from Hunter and Smith (2004) are used in the Schmidt-Le software.

The meta-analysis uses effect size r.

$$a = \sqrt{r_{xx}}, b = \sqrt{r_{yy}}, \text{ Hunter and Schmidt, 2004, p.150}$$

$$r_{corrected} = \frac{r_{uncorrected}}{a \times b}, \text{ Hunter and Schmidt, 2004, p.151}$$

$$r_{aggregated} = \frac{\sum r_{xy}}{\sqrt{n + n(n-1)\bar{r}_{xy}}}$$

where n is the number of correlations to aggregate and
$\bar{r}_{xy}$ is the intercorrelation, Hunter and Schmidt, 2004, p.435

MA11: Else-Quest et al. (2012)

p.952 Formulae for the effect size and homogeneity tests were taken from JacobCohen, 1988 and Lipsey and Wilson (2001). The effect size d was computed by subtracting the mean score for women from the mean score for men, divided by the within-groups standard deviation. Means and standard deviations were available for 571 of the 697 effects. For 126 of the effects, other usable statistics (e.g., Pearson correlations, t tests, F tests) were provided or obtained. These were converted to d according to the formulae provided by Cohen. Positive values of d represent higher scores for men than women, whereas negative values represent higher scores for women.

p.951 Parallel to consistent empirical findings in gendered emotion stereotypes, gender role socialization, and gender differences in self-esteem (general and domain specific) and psychopathology, we predicted greater pride (both hubristic and authentic) in men than in women, as well as greater shame, guilt, and embarrassment in women than in men.

p.952 Uncorrected effect sizes for shame, guilt, embarrassment, authentic pride, and hubristic pride appear in Tables 1, 2, 3, 4, and 5, respectively, along with corresponding study information. For the estimation of population effect sizes, all effect sizes were corrected for bias with the formula provided by Hedges and Becker (1986).

Note: the formula for the pooled standard deviation is slightly different in Cohen (1988, f.2.5.2) and we do not have acces to the raw data, so cannot use that formula. Since we wanted to reproduce the effect sizes of authentic pride from Table 4, which are uncorrected, we attempted to calculate Cohen's d, and we did not correct for small sample bias. Since we are not interested in moderators, we estimated a random-effects model for the meta-analytic estimates. We used the DerSimonian and Laird estimator in the metafor package in R, which coincides with the Lipsey and Wilson (2001) reference.

The meta-analysis uses effect size d.

$$d = \frac{\bar{x}_1 - \bar{x}_2}{s} = \frac{\mu_1 - \mu_2}{s}, \text{ Lipsey and Wilson, 2000, p.48}$$

$$s = \sqrt{\frac{(n_1 - 1)s_1^2 + (n_2 - 1)s_2^2}{n_1 + n_2 - 2}}, \text{ Lipsey and Wilson, 2000, p.47}$$

$$\phi = \sqrt{\frac{\chi^2(1)}{N}}, \text{ Cohen, 1988, f.7.2.5}$$

$$d = \frac{r_{pb}}{\sqrt{p(1-p)(1-r_{pb}^2)}}$$

where p is the propotion of subjects in group 1, and 1-p is the proportion of subjects in group 2, Lipsey and Wilson, 2000, p.62

$$d = 2\sqrt{\frac{F}{N}}, \text{ Lipsey and Wilson, 2000, p.174}$$

MA12: Farber & Doolin (2011)

p.61 Because the purpose of this meta-analysis was to examine the relation between therapist positive regard and treatment outcome, a simple correlation, r, was obtained to measure the effect for each study. The effect sizes for several studies had to be recomputed using the data the authors provided and then converted to r (per Cooper, Hedges, & Valentine, 2009). After each study was coded for the moderator variables, effect sizes were again computed for each of the 18 studies. Additionally, if there was more than one effect size per study, within-study aggregation was performed (see Del Re, 2010; Del Re & Hoyt, 2010).

The aggregate effect size was .27 (p <.000; N = 1067), indicating that positive regard has a moderate association with psychotherapy outcomes; only two of the 18 studies had negative effect sizes. This number represents the random, weighted effect.

Note: we found effects for which no transformation formulas were available- in those cases, we used other standard formulas fromCohen, 1988. We used the the Hunter and Schmidt method (2004, p.435) to aggregate, which is used in the MAc package in R, which the authors reference. The authors do not explicitly state a hypothesized direction of the effect. However, positive regard (or warmth) is generally assumed to correlate positivily with therapeutic outcome, and as such we decided to not recode the effect sizes. Since the authors do not reference what kind of software or estimator they use to estimate the random-effects model, we took the standard DerSimonian-Laird estimator in the metafor package in R, which is often used as the standard estimator in software.

The meta-analysis uses effect size r.

$$r = \pm\sqrt{\frac{t^2}{t^2 + n - 2}}, \text{ Cooper et al., 2009, p.233}$$

$$\phi = \sqrt{\frac{\chi^2(1)}{N}}, \text{Cohen, 1988, f.7.2.5}$$

$$r_{aggregated} = \frac{\sum r_{xy}}{\sqrt{n + n(n-1)\bar{r}_{xy}}}$$

where n is the number of correlations to aggregate and
$\bar{r}_{xy}$ is the intercorrelation, Hunter and Schmidt, 2004, p.435

$$r_{y1} = \beta_1 + r_{12}(r_{y2} - \beta_1 r_{12}), \text{ Peterson and Brown, 2005, p.177}$$

MA13: Fischer et al. (2011)

p.523 The effect size g recommended by Hedges and Becker (1986) was used in the statistical analysis. All effect sizes were computed via the statistics program Comprehensive Meta-Analysis, Version 2.2.048 (Borenstein, Hedges, Higgins, & Rothstein, 2005). We used a fixed effect model to assess the heterogeneity in different subsets of studies. Fixed effect models are sensitive to the number of participants within each study, so that studies with low sample size but extreme effects have less impact on the results of the meta-analysis. This was important to us to ensure that the test of our hypothesis that dangerous emergencies reduce the bystander effect is not biased by single studies with extreme effect sizes. Because fixed effect models assume a common true effect that underlies all studies, we also performed random-effects analyses to examine the critical variable of level of danger (high vs. low). Overall, it turned out that use of fixed or random effects models did not qualify the main findings.

If means and standard deviations were reported in the research studies, we computed the index g by subtracting the mean for the control group (no bystander present) from the mean for the experimental group (bystander present group) and divided the difference by the pooled within-group standard deviation. Hence, a negative sign indicated an inhibitory effect of bystanders on helping behavior. When no means and standard deviations were reported, we estimated g from t, F, or p values following the procedures recommended by Hedges and Becker (1986). If there was more than one measure of helping, we computed means as a composite measure.

We used a fixed effect model to assess the heterogeneity in different subsets of studies. Because fixed effect models assume a common true effect that underlies all studies, we also performed random-effects analyses to examine the critical variable of level of danger (high vs. low). Overall, it turned out that use of fixed or random effects models did not qualify the main findings.

p.522 We expected that dangerous emergencies would be associated with increased levels of emergency awareness (triggered by increased perceived costs of intervention), increased perceived costs of non-intervention, as well as increased expected physical support by other bystanders, which should altogether then reduce the bystander effect.

Note: we were unable to locate the Hedges and Becker text, and used formulas from the Comprehensive Meta-Analysis program. We found effects for which no transformation formulas were available- in those cases, other standard formulas from Cooper and Hedges (1994) were used to reproduce the effect sizes. We reversed the effect sizes for this meta-analysis, so positive effect sizes indicate bystanders reduce helping responses, as is hypothesized by the authors. The authors estimated both fixed effect as random-effects models, so we also estimated both. For the random-effects analysis, we used the DerSimonian and Laird (DL) estimation method in the metafor package in R, since this method is the standard estimation method in Comprehensive Meta-Analysis.

The meta-analysis uses effect size g.

$$d = \frac{\bar{x}_1 - \bar{x}_2}{s} = \frac{\mu_1 - \mu_2}{s}, \text{ Borenstein et al., 2009, f.4.18}$$

$$s = \sqrt{\frac{(n_1 - 1)s_1^2 + (n_2 - 1)s_2^2}{n_1 + n_2 - 2}}, \text{ Borenstein et al., 2009, f.4.19}$$

$$J = 1 - \frac{3}{4df - 1}$$

where *df* for two independent groups is n1+n2-2, Borenstein et al., 2009, f.4.22

$$g = J \times d, \text{ Borenstein et al., 2009, f.4.23}$$

$$OR = \frac{AD}{BC}, \text{ Cooper et al., 2009, p.266}$$

$$d = log(OR) \times \frac{\sqrt{3}}{\pi}, \text{ Cooper et al., 2009, p.232}$$

$$r = \frac{Z}{\sqrt{N}}, \text{ Rosenthal and DiMatteo, 2001, p.72}$$

$$r = \frac{d}{\sqrt{d^2 + a}}, \text{ Cooper et al., 2009, p.234}$$

$$a = \frac{(n_1 + n_2)^2}{n_1 n_2}, \text{ Cooper et al., 2009, p.234}$$

MA14: Fox et al. (2011)

p.323 Studies were included only if they provided information that allowed effect sizes to be calculated. This includes (Rosenthal, 1994) descriptive statistics (means and standard deviations), F or t ratios, p values, and other nonparametric test statistics such as chisquare.

p.328 Effect sizes were converted to rs, as recommended by Rosenthal (1994). These values were adjusted for unequal sample sizes to correct for sampling error, on the basis of Hunter and Schmidt (2004), and were Fisher Z-transformed for analyses. The effect sizes were converted back to report results. Effect sizes reflecting higher performance in verbal report conditions were assigned positive values, and effect sizes reflecting lower performance were assigned negative values.

All studies were weighted by sample size using the Z_r formula $w_i = n_i - 3$.

p.329 A mixed effects approach consisting of both fixed and random effects was used for analyses (Lipsey & Wilson, 2001).

Random effects were essential in this case because the sample includes a variety of tasks, and the purpose of the analysis is to provide generalizable insights

about reactivity of verbalization. Mixed effects allow moderator analyses to be conducted on heterogeneous samples (Lipsey & Wilson, 2001).

The entire data set was initially analyzed with fixed effects to obtain a heterogeneity estimate (Q). An additional calculation of I^2 provides a standardized indicator of heterogeneity by taking k into account (Higgins, Thompson, Deeks, & Altman, 2003). Rejection of homogeneity means that moderator analysis is warranted because there is too much between-studies variance to be accounted for by chance alone (Hedges & Olkin, 1985). Moderator analyses were conducted with Lipsey and Wilson's (2001) mixed effects analogues to the analysis of variance (ANOVA) and regression (meta-ANOVA and metaregression), using iterative maximum likelihood for parameter estimation.

Note: we found effects for which no transformation formulas were available- in those cases, other standard formulas from Cooper and Hedges (1994) and other references were used to reproduce the effect sizes. The authors present the estimate of a random-effects model in Table 2 (see note a below the table). As such, we tried to reproduce the meta-analytic effect using a random-effects model. The authors do not explicitly state a hypothesized direction of the effect. However, they base their research on the think-aloud effect on performance on the Ericsson and Simon (1980, 1984) model in which is argued that thinking aloud should not alter or disrupt the processes that mediate task performance. In other words, verbalization should not worsen performance. The goal of this meta-analytic study is to provide insight in which types of verbalization procedures are minimally reactive. Following this reasoning, either a null effect, or a positive effect for thinking aloud is expected, since a negative effect (i.e., being silent is better for performance) is not expected. We did not reverse the primary study effects. For the random-effects analysis, we used the Maximum Likelihood estimation method in the metafor package in R, since the authors use this method to estimate additional moderator effects in a mixed effects model.

The meta-analysis uses effect size r.

$$d = \frac{\bar{x}_1 - \bar{x}_2}{s} = \frac{\mu_1 - \mu_2}{s}, \text{ Cooper et al., 2009, p.226}$$

$$s = \sqrt{\frac{(n_1 - 1)s_1^2 + (n_2 - 1)s_2^2}{n_1 + n_2 - 2}}, \text{ Cooper et al., 2009, p.226}$$

$$r = \frac{d}{\sqrt{d^2 + a}}, \text{ Cooper et al., 2009, p.234}$$

$$a = \frac{(n_1 + n_2)^2}{n_1 n_2}, \text{ Cooper et al., 2009, p.234}$$

$$d = \pm\sqrt{\frac{F(n_1 + n_2)}{n_1 n_2}}, \text{ Cooper et al., 2009, p.228}$$

$$OR = \frac{AD}{BC}, \text{ Cooper et al., 2009, p.266}$$

$$d = log(OR) \times \frac{\sqrt{3}}{\pi}, \text{ Cooper et al., 2009, p.232}$$

$$r_{pb} = 1 - \frac{2U}{n_e n_c}, \text{ DeCoster, 2009, f.5.15, p.18}$$

$$r_b = \frac{r_{pb}\sqrt{n_e n_c}}{|z * |(n_e n_c)}$$

where $z*$ is the point on the normal distribution with a p-value of:

$$\frac{n_e}{n_e + n_c}, \text{ DeCoster, 2009, f.5.10, p.17}$$

$$r = \frac{Z}{\sqrt{N}}, \text{ Rosenthal and DiMatteo, 2001, p.72}$$

$$r_c = \frac{ar}{\sqrt{[(a^2 - 1)r^2 + 1]}}, \text{ Hunter and Schmidt, 2004, f.7.17, p.280}$$

$$a = \sqrt{\frac{.25}{pq}}, \text{ Hunter and Schmidt, 2004, f.7.17, p.280}$$

where p and q are the proportions of the complete sample for the control and treatment group, respectively; Hunter and Schmidt, 2004, f.7.18

$$Z_r = \frac{1}{2}\log\frac{(1 + r)}{(1 - r)}, \text{ Cooper et al., 2009, p.231}$$

$$r = \tanh(Z_r)$$

MA15: Freund & Kasten (2012)

p.303 Since the included studies investigated the relationship between self-estimated and psychometrically assessed cognitive ability, most results were directly reported as correlation coefficients.

p.304 It is usually recommended to use the Fisher's z-transformed correlation coefficients in meta-analysis because their distribution is more normal than that of the Pearson correlation coefficients (Borenstein et al., 2009; Silver & Dunlap, 1987) and because the variance of the estimates of the correlation coefficients is not independent of the population parameter, ρ. Figure 1 shows that the 154 correlation coefficients appear to be relatively normally distributed. Using the nontransformed correlation coefficients offers the advantage that the results of subsequent moderator analyses can be directly interpreted in the original metric. We therefore decided to perform all analyses on the correlation coefficients. However, in order to check for the robustness of this decision, we

also report the meta-analytically derived result for the overall relationship based on the Fisher's z-transformed coefficients.

p.300 Most studies investigating the relationship between self-estimates of cognitive ability and psychometric test scores report significant, positive correlations. In 1982, Mabe and West reported an average effect size of $r = .34$ (out of 12 effect sizes). We therefore expect to find a significant, positive overall relationship between the two variables.

p.308 We conducted a random-effects meta-analysis where most studies in our data set report more than one effect size. In such cases, the main experimental settings are usually the same or at least very similar. In order to cope with any dependencies among these effect sizes, we used the hierarchical linear modeling (HLM) approach to meta-analysis (Raudenbush & Bryk, 2002).

Technically, the unconditional, or 'empty', model is a random-effects model, while the conditional model is a mixed-effects model because it includes fixed effects for the moderator variables in addition to the random components.

All analyses were conducted with the software HLM 6.08 (Raudenbush, Bryk, Cheong, & Congdon, 2009).

Note: we used no specific formulas for this meta-analysis, since only single correlations were extracted. We are trying to reproduce the average effect size from the unconditional multilevel model, which the authors specify as a random-effects model. As such, we used a function to fit meta-analytic multivariate/multilevel models without any moderators or specifications. We used the REML estimator in the metafor package in R, since REML is the default estimation method in HLM. The authors also present results when transformed to Fisher's z, which we did not try to reproduce, since their primary outcome seems to be estimates in correlation r.

The meta-analysis uses effect size r.

MA16: Green & Rosenfeld (2011)

p.97 The two types of effect sizes calculated were standardized mean differences between groups (Cohen's d) and classification accuracy (sensitivity and specificity). For consistency, Cohen's d for each study was calculated based on the available raw data (rather than the effect sizes reported in the studies).For studies that permitted multiple comparisons by including more than one control group and/or feigning group, the sample size for each duplicated group was divided in half before composite effect sizes were calculated to ensure accurate weighting of each study. Study effect sizes were expected to differ significantly by design and sample types. Therefore, composite effect sizes were calculated with random-effects models.

Data were analyzed with the Comprehensive Meta-Analysis (Version 2) program from Biostat.

Note: the authors do not reference any formulas they have used. As such, we used formulas from the Comprehensive Meta-Analysis program. The authors

do not explicitly state a hypothesized direction of the effect. However, since the comparison is made between controls and (simulated) malingeres on SIRS total score, which is expected to detect malingering, we assume the effect size is expected to be positive. We did not reverse the effect sizes. For the random-effects analysis we used the DerSimonian and Laird (DL) estimation method in the metafor package in R, since this method is the standard estimation method in Comprehensive Meta-Analysis.

The meta-analysis uses effect size d.

$$d = \frac{\bar{x}_1 - \bar{x}_2}{s} = \frac{\mu_1 - \mu_2}{s}, \text{ Borenstein et al., 2009, f.4.18}$$

$$s = \sqrt{\frac{(n_1 - 1)s_1^2 + (n_2 - 1)s_2^2}{n_1 + n_2 - 2}}, \text{ Borenstein et al., 2009, f.4.19}$$

MA17: Hallion & Ruscio (2011)

p.944 Effect sizes were determined using group means and standard deviations; t, F, or chi-square values from between-group analyses; precise p values and degrees of freedom from between-group analyses; or other effect size values (e.g., correlation coefficients) reported in the text (Borenstein, Hedges, Higgins, & Rothstein, 2005; Lipsey & Wilson, 2001).

p.947 All effect sizes were coded such that a positive effect size reflected lower anxiety and depression in the treatment group relative to the control group.

Weighted mean effect sizes, heterogeneity analyses, and moderator analyses were conducted using Comprehensive Meta-Analysis, Version 2.2.046 (Borenstein et al., 2005).

p.949 All analyses presented were conducted using a random effects model.

Note: the authors do not explicitly state a hypothesized direction of the effect. However, in their literature review and general text the authors evaluate other studies as CBM having a positive (i.e., reducing) effect on anxiety and depression. As such, we did not reverse the effect sizes. For the random-effects analysis we used the DerSimonian and Laird (DL) estimation method in the metafor package in R, since this method is the standard estimation method in Comprehensive Meta-Analysis.

The meta-analysis uses effect size g.

$$d = \frac{\bar{x}_1 - \bar{x}_2}{s} = \frac{\mu_1 - \mu_2}{s}, \text{ Hallion and Ruscio, 2011, p.947}$$

$$s = \sqrt{\frac{(n_1 - 1)s_1^2 + (n_2 - 1)s_2^2}{n_1 + n_2 - 2}}, \text{ Hallion and Ruscio, 2011, p.947}$$

$$J = 1 - \frac{3}{4(n-1)}, \text{ Hallion and Ruscio, 2011, p.947}$$

Note: the authors do not define n. We interpreted it as total sample size.

$$g = J \times d\text{, Hallion and Ruscio, 2011, p.947}$$

MA18: Ihle et al. (2012)

p.269 Effect sizes were calculated using Hedges' g (i.e., the difference in mean cognitive performance scores between the APOE e4 and the non-e4 groups divided by the pooled standard deviation), which was then transformed to the unbiased estimate Hedges' d, because the former measure overestimates effect sizes, particularly in small samples (DeCoster, 2004; Rustenbach, 2003).

If these data were not reported, Hedges' g was computed from either t statistics, F statistics with one degree of freedom in the numerator, chi-square statistics, or dichotomous dependent variables (cf. DeCoster, 2004).

Overall cognitive performance analysis: Here, individual effect sizes (Hedges' d) were pooled to derive the weighted average effect size d• across all studies as an estimation of the APOE e4-related population effect size (Hedges & Olkin, 1985; Rustenbach, 2003).

Thus, for studies that reported multiple measures to assess cognitive performance for the same samples, all of the dependent effect size estimates, Hedges' g, were averaged and transformed into Hedges' d to derive a single effect size for each study.

We argue that the aforementioned features may be important and not reflect simply noise error as it would be treated in fixed effects modeling. For this reasons, random effects models were used in the present study.

p.270 Positive values of d indicate better performance of APOE e4 carriers, whereas negative values indicate better performance of non-e4 carriers.

Note: we found effects for which no transformation formulas were available- in those cases, other standard formulas from Cooper and Hedges (1994) were used to reproduce the effect sizes. The authors use a different formulation of effect sizes: they first calculated Hedges' g (which we name Cohen's d), then correct for small sample bias and end up with an estimate of Hedges' d (which we name Hedges' g). The authors do not explicitly state a hypothesized direction of the effect. However, in their literature review the authors indicate that the support of a null or positive effect is considerably larger than a negative effect. As such, we did not reverse the effect sizes. We used the Hedges estimator in the metafor package in R, which is referenced in Hedges and Olkin (1985).

The meta-analysis uses effect size g.

$$d = \frac{\bar{x}_1 - \bar{x}_2}{s} = \frac{\mu_1 - \mu_2}{s}\text{, DeCoster, 2009, f.6.5, p.21}$$

$$s = \sqrt{\frac{(n_1 - 1)s_1^2 + (n_2 - 1)s_2^2}{n_1 + n_2 - 2}}\text{, DeCoster, 2009, f.6.6, p.21}$$

$$J = 1 - \frac{3}{4m-1}, \text{ where m = ne + nt - 2, DeCoster, 2009, f.6.7, p.21}$$

$$g = J \times d, \text{ DeCoster, 2009, p.21}$$

$$OR = \frac{AD}{BC}, \text{ Cooper et al., 2009, p.266}$$

$$d = log(OR) \times \frac{\sqrt{3}}{\pi}, \text{ Cooper et al., 2009, p.232}$$

MA19: Koenig et al. (2011)

p.624 Effect sizes were calculated with a hand calculator or DSTAT software and then entered into Comprehensive Meta-Analysis (Version 2.2.050) and Statistical Package for the Social Sciences (SPSS).

In the agency–communion paradigm, researchers reported means and standard deviations separately on the agentic and communal scales, allowing the computation of a d effect size comparing the ratings on the two scales: (M1 - M2)/sp. The effect sizes were converted to g with the correction for small sample bias: 1- [3/(4N × 9)] (Borenstein et al., 2009). Some authors split their sample at the median on both scales and reported the frequencies or percentages in each quadrant of the resulting 2x2 table. If only this report was available, agency and communion were treated as dichotomous, and g was estimated from dCox, which is a logistic transformation of the odds-ratio (Sanchez-Meca, Marın-Martınez, & Chancon-Moscoso, 2003, Formula 18).

p.630 The within-study weighting term was the conventional inverse variance for standardized comparisons of means (Lipsey & Wilson, 2001, p. 72) or dCox (Sanchez-Meca, Marin-Martinez, and Chacon-Moscoso, 2003, Formula 19), with the random-effects models also incorporating the between-studies variances in the study weight.

p.635 Consistent with the rarity of women in top positions, higher status leadership positions were expected to have a more masculine stereotype.

Note: we found effects for which no transformation formulas were available- in those cases, other standard formulas from Cooper and Hedges (1994) were used to reproduce the effect sizes. We used the DerSimonian and Laird estimator in the metafor package in R, which coincides with what is referenced in Lipsey and Wilson (2001).

The meta-analysis uses effect size g.

$$d = \frac{\bar{x}_1 - \bar{x}_2}{s} = \frac{\mu_1 - \mu_2}{s}, \text{ Borenstein et al., 2009, f.4.18}$$

$$s = \sqrt{\frac{(n_1-1)s_1^2 + (n_2-1)s_2^2}{n_1+n_2-2}}, \text{ Borenstein et al., 2009, f.4.19}$$

$$J = 1 - \frac{3}{4df - 1}$$

where *df* for two independent groups is n1+n2-2, Borenstein et al., 2009, f.4.22

$$g = J \times d,$$ Borenstein et al., 2009, f.4.23

$$d = t\sqrt{\frac{n_t + n_c}{n_t n_c}},$$ Cooper et al., 2009, p.228

$$OR = \frac{AD}{BC},$$ Borenstein et al., 2009, f.5.8

$$d = \frac{log(OR)}{1.65},$$ Sanchez-Meca et al., 2003, f.18, p.23

MA20: Kolden et al. (2011)

p.68 The effect size (ES) we used was r, the correlation coefficient for the relation between congruence and outcome. Each study was reviewed and coded by two raters (coauthors Wang and Austin). Discrepancies in original coding were negotiated in a consensus discussion involving the first author. If r was not available or nonsignificant (and not reported), we adopted the strategy of entering zero as the effect size (Lipsey & Wilson, 2001). For studies reporting multiple correlations and using multiple measures, we aggregated within each study by accounting for the dependencies of measures. The within study aggregation used the correlation matrix among measures if reported. Otherwise, we assumed that the correlation among measures was .50 when the same method was used (e.g., self-report congruence and self-report outcome) and a correlation of .25 when different methods were used (e.g., self-report vs. observation; Gleser & Olkin, 1994). The overall correlation was estimated by aggregating the correlation of each study using a weighted average where the weights were the inverse of variance of the estimates of the study level correlations (Hedges & Olkin, 1985).

We adopted a random effects model for determining overall effect size (ES) since the studies we identified were quite heterogeneous (Q=35.32, p < .01), thus violating the assumptions required for fixed effects ES modeling (e.g., homogeneity of sample, variation in study ES due only to sampling error; Hedges & Vevea, 1998).

Note: the Gleser & Olkin method for aggregation is used in the MAd package in R, and refers to standardized mean differences. We transformed the effects we wanted to aggregate to cohen's d first for aggregation, and backtransformed the aggregated effect size to r. The authors do not explicitly state a hypothesized direction of the effect, but since positive regard for the patient (congruence/genuineness) is generally seen as positive, it makes sense that the authors expect a positive relationship between congruence and improvement. We did not

reverse the effect sizes. We used the Hedges estimator in the metafor package in R, which is referenced in Hedges and Olkin (1985).

The meta-analysis uses effect size r.

$$d = X\delta + \epsilon, \text{ Gleser \& Olkin (1994), f.3.5}$$

MA21: Lucassen et al. (2011)

p.988 The Comprehensive Meta-Analysis (CMA) program was used to transform the results of the individual studies into the common metric of Pearson's product-moment correlation coefficients (r) and to combine weighted effect sizes (Borenstein et al., 2009).

Significance tests were performed through random effects models (Borenstein et al., 2009).

p.987 We expect that higher levels of paternal sensitive warmth, and in particular when co-occurring with sensitive stimulation, are associated with more infant–father attachment security.

Note: For the random-effects analysis we used the DerSimonian and Laird (DL) estimation method in the metafor package in R, since this method is the standard estimation method in Comprehensive Meta-Analysis.

The meta-analysis uses effect size r.

$$r_{y1} = \beta_1 + r_{12}(r_{y2} - \beta_1 r_{12}), \text{ Peterson and Brown, 2005, p.177}$$

MA22: Mol & Bus (2011)

p.273 All correlations between a print exposure checklist and any outcome variable were inserted into the computer program Comprehensive Meta-Analysis (Borenstein, Hedges, Higgins, & Rothstein, 2005) and transformed into Fisher's z effect sizes for further analyses, because the variance of z is approximately constant, whereas the variance of the correlation follows an asymmetrical distribution (Borenstein, Hedges, Higgins, & Rothstein, 2009). To ease interpretation of the Results section, Fisher's z summary estimates were transformed back into a correlation with the formula $r = \tanh(z)$ (Lipsey & Wilson, 2001).

For studies that did not report bivariate Pearson rs, we converted the provided statistics into Fisher's z values. A p value of .10 was entered and converted into a weighted correlation for studies that only reported that an association was not significant. Kalia (2007), however, reported the range of nonsignificant correlations, so we entered $p = .50$ for all nonsignificant values to estimate a conservative correlation in the lower end of that range. Studies in which partial correlations (k=11), converted F and t tests (k=4), or means and standard deviations (k=8) were provided were scattered through all outcome measures and did not influence the results when we analyzed the data without them.

When a study used multiple tests to measure one outcome domain, we averaged the effect sizes within that study to ensure that each study contributed only one effect size to the analysis of that domain so that each had an equal impact on the summary estimate of each domain.

For oral language, reading comprehension, and spelling skills, our stepwise approach included (a) aggregating effects of standardized and unstandardized tests into two separate composites and (b) if both were available, combining the standardized and unstandardized composites to create an overall composite per study.

p.272 Hypothesis 1: At all educational levels, indicators of the comprehension component (oral language, reading comprehension, or general achievement measures) as well as indicators of technical reading and spelling skills (basic reading skills, word recognition, or spelling) will be associated with print exposure.

Hypothesis 2: For unconstrained skills such as oral language and reading comprehension, correlations with print exposure are expected to become stronger with increasing grade levels, because readers who have pleasurable reading experiences choose to read more often.

p.276 To estimate the mean effect size, we applied the conservative random-effects model in which studies are weighted by the inverse of their variance, and, in addition, within-study error and between-study variation in true effects are accounted for (Borenstein et al., 2009).

Note: For the random-effects analysis we used the DerSimonian and Laird (DL) estimation method in the metafor package in R, since this method is the standard estimation method in Comprehensive Meta-Analysis. The authors state they transformed the Fisher's z estimated back to a correlation, but the estimate we want to reproduce is z = .27. As such, we decided not to transform back the estimates.

The meta-analysis uses effect size z.

$$z = 0.5 \times \log(\frac{1+r}{1-r}), \text{ Borenstein et al., 2009, f.6.2}$$

MA23: Morgan et al. (2011)

p.41 Most studies (k=23) used some type of pre-post design, some of which utilized a control group and some of which did not. For studies that did not utilize a control group, effect size (ES) was calculated as the standardized mean gain score (Becker, 1988; see Lipsey & Wilson, 2001), which is interpretable as a standardized mean difference similar to Cohen's d, where values around 0.2 are considered "small," 0.5 are "medium," and 0.8 or above are "large" (Cohen, 1988, pp. 25–26). All ESs were coded so that a positive value indicated improvement due to treatment. For prepost studies that included a control group, ESs were calculated using the mean gain score from the treatment group

only, so that these ESs could be directly comparable to those from studies without a control group.

If a given study had multiple ESs for a general outcome (e.g., an ES for depression and an ES for obsessive compulsive disorder under the general outcome “mental health”), then these ESs were averaged to create an overall ES for that study. However, if there was a separate ES for a subset of participants, then only the ES for the complete group of participants was used (e.g., Lovell, Allen, Johnson, & Jemelka, 2001 psychotic sample; Nelson et al., 2001).

For each outcome, we calculated a weighted mean ES, where each weight is the inverse of the estimated variance of the ES (see Lipsey & Wilson, 2001, pp. 113–114). Each ES variance was calculated as the sum of the variance due to sampling error and a random-effects variance component.

Because of the widely varying methodologies employed by the studies reviewed in this article, a random-effects analysis is clearly appropriate. In addition, we calculated a 95% confidence interval estimate of the mean effect for each outcome (see Lipsey & Wilson, 2001, pp. 113–114). These analyses were conducted using a computer macro by Wilson (2005), which utilizes the method-of-moments approach to estimating the random-effects variance component (Raudenbush, 1994).

p.39 Given findings from treatments with non-mentally disordered offenders and psychosocial rehabilitation services for PMI, it was hypothesized that services would be effective for the domain treated (i.e., correctional rehabilitation oriented services would be effective for reducing criminalness), whereas psychosocial rehabilitation oriented services would be effective at reducing symptoms of mental illness.

Note: We used the DerSimonian and Laird estimator in the metafor package in R, since this is a method of moments based approach and it uses the inverse of the variance of the effect size as weights, which coincides with the Lipsey and Wilson (2001) reference.

The meta-analysis uses effect size d.

$$d = \frac{\bar{x}_{2post} - \bar{x}_{2pre}}{\frac{S_g}{\sqrt{2(1-r)}}}, \text{ Lipsey and Wilson, 2000, p.44}$$

where Sg is the standard deviation of the gain scores, and r is the correlation between pre- and posttreatment scores. Lipsey and Wilson, 2000, p.44.

$$S_g = \sqrt{\sigma_1^2 + \sigma_2^2 - 2r\sigma_1\sigma_2}, \text{ Borenstein et al., 2009, f.4.15}$$

$$OR = \frac{AD}{BC}, \text{ Cooper et al., 2009, p.266}$$

$$d = log(OR) \times \frac{\sqrt{3}}{\pi}, \text{ Cooper et al., 2009, p.232}$$

MA24: Munder et al. (2012)

p.633 The relative effect size d with small sample correction and the corresponding standard error were calculated for each TFT comparison (Lipsey & Wilson, 2001). One effect size was calculated per treatment comparison. PTSD symptoms were defined as the outcome of interest. If more than one measure was used to assess PTSD symptoms, we selected one measure using a prespecified hierarchy of measures.

We assumed that relative effects were drawn from a population of effects, and therefore a standard inverse-variance-weighted random effect meta-analyses using a method of moments estimator (DerSimonian & Laird, 1986) was conducted within Stata 11.1 (StataCorp, College Station, TX).

p.632 In this study, true efficacy differences were eliminated by focusing on a set of treatments that are equally effective. Trauma-focused therapies (TFT) for posttraumatic stress disorder (PTSD) are such a set in that there appears to be consensus that such treatments have been rigorously shown to be equally effective (Bisson & Andrew, 2008; Bradley, Greene, Russ, Dutra, & Westen, 2005; Ehlers et al., 2010; van Etten & Taylor, 1998).

Note: since the authors assume the two TFT treatments are equally effective, we did not reverse the effect sizes. We used the DerSimonian and Laird estimator in the metafor package in R.

The meta-analysis uses effect size g.

$$d = \frac{\bar{x}_1 - \bar{x}_2}{s} = \frac{\mu_1 - \mu_2}{s}, \text{ Lipsey and Wilson, 2000, p.48}$$

$$s = \sqrt{\frac{(n_1 - 1)s_1^2 + (n_2 - 1)s_2^2}{n_1 + n_2 - 2}}, \text{ Lipsey and Wilson, 2000, p.47}$$

$$J = 1 - \frac{3}{4N - 9}, \text{ Lipsey and Wilson, 2000, p.49}$$

$$g = J \times d, \text{ Lipsey and Wilson, 2000, p.49}$$

MA25: Piet et al. (2012)

p.1009 Computed ES statistics were standardized weighted mean differences based on Hedges's g for continuous measures of anxiety, depression, and mindfulness. ESs were weighted by the inverse standard error (i.e., taking the precision of each study into account) and presented with 95% confidence intervals (CIs). Hedges's g is a variation of Cohen's d (Cohen, 1988), correcting for potential bias due to small sample sizes (Hedges & Olkin, 1985).

ESs derived from RCTs were based on mean pre- to posttreatment change scores (using the standard deviation of posttreatment scores) for both MBT and control conditions.

Our objective was by means of meta-analysis of the currently available results to test the hypothesis that MBT is an effective treatment for reduction of symptoms of anxiety and depression in adult cancer patients and survivors.

p.1010 To obtain a summary statistic, ESs were pooled across studies using the inverse variance random-effects model (DerSimonian & Laird, 1986).

Note: we used the DerSimonian and Laird estimator in the metafor package in R.

The meta-analysis uses effect size g.

$$d = \frac{\Delta_1 - \Delta_2}{s}$$

where Δ_1 1 and Δ_2 are the mean pre–post change scores for the treatment group and control condition, respectively. Piet, Würtzen, and Zachariae, 2012, p.1010, footnote 2.

$$s = \sqrt{\frac{(n_1 - 1)s_1^2 + (n_2 - 1)s_2^2}{n_1 + n_2 - 2}},$$ Piet et al., 2012, p.1010, footnote 2.

s_1 and s_2 are the standard deviations of the posttreatment scores for each group.

$$J = 1 - \frac{3}{4df - 1}$$

where df for two independent groups is n1+n2-2, Piet et al., 2012, p.1010, footnote 2.

$g = J \times d$, Piet et al., 2012, p.1010, footnote 2.

MA26: Smith & Silva (2011)

p.45 The studies included in this meta-analysis frequently (90%) reported data in terms of bivariate correlations (Pearson's r). Reports including other statistics (e.g., analyses of variance, t tests, p values) were transformed to the metric of r with statistical software. Coders assigned a positive value to effect sizes indicating a stronger ethnic identity co-occurring with greater well-being (or weaker ethnic identity co-occurring with, e.g., symptoms of mental il ess, distress), with a negative value indicating an inverse association between ethnic identity and personal well-being. In two cases when an analysis was reported to be statistically significant but no statistic was provided, the r value was determined by the corresponding alpha level (assuming two-tailed $\alpha = .05$ unless reported otherwise). In six cases, analyses described as nonsignificant without any additional information were set to $r = .00$.

To overcome this issue, we averaged all effect sizes within each study (weighted by the number of participants included in each analysis) to compute an aggregate effect size for that particular study (Mullen, 1989). Thus, each study contributed only one data point to the calculation of the omnibus effect size. However, in one instance where a grouping variable that was found to moderate

the omnibus results required subsequent detailed exploration for better interpretation of the finding (the type of dependent measure used within studies), we conducted an additional analysis by shifting the unit of analysis (Cooper, 1998). In that analysis, we included multiple effect sizes within studies if they were based on distinct measures of wellbeing (i.e., self-esteem and symptoms of depression). Thus, this approach disaggregated results across conceptually distinct measures used within studies.

Because factors other than ethnic identity influence well-being and because the magnitude of the association between ethnic identity and well-being was expected to differ across individual participants and across individual studies, random effects models were used in analyzing the data with macros for SPSS provided by Lipsey and Wilson (2001).

Note: since the authors do not use any references to formulas used to transform effect sizes, we used standard formulas from Cooper and Hedges (1994). The authors do not explicitly state a hypothesized direction of the effect, but it is clear from the text that they expect to find a positive correlation (e.g., "Scholars have consistently concluded that a strong ethnic identity is positively associated with personal well-being and successful life adjustment for people of color (p.44)". We did not recode the effect sizes. The SPSS macro from Lipsey and Wilson (2001) estimates inverse variance weights and uses, among others, a noniterative method of moments estimator. This coincides with the DerSimonian and Laird estimator in the metafor package in R, which we used for estmation.

The meta-analysis uses effect size r.

$$d = \frac{\bar{x}_1 - \bar{x}_2}{s} = \frac{\mu_1 - \mu_2}{s}, \text{ Cooper et al., 2009, p.226}$$

$$s = \sqrt{\frac{(n_1 - 1)s_1^2 + (n_2 - 1)s_2^2}{n_1 + n_2 - 2}}, \text{ Cooper et al., 2009, p.226}$$

$$r = \frac{d}{\sqrt{d^2 + a}}, \text{ Cooper et al., 2009, p.234}$$

$$a = \frac{(n_1 + n_2)^2}{n_1 n_2}, \text{ Cooper et al., 2009, p.234}$$

MA27: Tillman (2011)

p.1015 Pearson correlation coefficients for the relation between simple and complex span tasks derived from each study, and the mean age of each sample, were used as the relevant primary data in the present meta-analysis.

Weighted regression analysis with method of moments random effects, adapted for meta-analytic purposes by Lipsey and Wilson (2001), was used in the meta-analysis. The meta-analytic computations were done with SPSS, using syntaxes

provided by Lipsey and Wilson. To study the effects of age on the relation between simple and complex span tasks, the simple span–complex span correlation coefficients were used as outcome, mean age of sample as predictor, and the inverse variance (calculated as n − 3; see Lipsey & Wilson, 2001) as weight variable.

About half of the included samples ($n = 27$) reported more than one correlation coefficient of the simple span–complex span relation (because they used several simple and/or complex span tasks; see note in Table 1). As recommended by Hunter et al. (1986), the correlation coefficients in these cases were averaged to yield a single effect size estimate for that sample.

Note: no specific formulas for this meta-analysis, since only single correlations or the mean of multiple correlations were extracted or calculated. The author does not explicitly state a hypothesized direction of the effect we are researching, but it is clear from the text that they expect to find a positive correlation (e.g., "the developmental hypothesis concerning the relation between simple and complex spans is that these immediate memory tasks are more strongly related in children than in adults. (p.1013)". We did not recode the effect sizes. The SPSS macro from Lipsey and Wilson (2001) estimates inverse variance weights and uses, among others, a noniterative method of moments estimator. This coincides with the DerSimonian and Laird estimator in the metafor package in R, which we used for estmation.

The meta-analysis uses effect size r.

MA28: Toosi et al. (2012)

p.11 We calculated effect sizes using the r statistic, as recommended by Rosenthal, 1991, through one of several methods. If the study reported an F value with one degree of freedom in the numerator, a t value, a chi-square value with one degree of freedom, or a Z value that directly compared the outcomes for the same-race and interracial dyads, we were able to calculate the r value using formulas provided in Rosenthal, 1991. Studies that reported a beta value were included as well, using the approximation suggested by Peterson and Brown (2005). Alternatively, if studies did not directly provide these statistics we often were able to obtain the means, standard deviations, and sample sizes so that a two-sample t test comparing the same-race and interracial dyads could be calculated (Rosenthal & Rosnow, 1991). When multiple values for the same category of dependent measures could be calculated from a single participant sample, the effect sizes were averaged into a single value, using an unweighted Fisher's Z-to-r transformation.

When results indicated the presence of bias in favor of same race partners over other-race partners, the effect sizes were considered congruent with expectations of prejudice in interracial interactions and were assigned a positive sign. When results showed a bias in favor of other-race partners over same-race partners, the effect sizes were assigned a negative sign. When authors reported

no significant differences between interracial and same-race dyads and sufficient data could not be obtained to calculate an exact value or direction, those effect sizes (k=19) were set equal to zero.

Random-effects models were used to calculate the overall effect sizes.

For random-effects models, study effect sizes are weighted by the inverse of the variance and combined, incorporating an estimate of between-study variance. To calculate mean effect sizes, we used the Comprehensive Meta-Analysis software package (Version 2; Borenstein, Hedges, Higgins, & Rothstein, 2005).

Note: we found effects for which no transformation formulas were available-in those cases, other standard formulas from Cooper and Hedges (1994) were used to reproduce the effect sizes. When we found multiple values for the same category of dependent measures, we averaged the effect sizes, since it is unclear what the authors meant with an "unweighted Fisher's Z-to-r transformation", given the fact that we do not calculate Fisher's z estimates. For the random-effects analysis we used the DerSimonian and Laird (DL) estimation method in the metafor package in R, since this method is the standard estimation method in Comprehensive Meta-Analysis.

The meta-analysis uses effect size r.

$$d = \frac{\bar{x}_1 - \bar{x}_2}{s} = \frac{\mu_1 - \mu_2}{s}, \text{ Rosenthal, 1991, f.2.4}$$

$$s = \sqrt{\frac{(n_1 - 1)s_1^2 + (n_2 - 1)s_2^2}{n_1 + n_2 - 2}}, \text{ Cooper et al., 2009, p.226}$$

$$r = \sqrt{\frac{t^2}{t^2 + (n_1 + n_2 - 2)}}, \text{ Rosenthal, 1991, f.2.16}$$

$$r = \sqrt{\frac{\sqrt{F}}{\sqrt{F} + (n_1 + n_2 - 2)}}, \text{ Rosenthal, 1991, p.15:}$$

If the test statistic employed was F (from analysis of variance) and df for the numerator was unity, we take the as t and proceed as we did in the case of t with df equal to the df of the denominator of the F ratio

$$r = \frac{d}{\sqrt{d^2 + a}}, \text{ Rosenthal, 1991, f.2.19 \& 2.20}$$

$$a = \frac{(n_1 + n_2)^2}{n_1 n_2}, \text{ Rosenthal, 1991, f.2.19 \& 2.20}$$

MA29: van Iddekinge et al. (2011)

p.1173 We implemented Hunter and Schmidt's (2004) psychometric approach to meta-analysis. We began by identifying (and/or computing) the observed

validity coefficient(s) within each primary study. Most studies reported zero-order correlations. When other statistics were reported (e.g., t, M, and SD), we converted them to correlations using formulae provided by Hunter and Schmidt.

For studies that reported a single validity coefficient between one interest scale and one criterion measure, we used that coefficient in our analyses. For studies that reported validities for multiple interest scales (e.g., scales for each RIASEC dimension), we recorded the interest scale that was theoretically most relevant to the target job or vocation. For most studies, it was clear which scale was most relevant.

p.1169 To the extent training reflects the knowledge and skill requirements of the job, employees whose interests are congruent with the job may be more motivated to perform during training (and, in turn, acquire job-relevant knowledge and skills) than people whose interests are not as congruent with the job.

Note we used: no specific formulas for this meta-analysis, since only single correlations or the mean of multiple correlations were extracted or calculated. The authors do not indicate whether they used a fixed effect or random-effects model. We estimated both. For the random-effects meta-analysis, we used the Hunter & Schmidt estimation method in the metafor package in R.

The meta-analysis uses effect size r.

MA30: Webb et al. (2012)

p.784 Two types of comparisons were deemed to provide useful information about the effects of different ER strategies: (a) a comparison between participants who were given regulation instructions (experimental condition) and participants who were given alternative instructions (either another experimental condition or a control condition) or (b) a within-participants comparison between trials where participants were given regulation instructions and trials where participants were given alternative instructions. Where experiments included manipulations of more than one ER strategy or more than one control condition, we included all relevant comparisons. Where possible, we compared each ER strategy with each control strategy. However, if no control strategy was included, we compared the experimental strategies with each other.

Whenever multiple comparisons from one experiment led to the same participants being represented in more than one effect size, we adjusted the N for each group accordingly when calculating the standard error (i.e., if control instructions were compared to both reappraisal instructions and suppression instructions, we computed effect sizes for both comparisons but halved the N for the control group when calculating the standard error.

For example, if the effect was nonsignificant we assumed zero difference (d=0.00). If the effect was significant at p < .05 we used the smallest value of d (given the sample size) that was significant at this level of alpha (Lipsey & Wilson, 2001).

Some ER instructions aimed at improving affect, whereas others were in-

tended to worsen affect. Thus, we did not code our effect sizes in terms of hedonic success (i.e., a decrease in negative affect or increase in positive affect). Instead, we calculated effect sizes in terms of regulation success according to the strategy's aims. For example, if a strategy was intended to reduce anger, the data were coded such that a positive effect size represented a reduction in the experiential, physiological, or behavioral components of anger. If a strategy was intended to increase anger, the data were coded such that a positive effect size represented an increase in these components of anger.

p.785 Where outcomes were measured at multiple timepoints, we used data from the ER period (e.g., physiological measures of experience during regulation) or the nearest timepoint after the ER period (e.g., self-report measures completed after the regulation attempt). Where there were multiple time points within the ER period (e.g., Borton & Casey, 2006) or values were reported for multiple ER periods (e.g., Hunt, 1998), an average effect size was computed prior to inclusion in the main data set.

p.791 Computations were undertaken using STATA Version 11 and Comprehensive Meta-Analysis Version 2 (Borenstein, Hedges, Higgins, & Rothstein, 2005). Weighted average effect sizes ($d+$) were based on a random effects model because studies were likely to be "different from one another in ways too complex to capture by a few simple study characteristics" (Cooper, 1986, p. 526).

Note: since the authors do not use any references to formulas used to transform effect sizes, we used standard formulas from Borenstein et al., 2009 and Cooper and Hedges (1994). We used the DerSimonian and Laird (DL) estimation method in the metafor package in R, since this method is the standard estimation method in Comprehensive Meta-Analysis.

The meta-analysis uses effect size d.

$$d = \frac{\bar{x}_1 - \bar{x}_2}{s} = \frac{\mu_1 - \mu_2}{s}, \text{ Borenstein et al., 2009, f.4.18}$$

$$s = \sqrt{\frac{(n_1 - 1)s_1^2 + (n_2 - 1)s_2^2}{n_1 + n_2 - 2}}, \text{ Borenstein et al., 2009, f.4.19}$$

$$d = t\sqrt{\frac{n_t + n_c}{n_t n_c}}, \text{ Cooper et al., 2009, p.228}$$

MA31: Woodin (2011)

p.328 A separate effect size was computed for each observational code and for each analysis (i.e., gender difference or relationship satisfaction). Effect sizes were computed using Cohen's (1988) *d*, which was then corrected for small sample bias (Hedges & Olkin, 1985). Most of the data used to analyze gender differences and relationship satisfaction were reported as means and standard deviations. Cohen's *d* was also computed in several cases based on the reported test statistic (*F* ratio or *t* ratio) and was also at times transformed

from a correlation coefficient (r). In several instances, means and standard deviations were reported separately for different groups (e.g., male and female behavior reported separately for high- vs. low-relationship satisfaction) or different conversations (e.g., male topic vs. female topic). In this case, means and standard deviations were weighted for sample size and averaged across groups. When differences were reported as nonsignificant, but inadequate information was provided to calculate an effect size, the conservative approach of assigning a d of zero was used (Lipsey & Wilson, 2001). Finally, several effect sizes were significant outliers (greater than 2 *SD*s from the mean) compared to others in the same category. In each case, the effect size was Windsorized by recoding each outlier to be slightly larger than the next largest effect size in the category, so that one large effect size did not skew the overall findings (Lipsey & Wilson, 2001).

A conservative decision rule was created to deal with combination codes (e.g., more than one code combined into a summary code), such that coders were instructed to choose the metacode that represented the highest intensity behavior represented by the combination code. For instance, a combination code that included belligerence, anger, and sadness would be coded as hostility even if it contained some distress behavior.

p.329 Analyses began with fixed-effects models, as effect sizes were theorized to vary systematically based on characteristics of individual studies (Lipsey & Wilson, 2001). Overall effect sizes with significant heterogeneity were then tested for study moderators.

p.327 On the basis of previous research and theory, the hypotheses were that (a) women would engage in the higher intensity behaviors of hostility, distress, and intimacy more than men; (b) men would engage in the more low-intensity behaviors of withdrawal and problem solving more than women; (c) higher intensity behaviors (hostility and intimacy) would be closely associated with relationship satisfaction; and (d) lower intensity behaviors (distress, withdrawal, problem solving) would be less closely related to relationship satisfaction.

Note: we found effects for which no transformation formulas were available- in those cases, other standard formulas from Cooper and Hedges (1994) were used to reproduce the effect sizes. The authors state they corrected their Cohen's d estimates for small sample bias (i.e., they transformed them to Hedges' g). However, since the final primary study effect size in the meta-analysis is a correlation, we assumed they used the Hedges' g effect size as input when transforming to correlations. The author does not specifically state whether they expect a negative or positive correlation, but since hostility is regarded as negative affect, we assume the author does expect a negative correlation between hostility and marital adjustment. We reversed the effect sizes for this meta-analysis, so positive effect sizes indicate a negative correlation between marital adjustment and hostility, as we assume is expected by the author.

The meta-analysis uses effect size g.

$$d = \frac{\bar{x}_1 - \bar{x}_2}{s} = \frac{\mu_1 - \mu_2}{s}, \text{ Hedges and Olkin, 1985, p.78}$$

$$s = \sqrt{\frac{(n_1 - 1)s_1^2 + (n_2 - 1)s_2^2}{n_1 + n_2 - 2}}, \text{ Hedges and Olkin, 1985, p.79}$$

$$J = 1 - \frac{3}{4N - 9}, \text{ Hedges and Olkin, 1985, p.81}$$

$$g = J \times d, \text{ Hedges and Olkin, 1985, p.81}$$

$$d = \frac{2r}{\sqrt{1 - r^2}}, \text{ Borenstein et al., 2009, f.7.5}$$

$$r = \sqrt{\eta^2}, \text{ Levine and Hullett, 2002, p.615}$$

MA32: Woodley (2011)

p.229 In this section the results of a meta-analysis involving all known correlations between g and K (both published and unpublished) will also be presented demonstrating the independence of these variables.

Note: the author does not indicate whether they used a fixed effect or random effects model. We estimated both. Since we do not know what program or estimation method were used, we used the standard DerSimonian-Laird estimator for the random-effects analysis in the metafor package in R. We used no specific formulas for this meta-analysis, since only single correlations were extracted. The author does not explicitly state a hypothesized direction of the effect. The author investigates the relationship between g (IQ) and K (life history speed). They expect a correlation ("There is every indication therefore that g and direct measures of life history speed should correlate at individual differences scales") but do not state the direction. One of the evolutionary theories they discuss is the life history differential K theory, which states that life history speed negatively correlates with fitness indicators (g, IQ) because the environment being developed is less stable. The remainder of the article focuses on hypotheses being related to mediators. Since we did not want to deviate from the original meta-analysis if not needed, we decided to not reverse code the effect, so a positive effect indicates a positive correlation between the two variables.

The meta-analysis uses effect size r.

MA33: Yoon et al. (2011)

p.90 The correlation coefficient (i.e.,r) was the effect size measure of choice. For each meta-analysis, only one effect size was included from each study. For example, when multiple relevant correlations were reported from the same sample

(e.g., Wong, Tran, & Lai, 2009), the average of the correlations was coded as the effect size. When the correlation was reported for each subgroup of the sample (e.g., Yeh, 2003), the correlation with the overall sample was coded as the effect size. When the correlations for both acculturation/ enculturation total scales and dimensions were reported, we chose the correlations with total scales (e.g., Obasi & Leong, 2009). Four studies reported only standardized regression weights without any information to calculate correlations (e.g., Cavazos-Rehg & DeLucia-Waack, 2009; Rahman & Rollock, 2004; Rodriguez, Mira, Morris, & Cardoza, 2003; Tsai et al., 2000). Following Hunter and Schmidt's (2004) claim that standardized regression weights could validly substitute for correlations in meta-analyses, we used standardized regression weights for the four studies (see Poropat, 2009). To avoid any problem associated with the standard error formulation of correlation coefficients, we converted each effect size of r to zr by using Fisher's r-to-z transformation to calculate the$Q$ statistic for homogeneity test and the mean effect size.

We used random effects models because they make inferences about a population of studies beyond the present sample of studies by considering both within-study and between-study variability.

Note: the author does not explicitly state a hypothesized direction of the effect. The authors investigate the relationship of acculturation/enculturation and psychological distress/depression or self-esteem. Since acculturation is, among others, defined as "cultural adaptation that occurs as a result of contact between multiple cultures", and the authors state "acculturation and enculturation showed patterns of noninverse correlations with other variables (i.e., personality, self-identity, and psychosocial adjustment" (p.84), we assume a negative correlation between acculturation and distress/depression or self-esteem is expected. We reversed the effect sizes for this meta-analysis, so positive effect sizes indicate a negative correlation between acculturation and distress/depression, as we assume is expected by the authors. Since we do not know what program or estimation method were used and the authors reference Lipsey and Wilson (2001) when discussing the random-effects analysis, we used the standard DerSimonian-Laird estimator in the metafor package in R.

The meta-analysis uses effect size r.

$$r_{y1} = \beta_1 + r_{12}(r_{y2} - \beta_1 r_{12}), \text{ Peterson and Brown, 2005, p.177}$$

$$Z_r = \frac{1}{2}\log\frac{(1+r)}{(1-r)}, \text{ Cooper et al., 2009, p.231}$$

$$r = \tanh(Z_r)$$

General formulas

$$r_{pb} = \frac{d}{\sqrt{d^2+h}}, \text{ Jacobs and Viechtbauer, 2016, f.5}$$

$$h = \frac{m}{n_1} + \frac{m}{n_0}, m = n_1 + n_0 - 2, \text{ Jacobs and Viechtbauer, 2016, f.5}$$

$$r_b = \frac{\sqrt{pq}}{f(z_p)} r_{pb}, \text{ Jacobs and Viechtbauer, 2016, f.8}$$

where *f(zp)* denotes the density of the standard normal distribution at value *zp*, which is the point for which *P(Z > zp) = p*, with *Z* denoting a random variable following a standard normal distribution.

$$p = \frac{n_1}{n}, q = 1 - p, \text{ Jacobs and Viechtbauer, 2016, f.6}$$

$$z_{rb} = \frac{a}{2} \log(\frac{1 + ar_b}{1 - ar_b}), \text{ Jacobs and Viechtbauer, 2016, f.17}$$

$$a = \frac{\sqrt{f(z_p)}}{\sqrt[4]{pq}}, \text{ Jacobs and Viechtbauer, 2016, f.17}$$

References

Borenstein, M., Hedges, L. V., Higgins, J. P. T., & Rothstein, H. R. (2009). *Introduction to Meta-Analysis.* doi:10.1002/9780470743386

Card, N. A., Bosch, L., Casper, D. M., Wiggs, C. B., Hawkins, S. A., Schlomer, G. L., & Borden, L. M. (2011). A meta-analytic review of internalizing, externalizing, and academic adjustment among children of deployed military service members. *Journal of Family Psychology*, *25*(4), 508–520. doi:10.1037/a0024395

Cohen, J. (1988). *Statistical Power Analysis for the Behavioral Sciences.* Mahwah, NJ: Taylor & Francis Inc.

Cooper, H. M., Hedges, L. V., & Valentine, J. C. (Eds.). (2009). *The Handbook of Research Synthesis and Meta-Analysis.* New York City, NY: Russell Sage Found.

DeCoster, J. (2009). *Meta-Analysis Notes.*

Hallion, L. S., & Ruscio, A. M. (2011). A meta-analysis of the effect of cognitive bias modification on anxiety and depression. *Psychological Bulletin*, *137*(6), 940–958. doi:10.1037/a0024355

Hedges, L. V., & Olkin, I. (1985). *Statistical Methods for Meta-Analysis.* Cambridge, MA: Academic Press.

Hunter, J. E., & Schmidt, F. L. (2004). *Methods of Meta-Analysis: Correcting Error and Bias in Research Findings.* New York City, NY: SAGE Publications, Inc.

Jacobs, P., & Viechtbauer, W. (2016). Estimation of the biserial correlation and its sampling variance for use in meta-analysis. *Research Synthesis Methods*, *8*(2), 161–180. doi:10.1002/jrsm.1218

Levine, T. R., & Hullett, C. R. (2002). Eta Squared, Partial Eta Squared, and Misreporting of Effect Size in Communication Research. *Human Communication Research*, *28*(4), 612–625. doi:10.1111/j.1468-2958.2002.tb00828.x

Lipsey, M. W., & Wilson, D. (2000). *Practical Meta-Analysis (Applied Social Research Methods)*. New York City, NY: SAGE Publications, Inc.

Peterson, R. A., & Brown, S. P. (2005). On the Use of Beta Coefficients in Meta-Analysis. *Journal of Applied Psychology*, *90*(1), 175–181. doi:10.1037/0021-9010.90.1.175

Piet, J., Würtzen, H., & Zachariae, R. (2012). The effect of mindfulness-based therapy on symptoms of anxiety and depression in adult cancer patients and survivors: A systematic review and meta-analysis. *Journal of Consulting and Clinical Psychology*, *80*(6), 1007–1020. doi:10.1037/a0028329

Rosenthal, R. (1991). *Meta-Analytic Procedures for Social Research*. doi:10.4135/9781412984997

Rosenthal, R., & DiMatteo, M. R. (2001). Meta-Analysis: Recent Developments in Quantitative Methods for Literature Reviews. *Annual Review of Psychology*, *52*(1), 59–82. doi:10.1146/annurev.psych.52.1.59

Sanchez-Meca, J., Marin-Martinez, F., & Chacon-Moscoso, S. (2003). Effect-Size Indices for Dichotomized Outcomes in Meta-Analysis. *Psychological Methods*, *8*(4), 448–467. doi:10.1037/1082-989x.8.4.448

Wampold, B. E., Mondin, G. W., Moody, M., Stich, F., Benson, K., & Ahn, H.-n. (1997). A meta-analysis of outcome studies comparing bona fide psychotherapies: Empiricially, "all must have prizes.". *Psychological Bulletin*, *122*(3), 203–215. doi:10.1037/0033-2909.122.3.203

Whitener, E. M. (1990). Confusion of confidence intervals and credibility intervals in meta-analysis. *Journal of Applied Psychology*, *75*(3), 315–321. doi:10.1037/0021-9010.75.3.315
