# Supplementary material for: Reproducibility of individual effect sizes in meta-analyses in psychology
Source: PLoS One. 2020 May 27;15(5):e0233107. doi: 10.1371/journal.pone.0233107 (PMC7252608; doi:10.1371/journal.pone.0233107)
Supplement: S4 File — https://osf.io/65b8z/. (PDF) [file pone.0233107.s004.pdf]

Reproducibility of Individual Effect Sizes in Meta-Analyses in Psychology
Supplement D. Reported vs Reproduced primary study effect sizes

Supplement D contains for each meta-analysis separately the primary study effect sizes as reported (the horizontal axis) and the primary study effect sizes as reproduced (the vertical axis). All data points on the diagonal line indicate Reproducible between reported and reproduced primary study effect sizes. In the plot, the original meta-analytic effect size estimate (as we computed it from the reported primary study effect sizes), as well as the reproduced meta-analytic effect size estimate are given. The k in the title refers to the number of primary study effect sizes we reproduced.

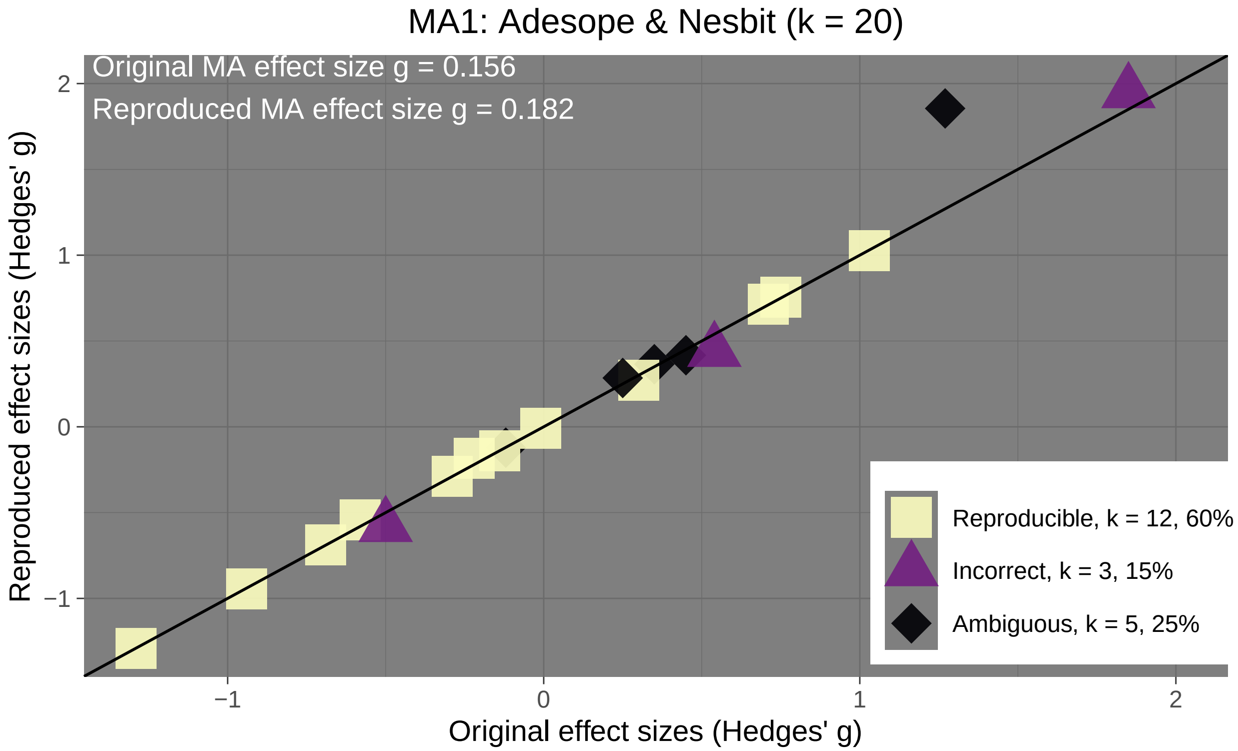

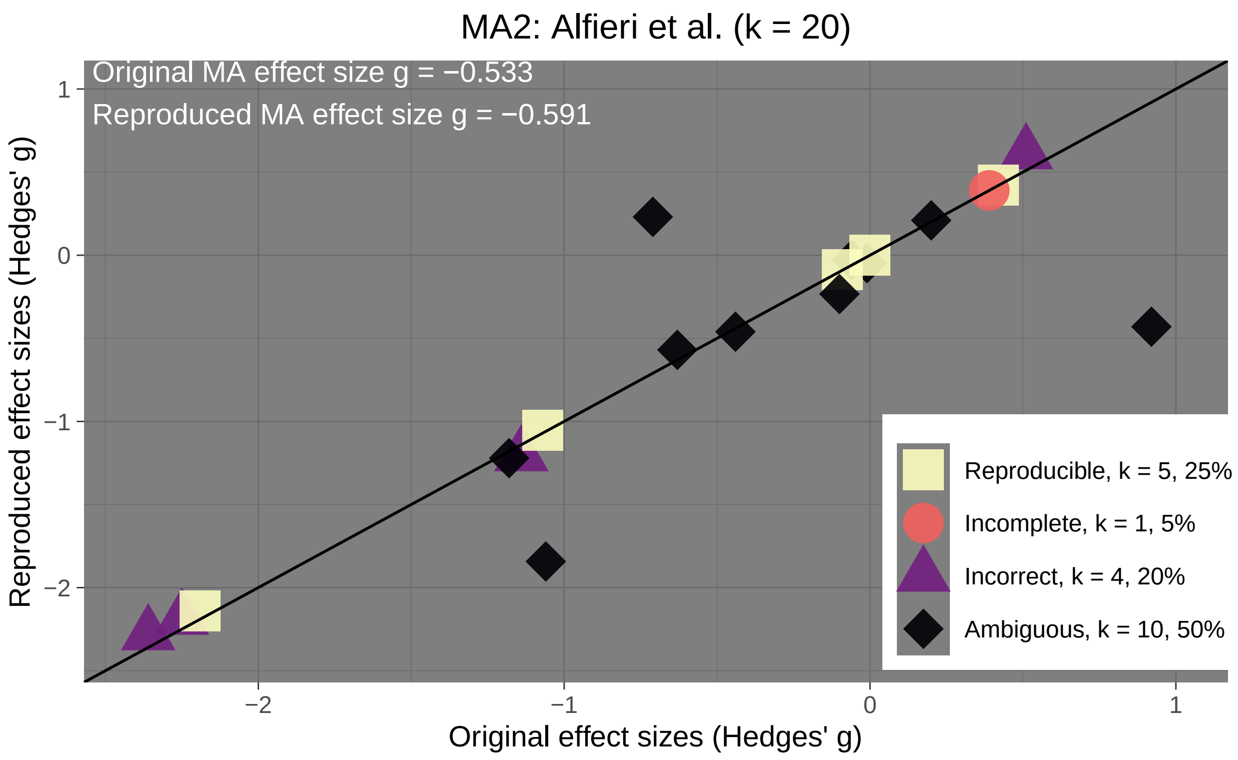
MA2: Alfieri et al. (k = 20)
Original MA effect size g = −0.533
Reproduced MA effect size g = −0.591
Reproducible, k = 5, 25%
Incomplete, k = 1, 5%
Incorrect, k = 4, 20%
Ambiguous, k = 10, 50%
Reproduced effect sizes (Hedges' g)
Original effect sizes (Hedges' g)

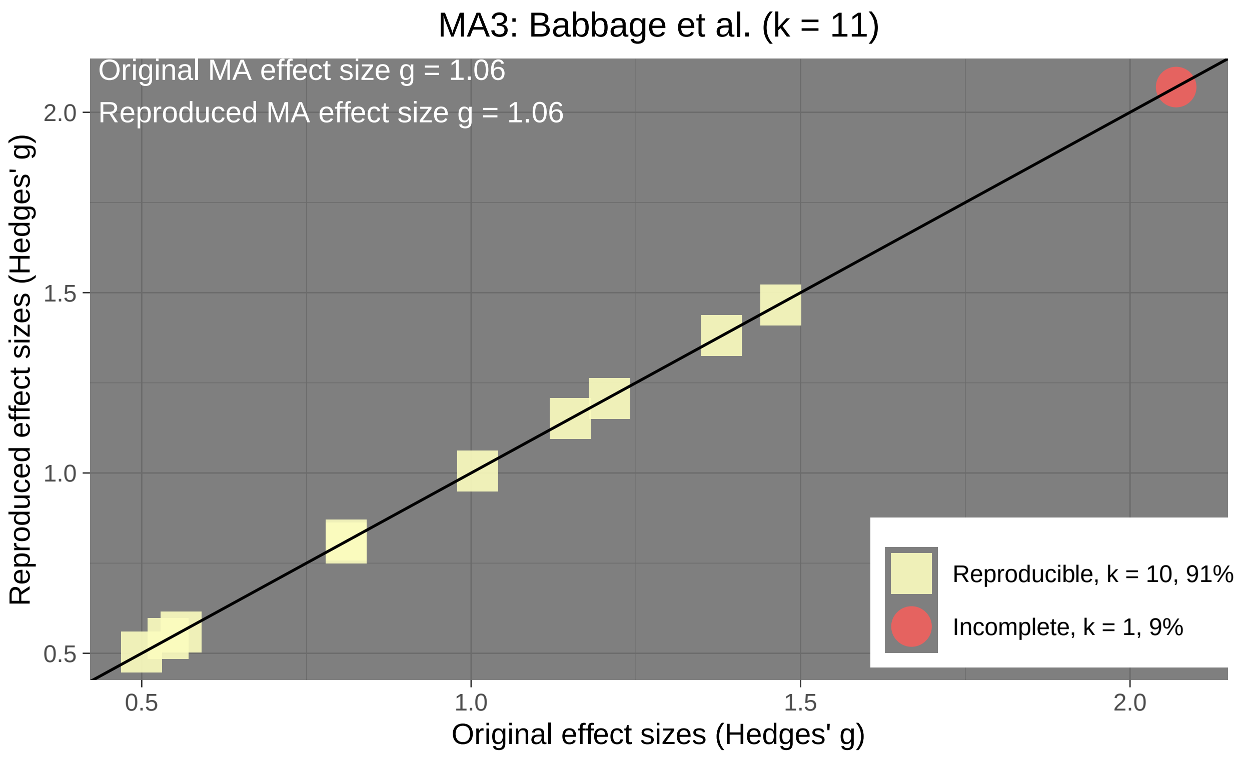
MA3: Babbage et al. (k = 11)
Original MA effect size g = 1.06
Reproduced MA effect size g = 1.06
Reproducible, k = 10, 91%
Incomplete, k = 1, 9%
Reproduced effect sizes (Hedges' g)
Original effect sizes (Hedges' g)

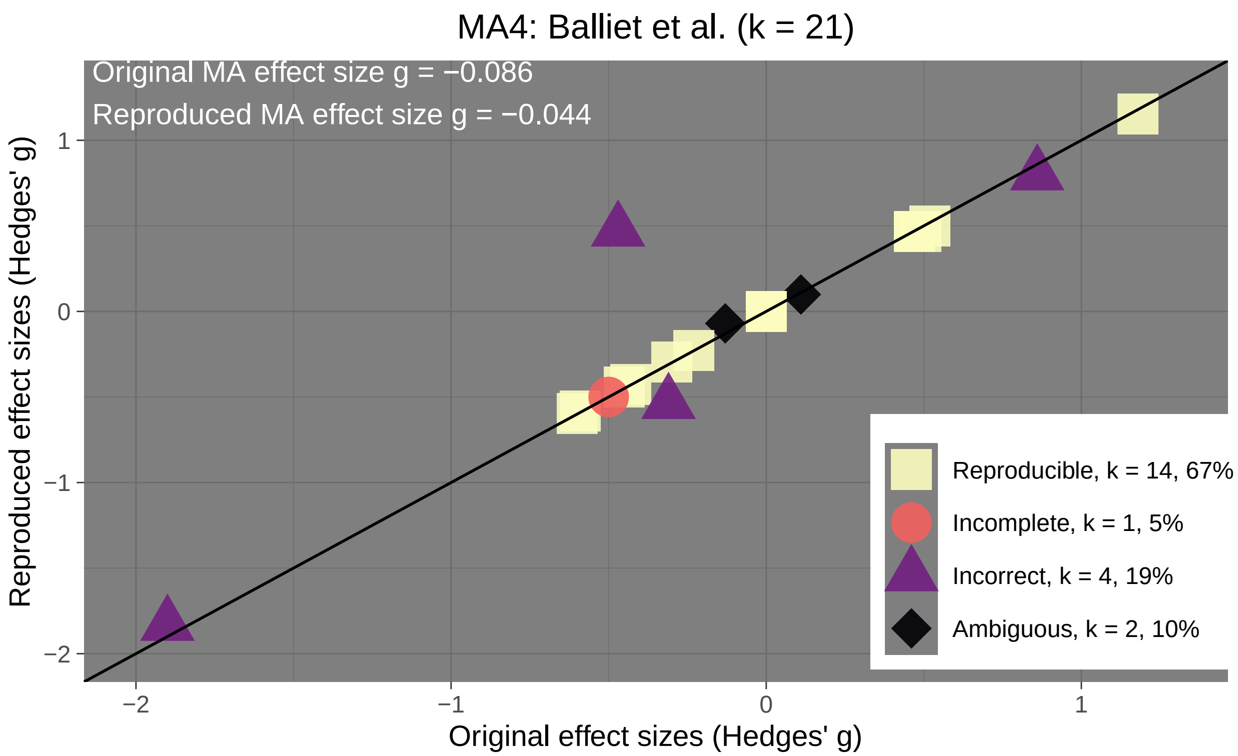
MA4: Balliet et al. (k = 21)
Original MA effect size g = −0.086
Reproduced MA effect size g = −0.044
Reproducible, k = 14, 67%
Incomplete, k = 1, 5%
Incorrect, k = 4, 19%
Ambiguous, k = 2, 10%
Reproduced effect sizes (Hedges' g)
Original effect sizes (Hedges' g)
1
0
−1
−2
−2
−1
0
1

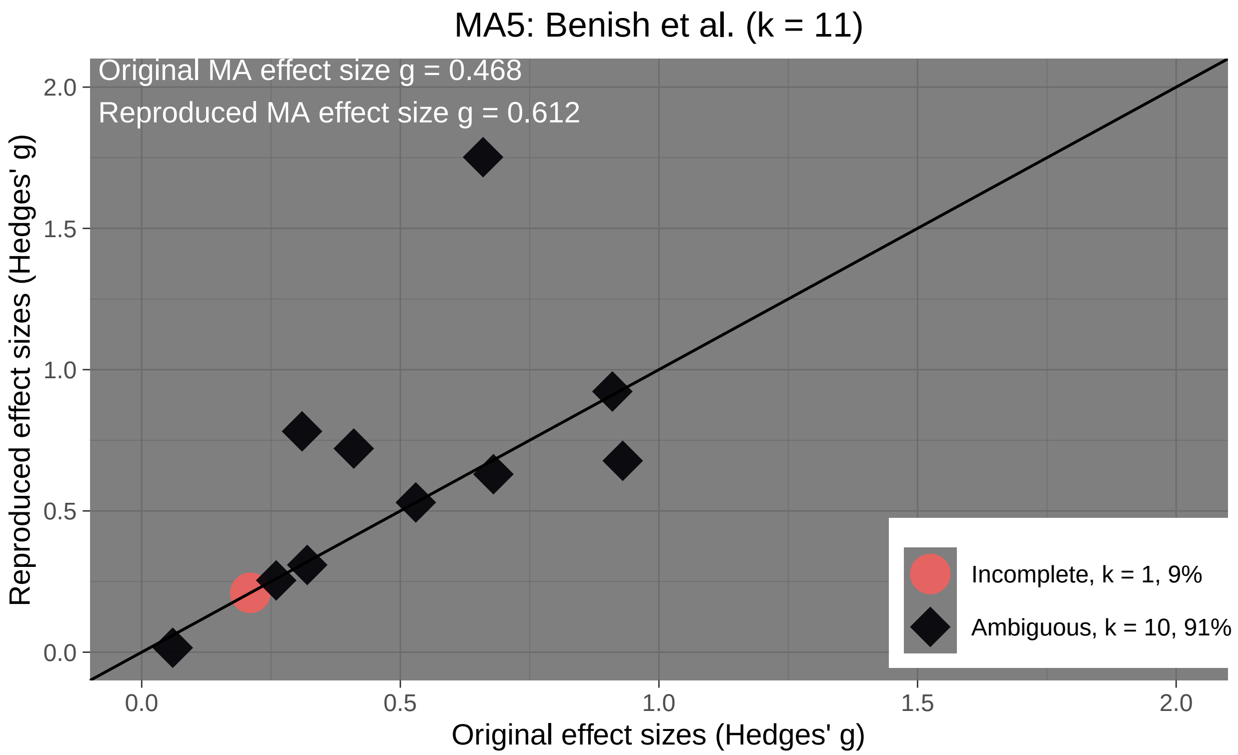
MA5: Benish et al. (k = 11)
Original MA effect size g = 0.468
Reproduced MA effect size g = 0.612
Incomplete, k = 1, 9%
Ambiguous, k = 10, 91%
Reproduced effect sizes (Hedges' g)
Original effect sizes (Hedges' g)
2.0
1.5
1.0
0.5
0.0
0.0
0.5
1.0
1.5
2.0

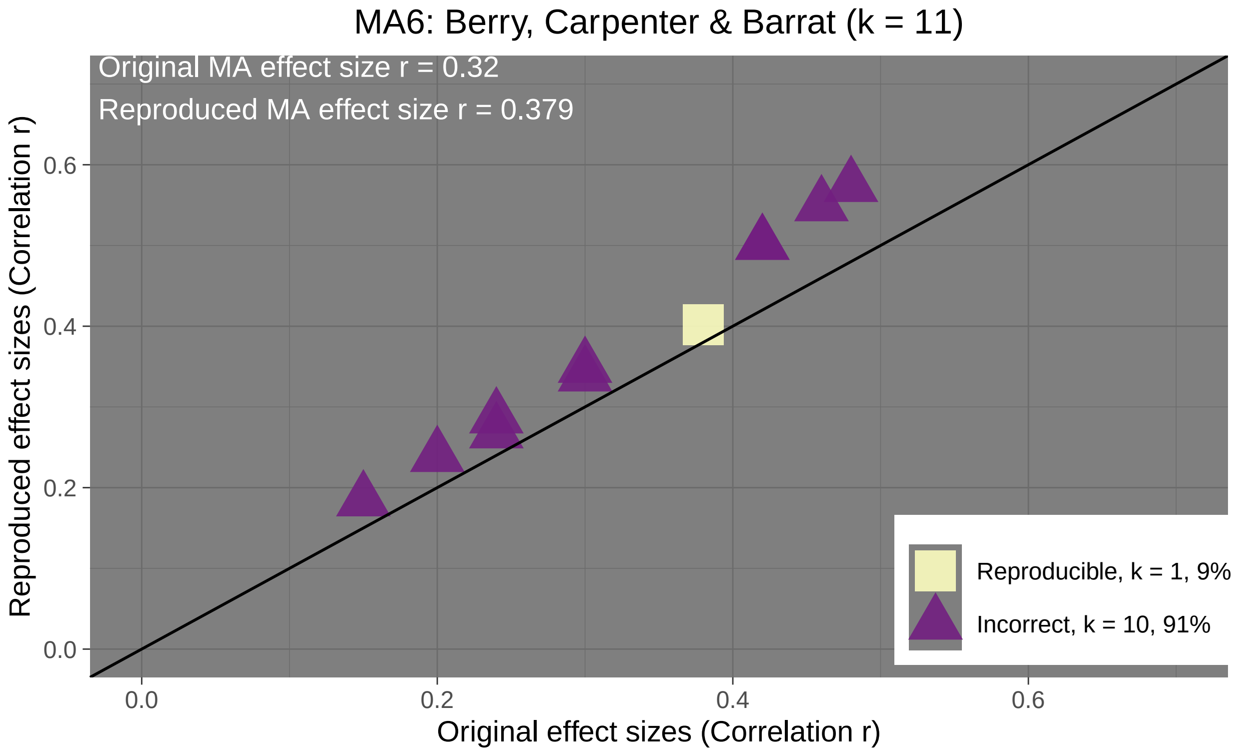
MA6: Berry, Carpenter & Barrat (k = 11)
Original MA effect size r = 0.32
Reproduced MA effect size r = 0.379
Reproduced effect sizes (Correlation r)
0.0
0.2
0.4
0.6
Original effect sizes (Correlation r)
Reproducible, k = 1, 9%
Incorrect, k = 10, 91%

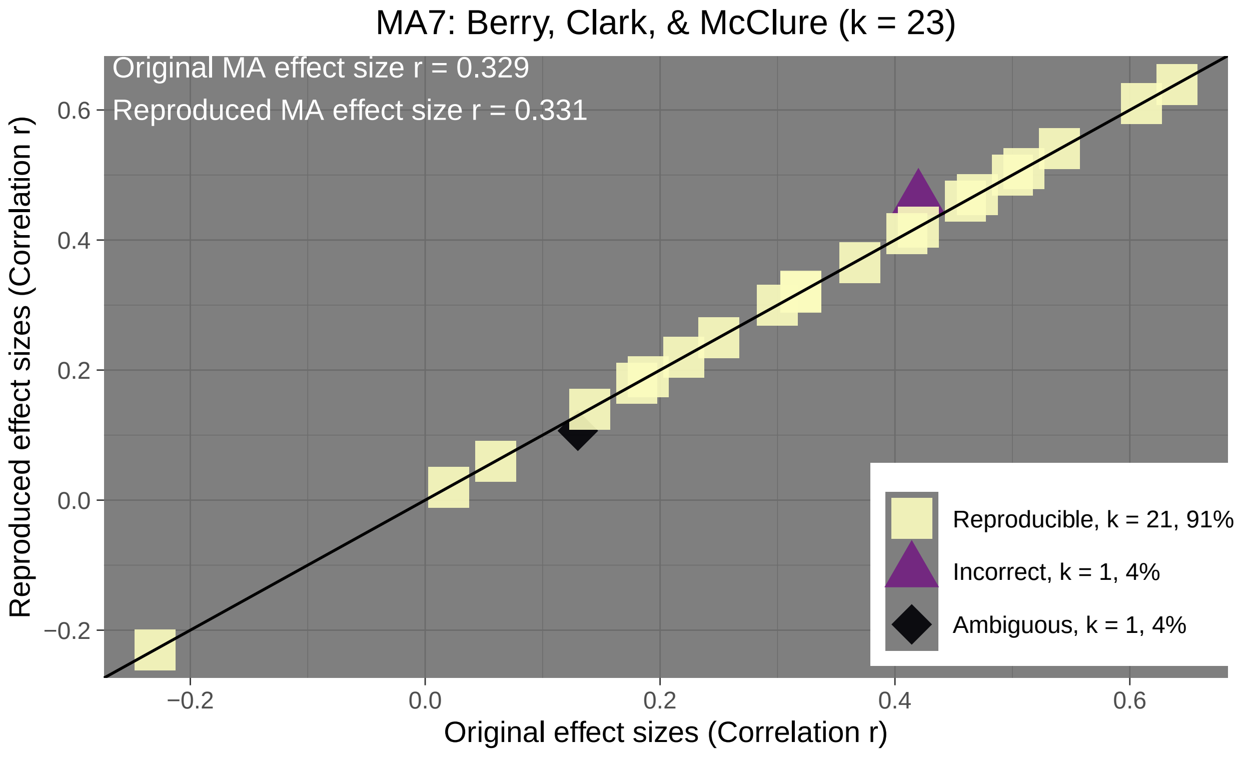
MA7: Berry, Clark, & McClure (k = 23)
Original MA effect size r = 0.329
Reproduced MA effect size r = 0.331
Reproduced effect sizes (Correlation r)
−0.2
0.0
0.2
0.4
0.6
Original effect sizes (Correlation r)
Reproducible, k = 21, 91%
Incorrect, k = 1, 4%
Ambiguous, k = 1, 4%

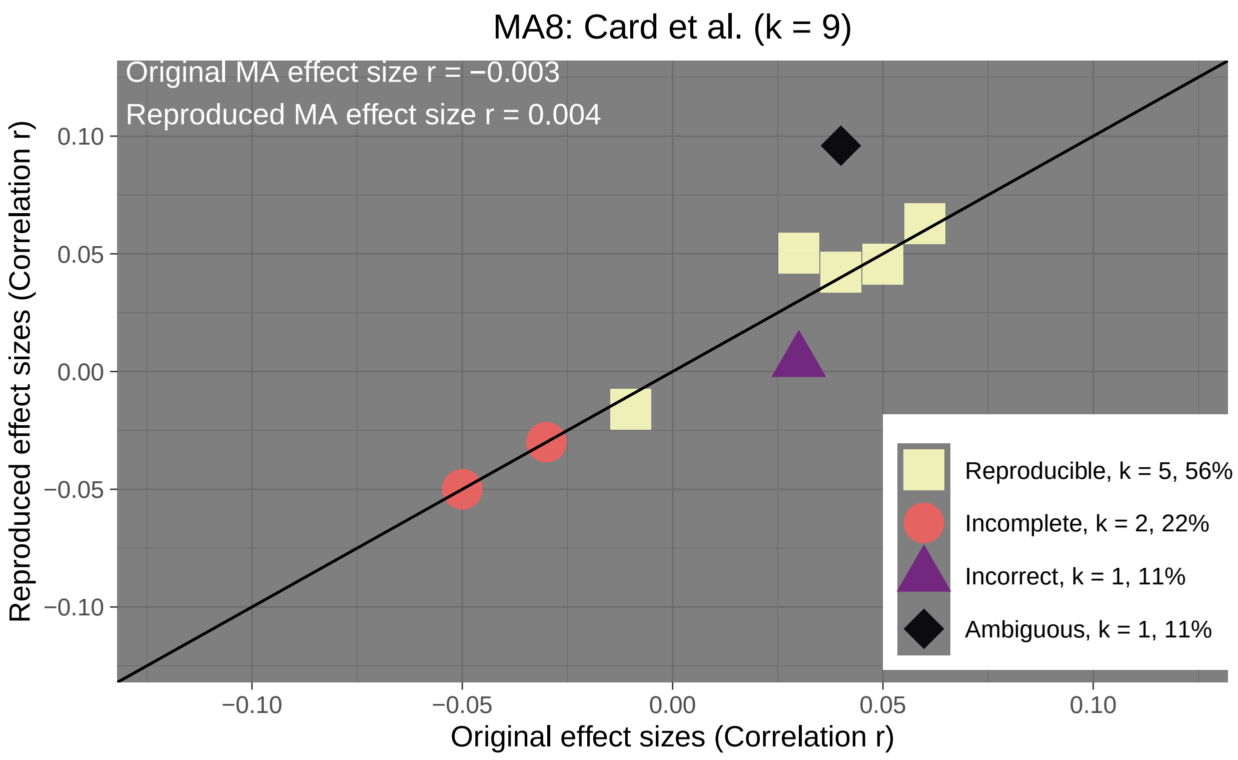
MA8: Card et al. (k = 9)
Original MA effect size r = −0.003
Reproduced MA effect size r = 0.004
Reproduced effect sizes (Correlation r)
Original effect sizes (Correlation r)
0.10
0.05
0.00
−0.05
−0.10
−0.10
−0.05
0.00
0.05
0.10
Reproducible, k = 5, 56%
Incomplete, k = 2, 22%
Incorrect, k = 1, 11%
Ambiguous, k = 1, 11%

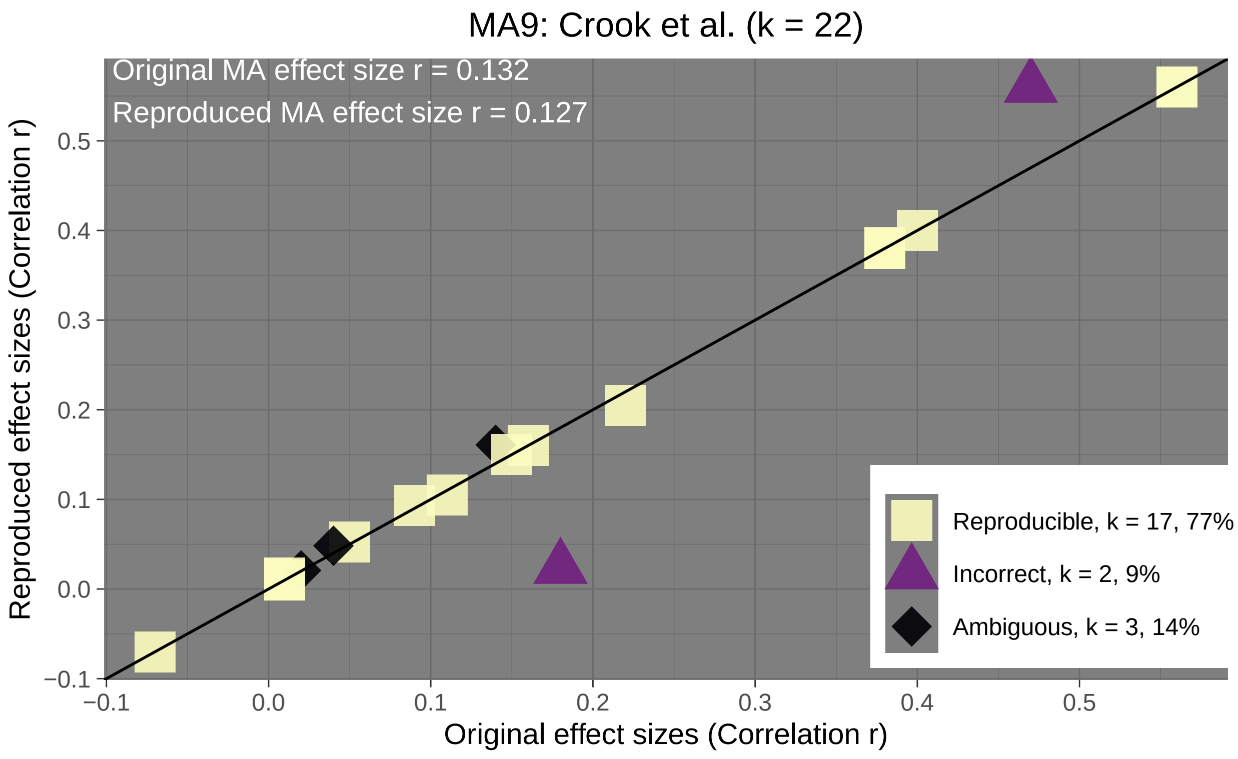
MA9: Crook et al. (k = 22)
Original MA effect size r = 0.132
Reproduced MA effect size r = 0.127
Reproduced effect sizes (Correlation r)
Original effect sizes (Correlation r)
0.5
0.4
0.3
0.2
0.1
0.0
−0.1
−0.1
0.0
0.1
0.2
0.3
0.4
0.5
Reproducible, k = 17, 77%
Incorrect, k = 2, 9%
Ambiguous, k = 3, 14%

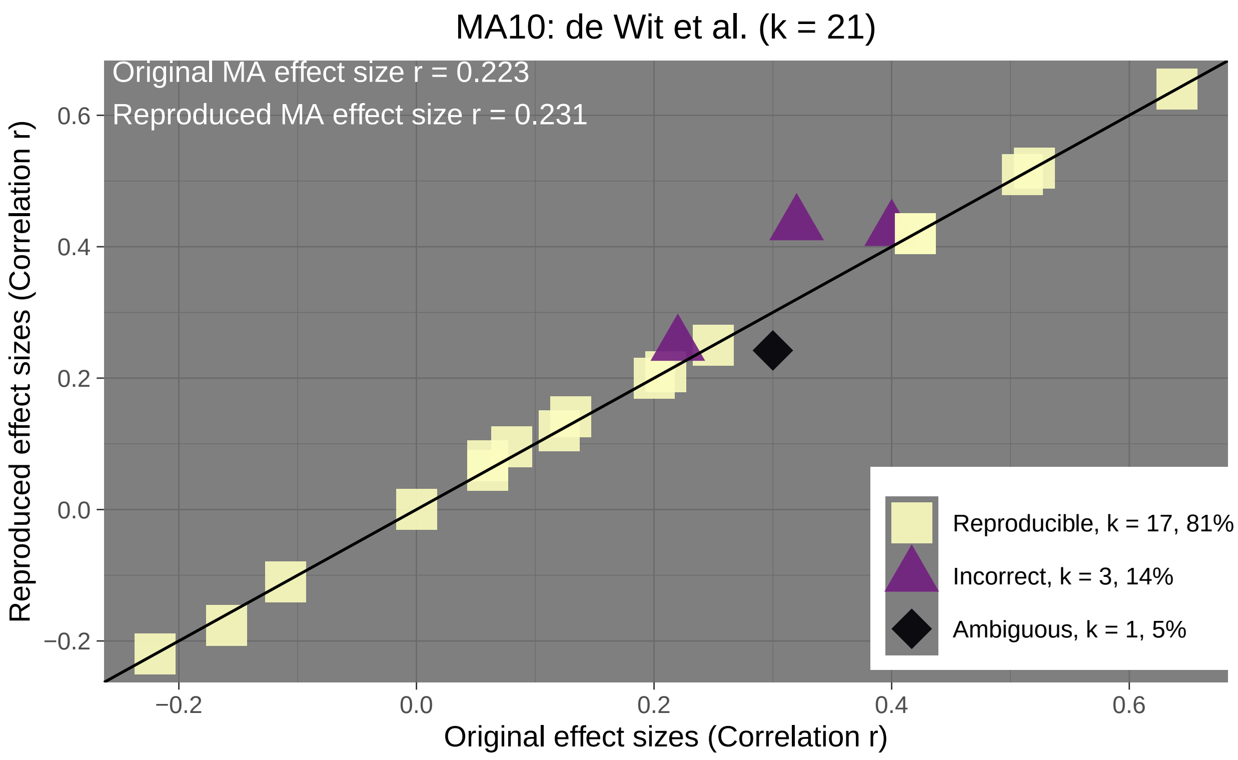
MA10: de Wit et al. (k = 21)
Original MA effect size r = 0.223
Reproduced MA effect size r = 0.231
Reproduced effect sizes (Correlation r)
Original effect sizes (Correlation r)
0.6
0.4
0.2
0.0
−0.2
−0.2
0.0
0.2
0.4
0.6
Reproducible, k = 17, 81%
Incorrect, k = 3, 14%
Ambiguous, k = 1, 5%

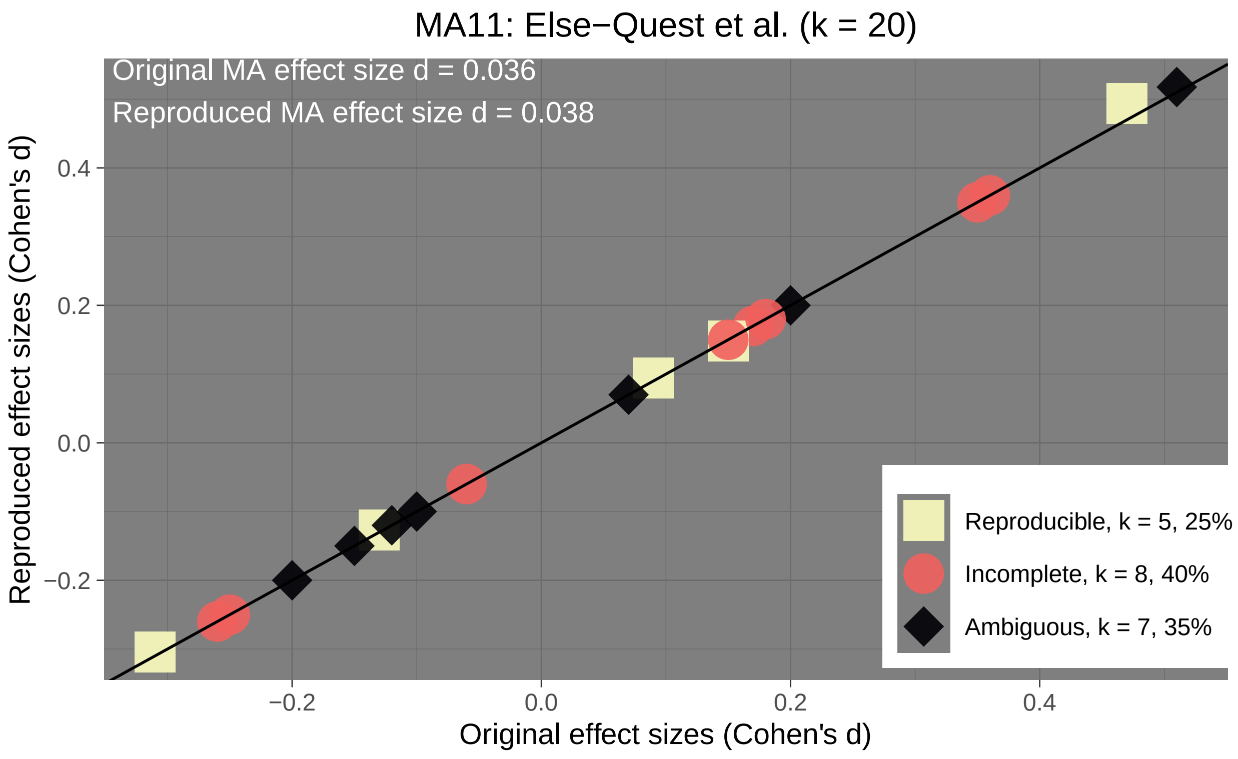
MA11: Else−Quest et al. (k = 20)
Original MA effect size d = 0.036
Reproduced MA effect size d = 0.038
Reproduced effect sizes (Cohen's d)
Original effect sizes (Cohen's d)
0.4
0.2
0.0
−0.2
−0.2
0.0
0.2
0.4
Reproducible, k = 5, 25%
Incomplete, k = 8, 40%
Ambiguous, k = 7, 35%

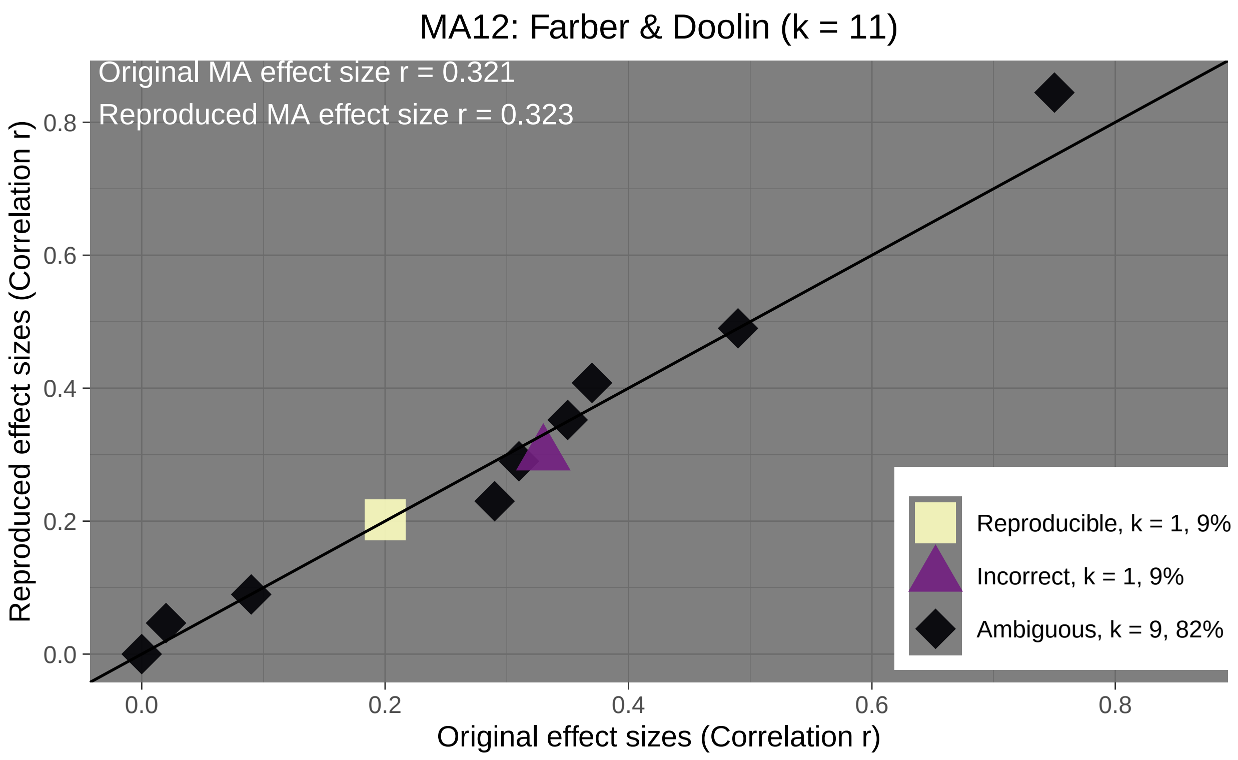
MA12: Farber & Doolin (k = 11)
Original MA effect size r = 0.321
Reproduced MA effect size r = 0.323
Reproduced effect sizes (Correlation r)
Original effect sizes (Correlation r)
Reproducible, k = 1, 9%
Incorrect, k = 1, 9%
Ambiguous, k = 9, 82%

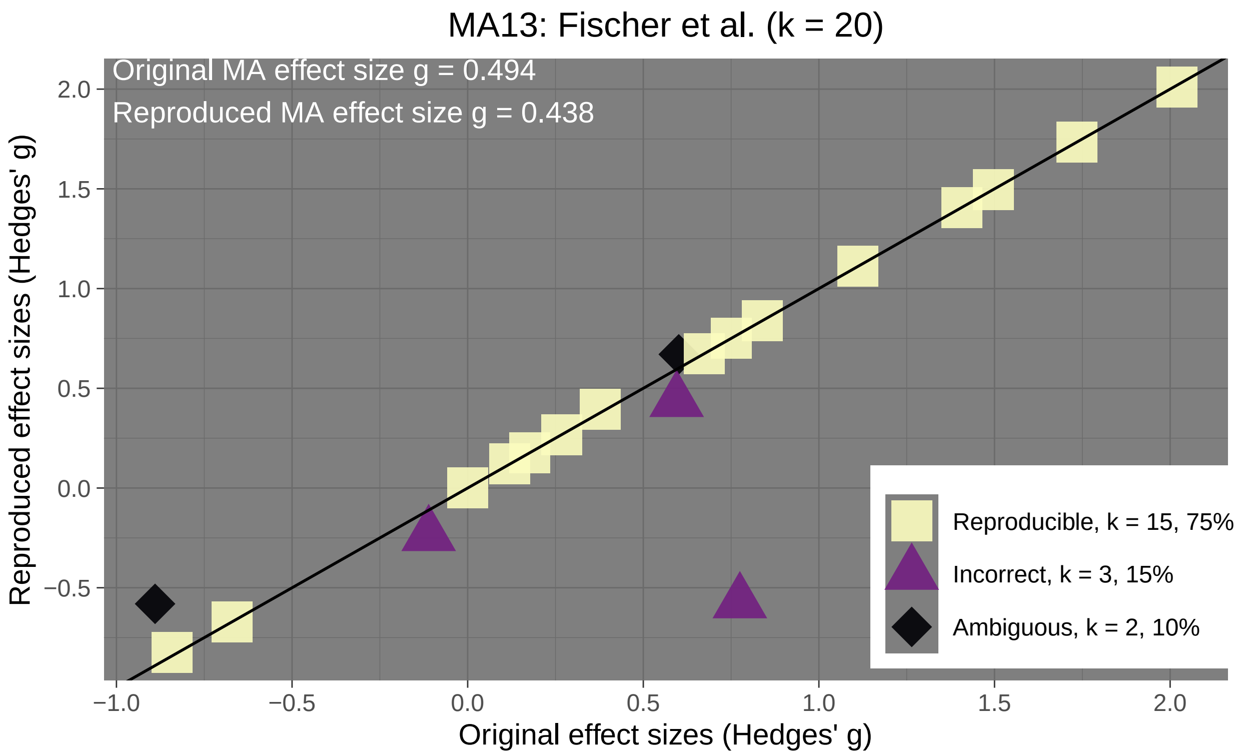
MA13: Fischer et al. (k = 20)
Original MA effect size g = 0.494
Reproduced MA effect size g = 0.438
Reproduced effect sizes (Hedges' g)
Original effect sizes (Hedges' g)
Reproducible, k = 15, 75%
Incorrect, k = 3, 15%
Ambiguous, k = 2, 10%

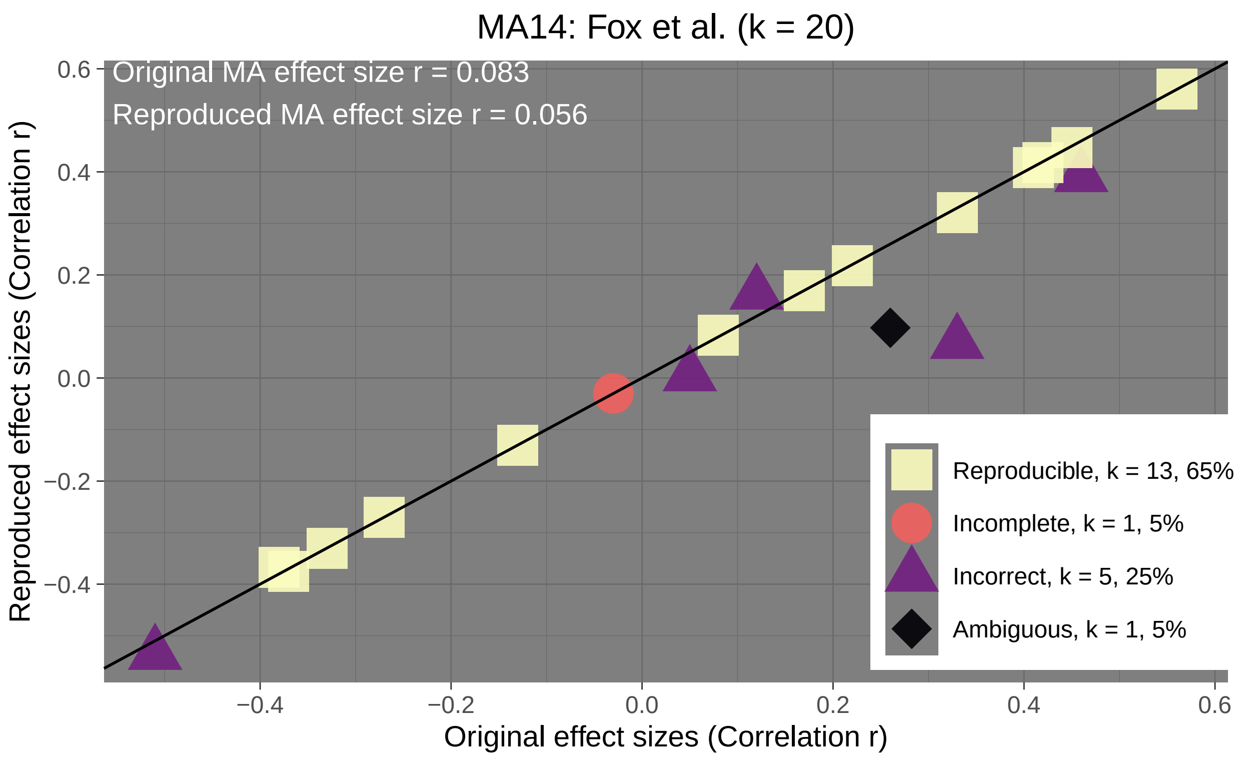
MA14: Fox et al. (k = 20)
Original MA effect size r = 0.083
Reproduced MA effect size r = 0.056
Reproducible, k = 13, 65%
Incomplete, k = 1, 5%
Incorrect, k = 5, 25%
Ambiguous, k = 1, 5%
Reproduced effect sizes (Correlation r)
Original effect sizes (Correlation r)

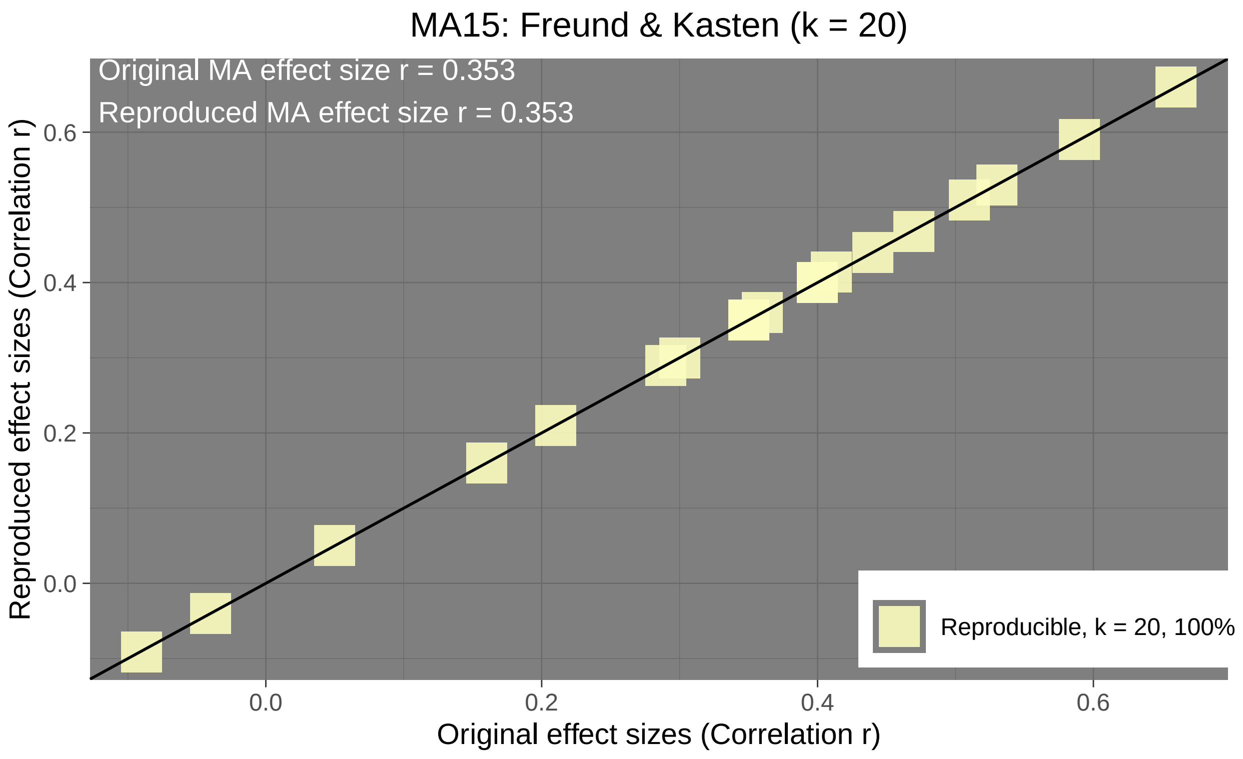
MA15: Freund & Kasten (k = 20)
Original MA effect size r = 0.353
Reproduced MA effect size r = 0.353
Reproducible, k = 20, 100%
Reproduced effect sizes (Correlation r)
Original effect sizes (Correlation r)

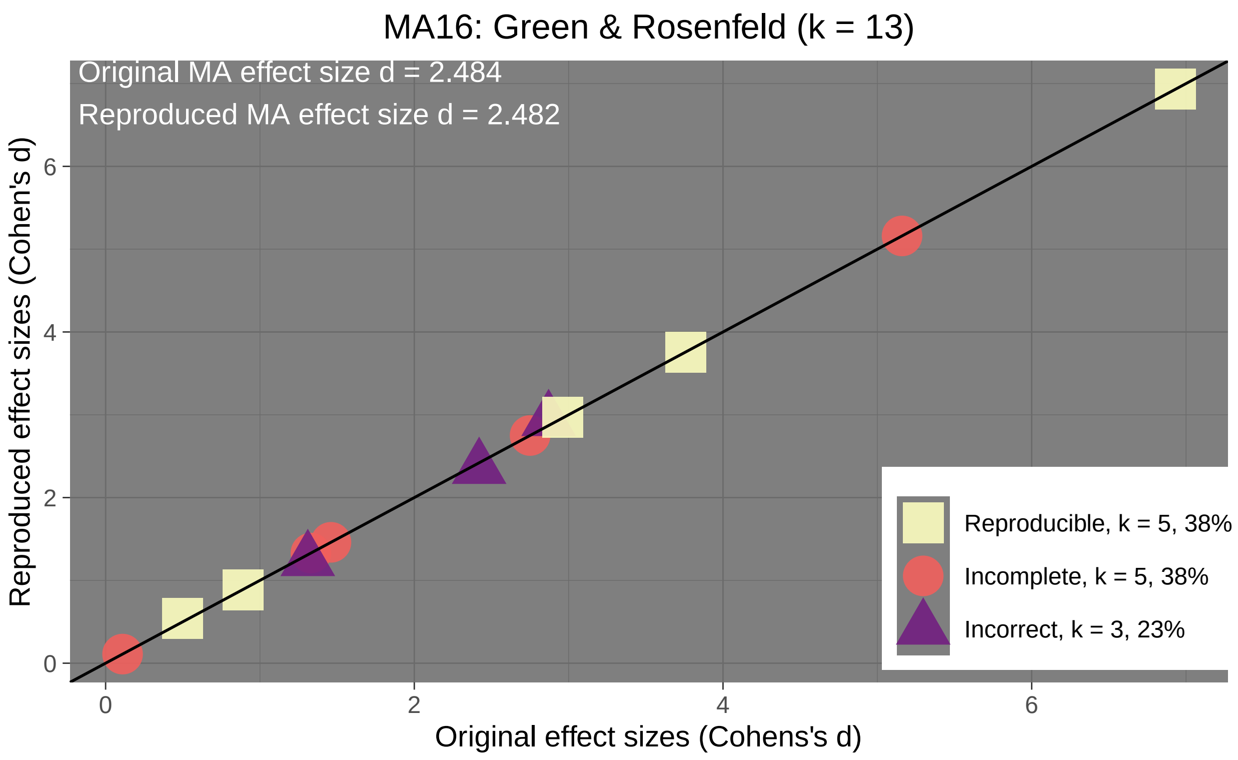
MA16: Green & Rosenfeld (k = 13)
Original MA effect size d = 2.484
Reproduced MA effect size d = 2.482
Reproduced effect sizes (Cohen's d)
Original effect sizes (Cohens's d)
Reproducible, k = 5, 38%
Incomplete, k = 5, 38%
Incorrect, k = 3, 23%

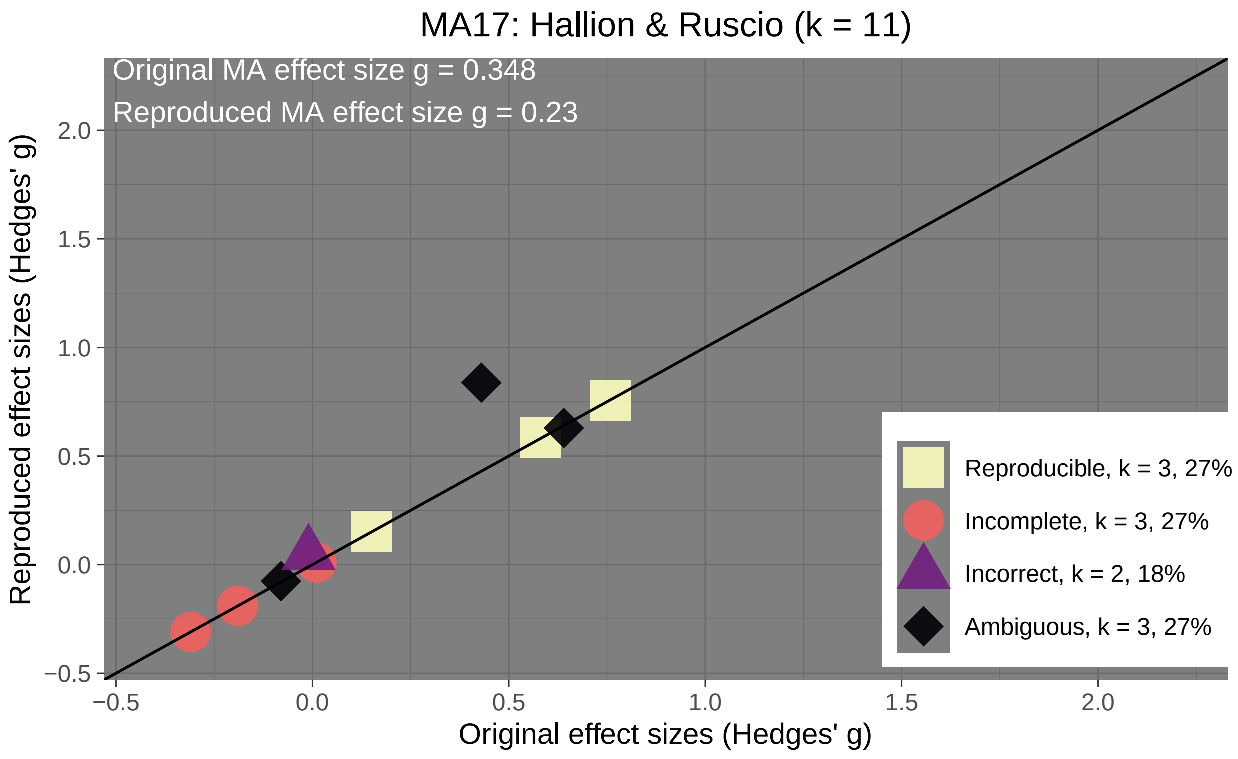
MA17: Hallion & Ruscio (k = 11)
Original MA effect size g = 0.348
Reproduced MA effect size g = 0.23
Reproduced effect sizes (Hedges' g)
Original effect sizes (Hedges' g)
Reproducible, k = 3, 27%
Incomplete, k = 3, 27%
Incorrect, k = 2, 18%
Ambiguous, k = 3, 27%

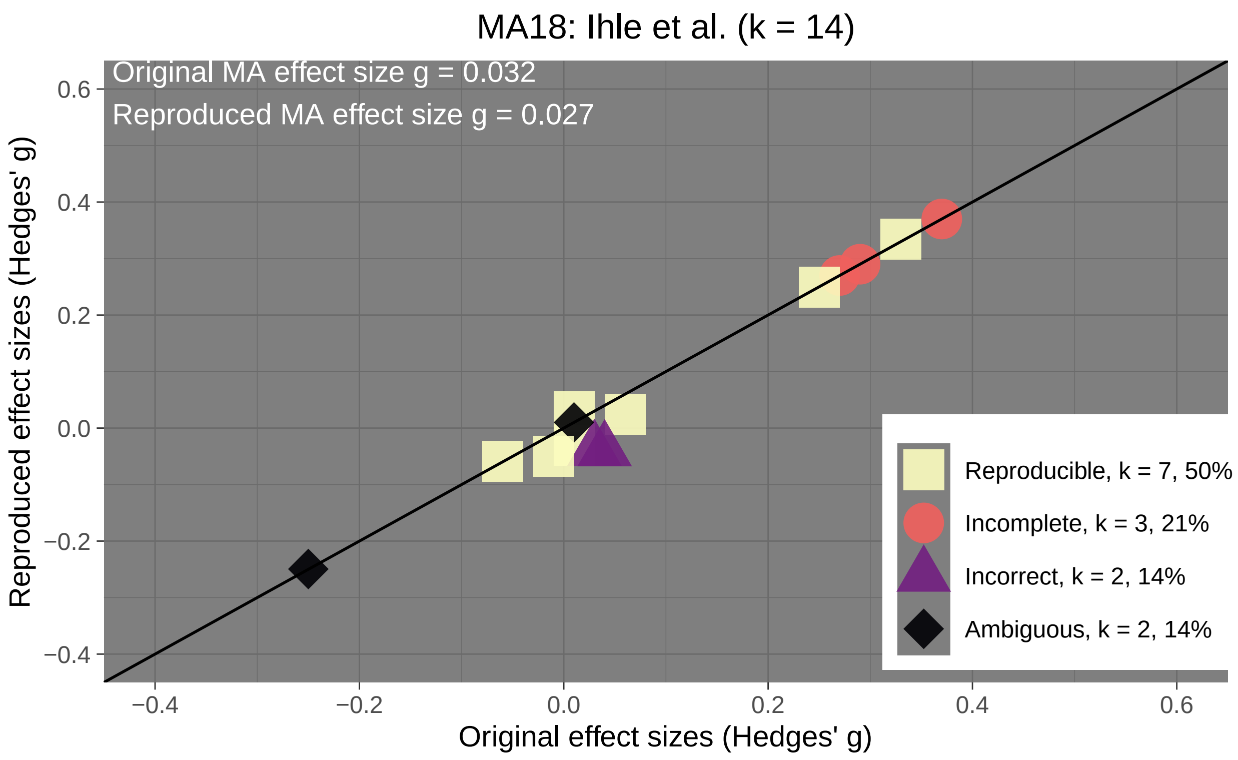
MA18: Ihle et al. (k = 14)
Original MA effect size g = 0.032
Reproduced MA effect size g = 0.027
Reproducible, k = 7, 50%
Incomplete, k = 3, 21%
Incorrect, k = 2, 14%
Ambiguous, k = 2, 14%
Reproduced effect sizes (Hedges' g)
Original effect sizes (Hedges' g)
0.6
0.4
0.2
0.0
−0.2
−0.4
−0.4
−0.2
0.0
0.2
0.4
0.6

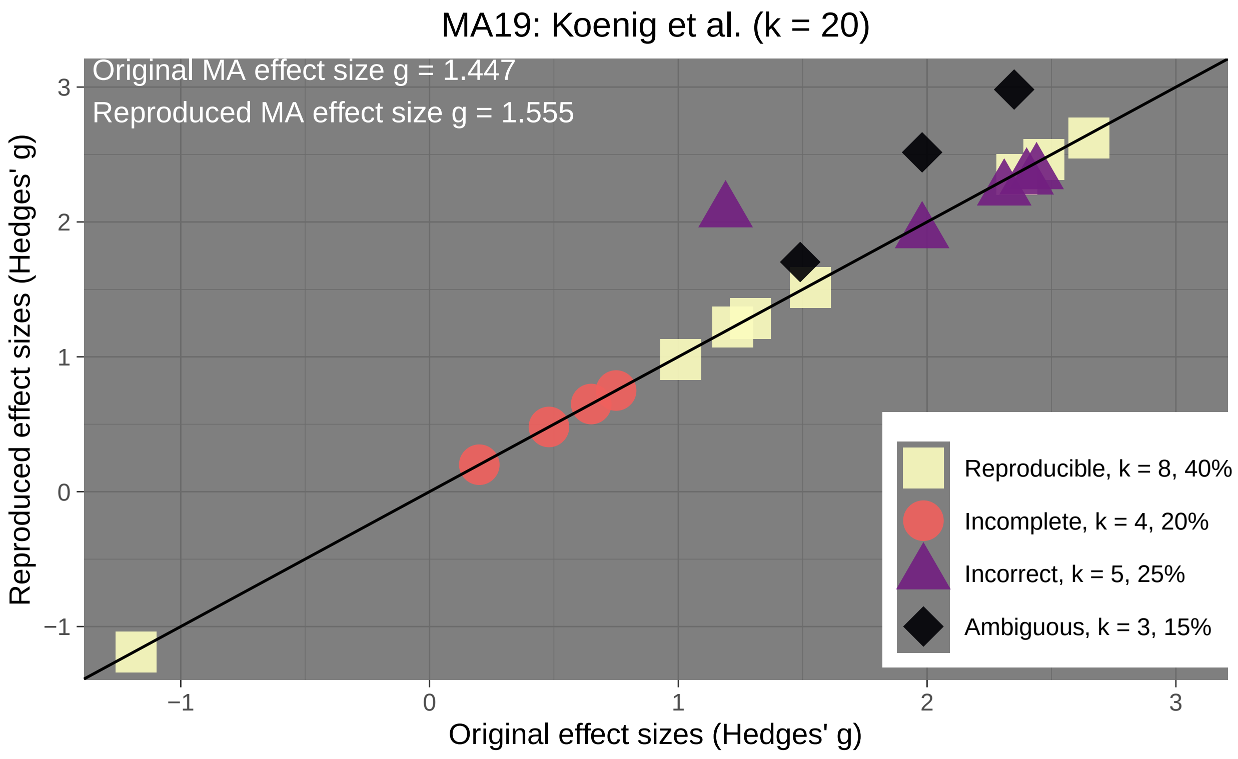
MA19: Koenig et al. (k = 20)
Original MA effect size g = 1.447
Reproduced MA effect size g = 1.555
Reproducible, k = 8, 40%
Incomplete, k = 4, 20%
Incorrect, k = 5, 25%
Ambiguous, k = 3, 15%
Reproduced effect sizes (Hedges' g)
Original effect sizes (Hedges' g)
3
2
1
0
−1
−1
0
1
2
3

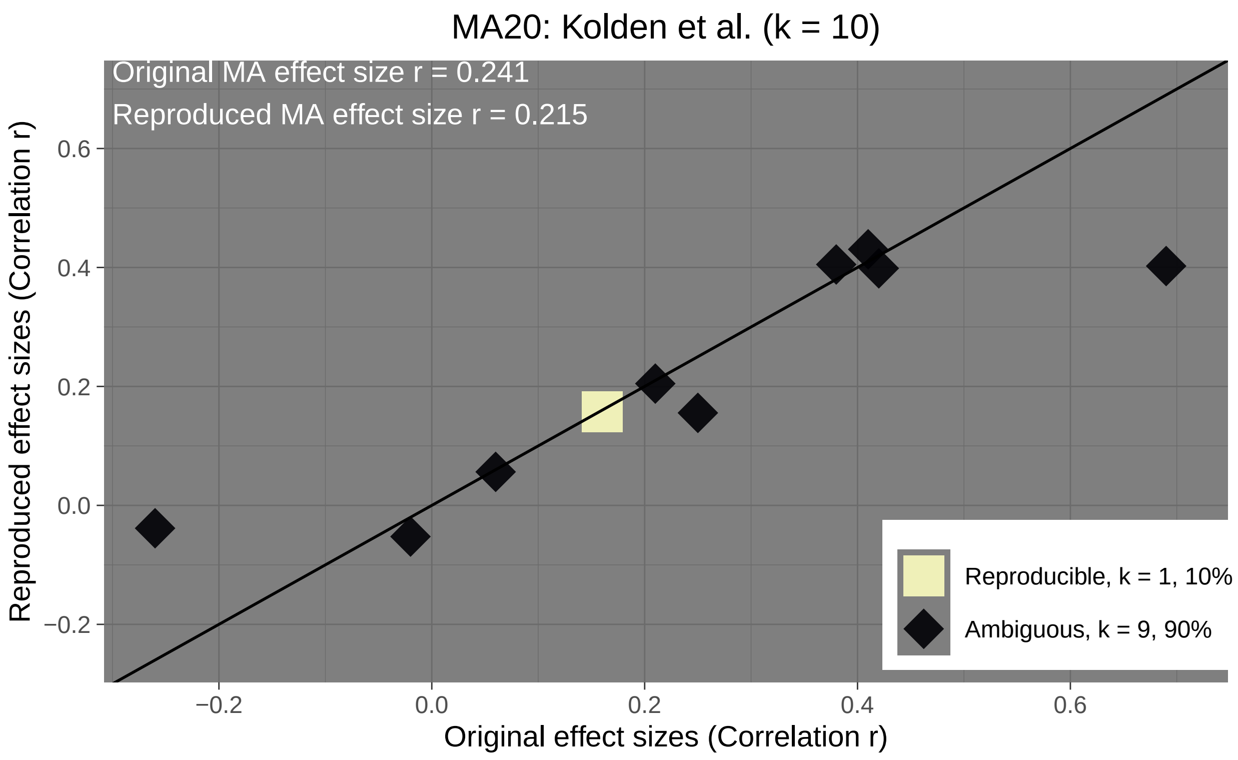
MA20: Kolden et al. (k = 10)
Original MA effect size r = 0.241
Reproduced MA effect size r = 0.215
Reproduced effect sizes (Correlation r)
0.6
0.4
0.2
0.0
−0.2
−0.2
0.0
0.2
0.4
0.6
Original effect sizes (Correlation r)
Reproducible, k = 1, 10%
Ambiguous, k = 9, 90%

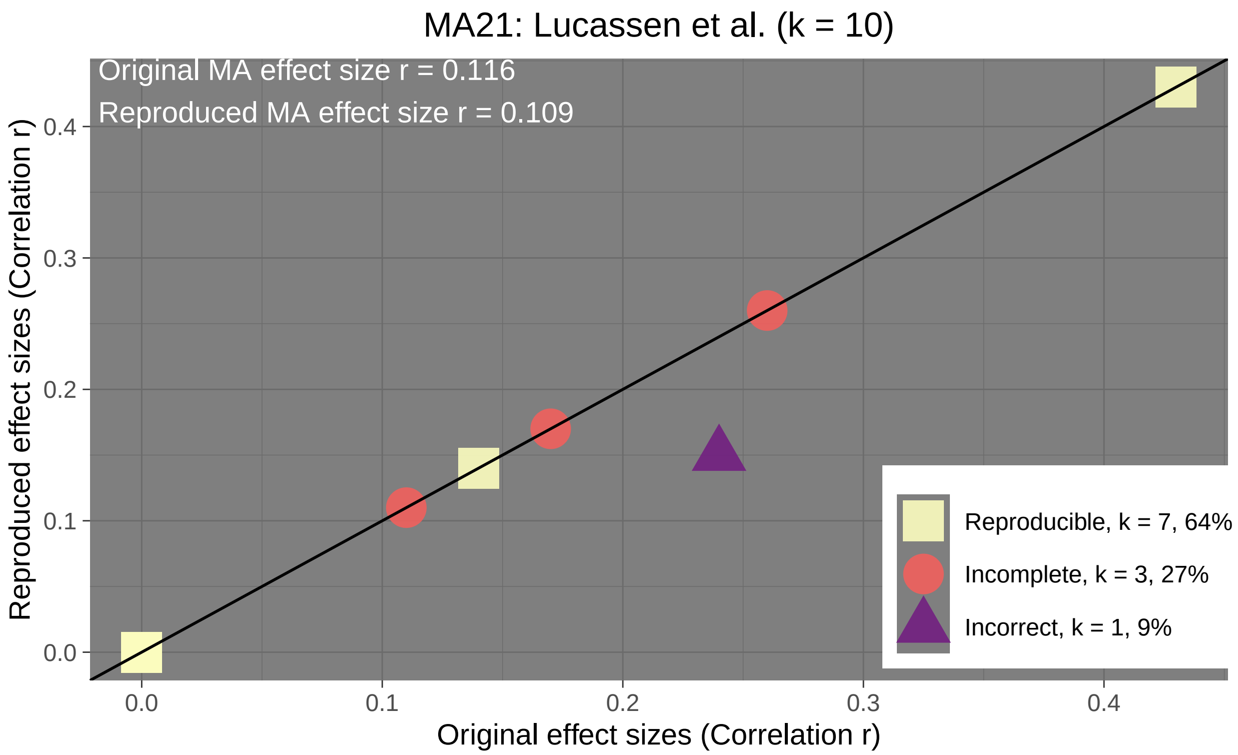
MA21: Lucassen et al. (k = 10)
Original MA effect size r = 0.116
Reproduced MA effect size r = 0.109
Reproduced effect sizes (Correlation r)
0.4
0.3
0.2
0.1
0.0
0.0
0.1
0.2
0.3
0.4
Original effect sizes (Correlation r)
Reproducible, k = 7, 64%
Incomplete, k = 3, 27%
Incorrect, k = 1, 9%

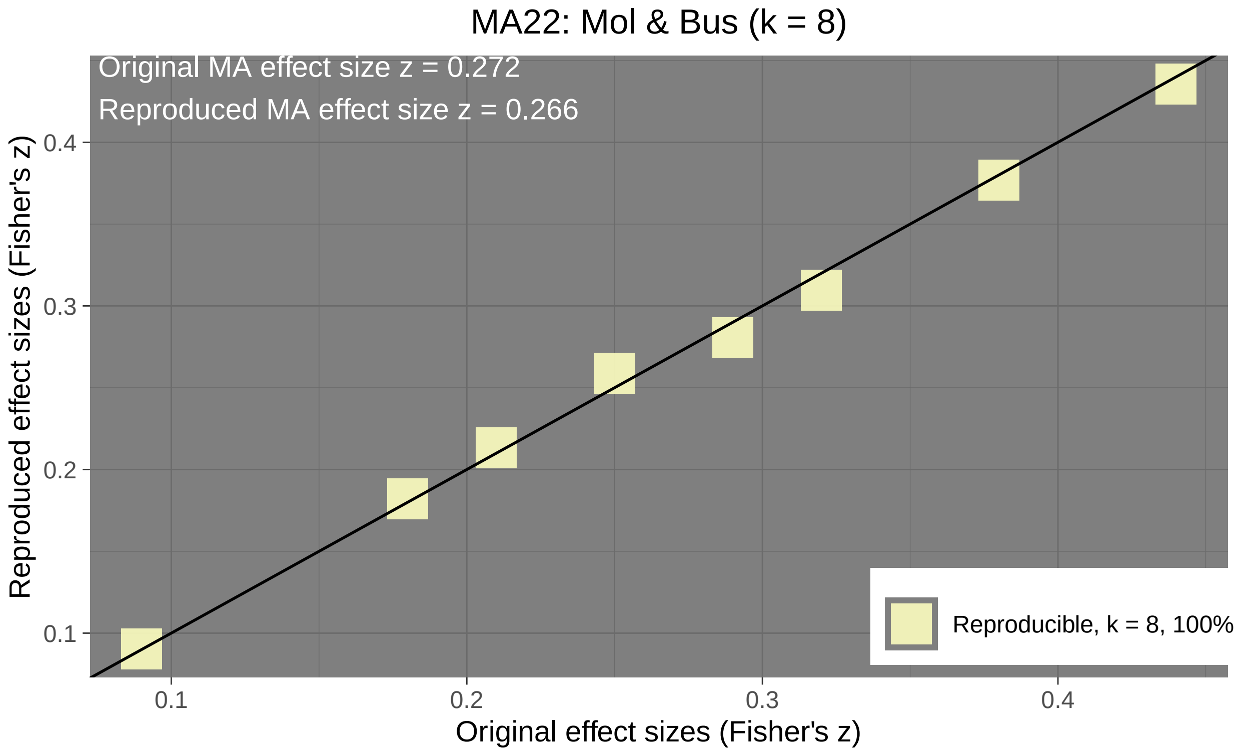
MA22: Mol & Bus (k = 8)
Original MA effect size z = 0.272
Reproduced MA effect size z = 0.266
Reproduced effect sizes (Fisher's z)
0.4
0.3
0.2
0.1
0.1
0.2
0.3
0.4
Original effect sizes (Fisher's z)
Reproducible, k = 8, 100%

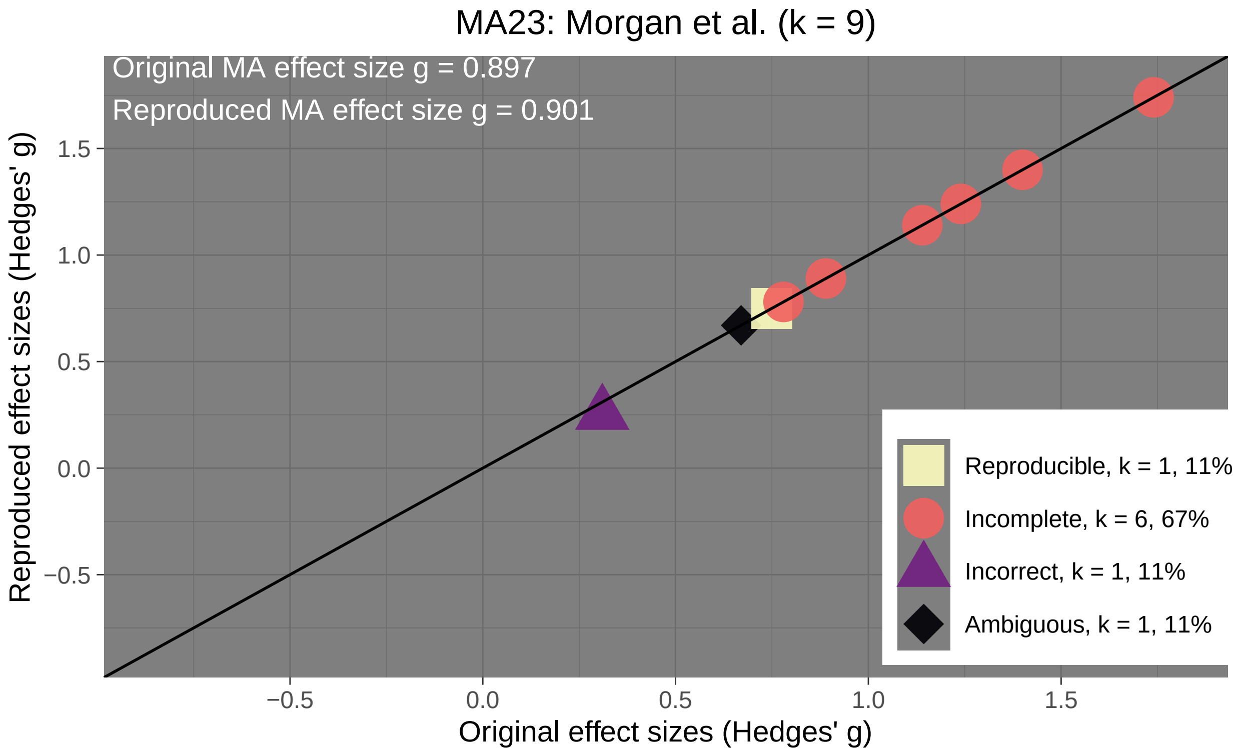
MA23: Morgan et al. (k = 9)
Original MA effect size g = 0.897
Reproduced MA effect size g = 0.901
Reproduced effect sizes (Hedges' g)
1.5
1.0
0.5
0.0
−0.5
−0.5
0.0
0.5
1.0
1.5
Original effect sizes (Hedges' g)
Reproducible, k = 1, 11%
Incomplete, k = 6, 67%
Incorrect, k = 1, 11%
Ambiguous, k = 1, 11%

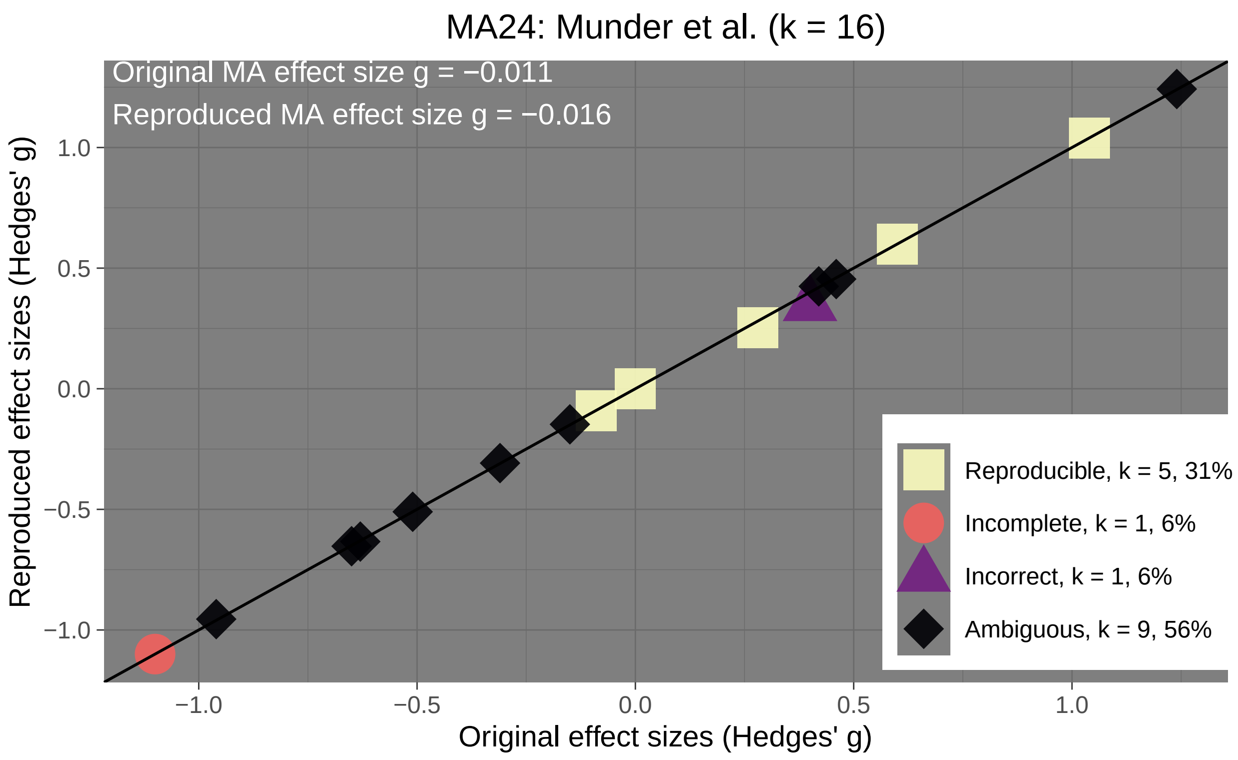
MA24: Munder et al. (k = 16)
Original MA effect size g = −0.011
Reproduced MA effect size g = −0.016
Reproduced effect sizes (Hedges' g)
Original effect sizes (Hedges' g)
1.0
0.5
0.0
−0.5
−1.0
Reproducible, k = 5, 31%
Incomplete, k = 1, 6%
Incorrect, k = 1, 6%
Ambiguous, k = 9, 56%

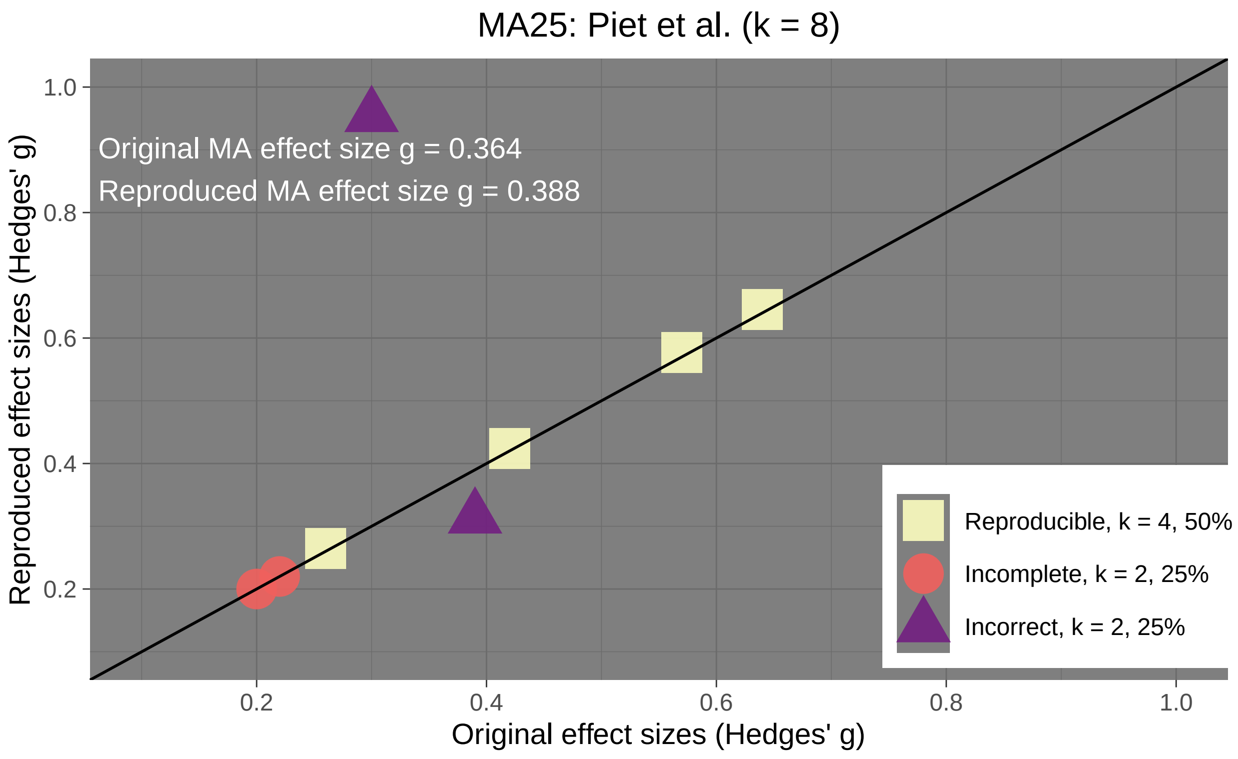
MA25: Piet et al. (k = 8)
Original MA effect size g = 0.364
Reproduced MA effect size g = 0.388
Reproduced effect sizes (Hedges' g)
Original effect sizes (Hedges' g)
1.0
0.8
0.6
0.4
0.2
Reproducible, k = 4, 50%
Incomplete, k = 2, 25%
Incorrect, k = 2, 25%

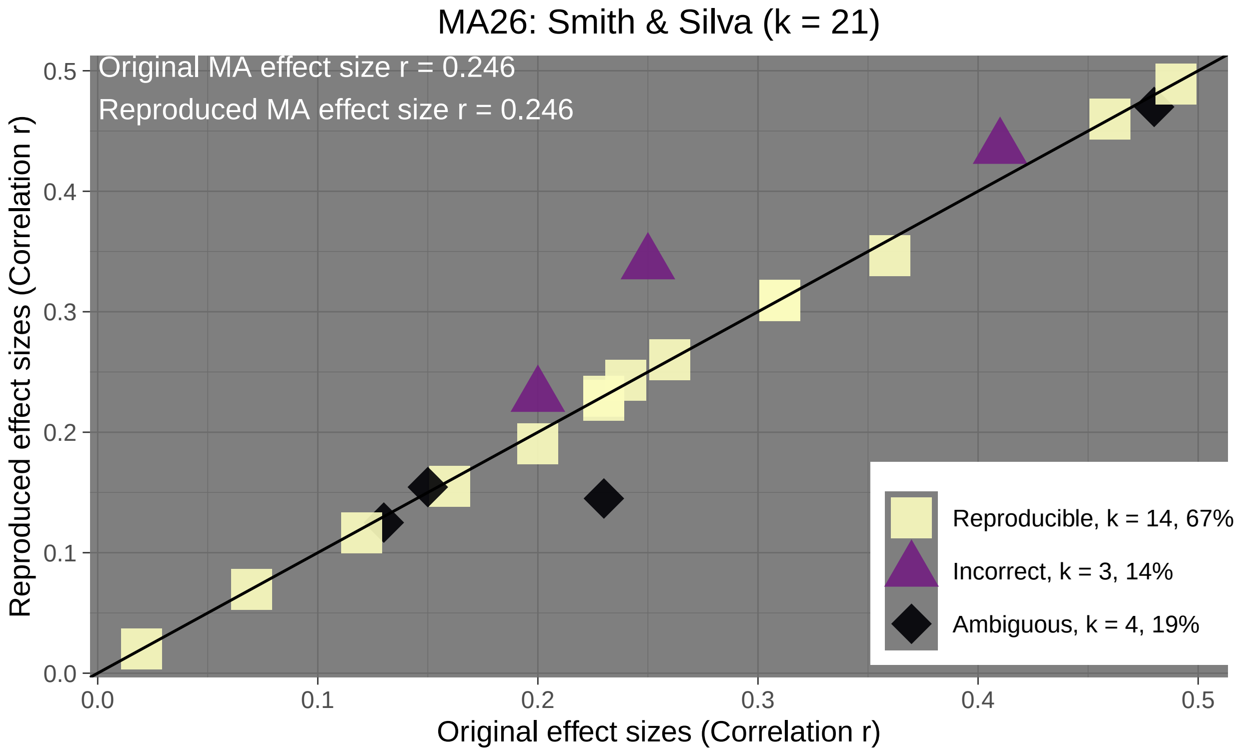
MA26: Smith & Silva (k = 21)
Original MA effect size r = 0.246
Reproduced MA effect size r = 0.246
Reproduced effect sizes (Correlation r)
Original effect sizes (Correlation r)
0.0
0.1
0.2
0.3
0.4
0.5
Reproducible, k = 14, 67%
Incorrect, k = 3, 14%
Ambiguous, k = 4, 19%

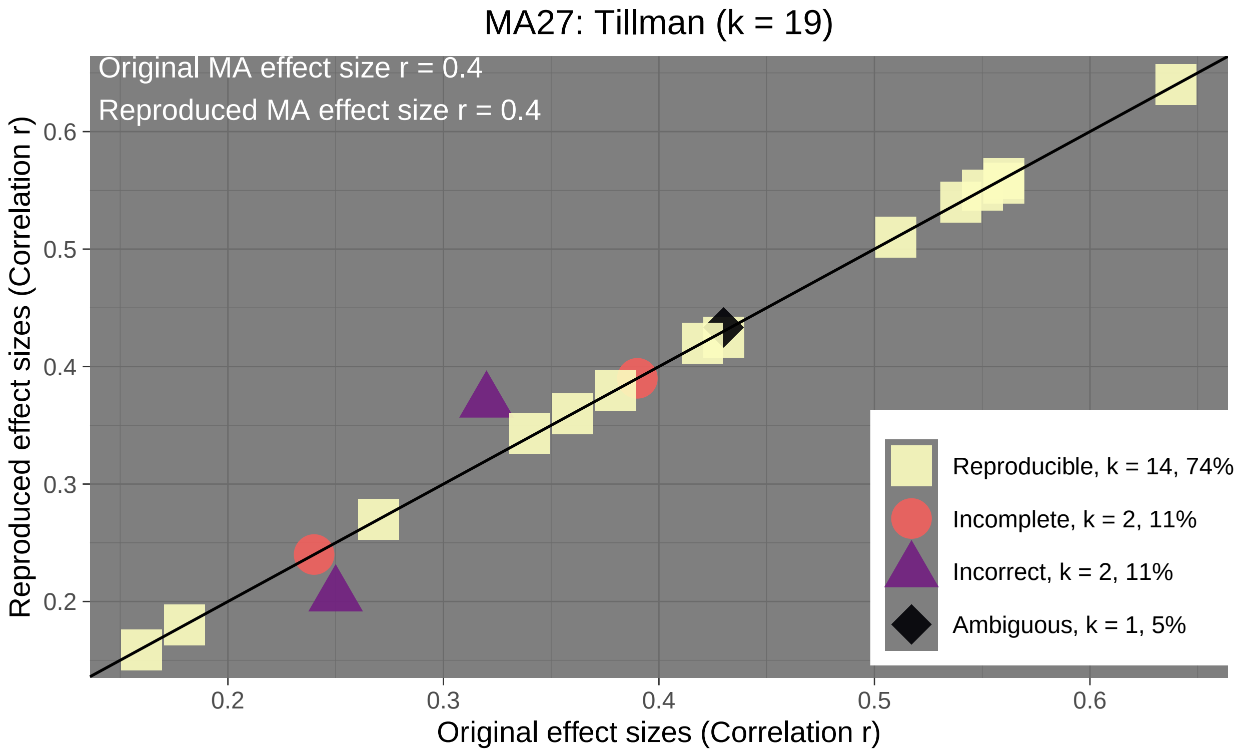
MA27: Tillman (k = 19)
Original MA effect size r = 0.4
Reproduced MA effect size r = 0.4
Reproduced effect sizes (Correlation r)
Original effect sizes (Correlation r)
0.2
0.3
0.4
0.5
0.6
Reproducible, k = 14, 74%
Incomplete, k = 2, 11%
Incorrect, k = 2, 11%
Ambiguous, k = 1, 5%

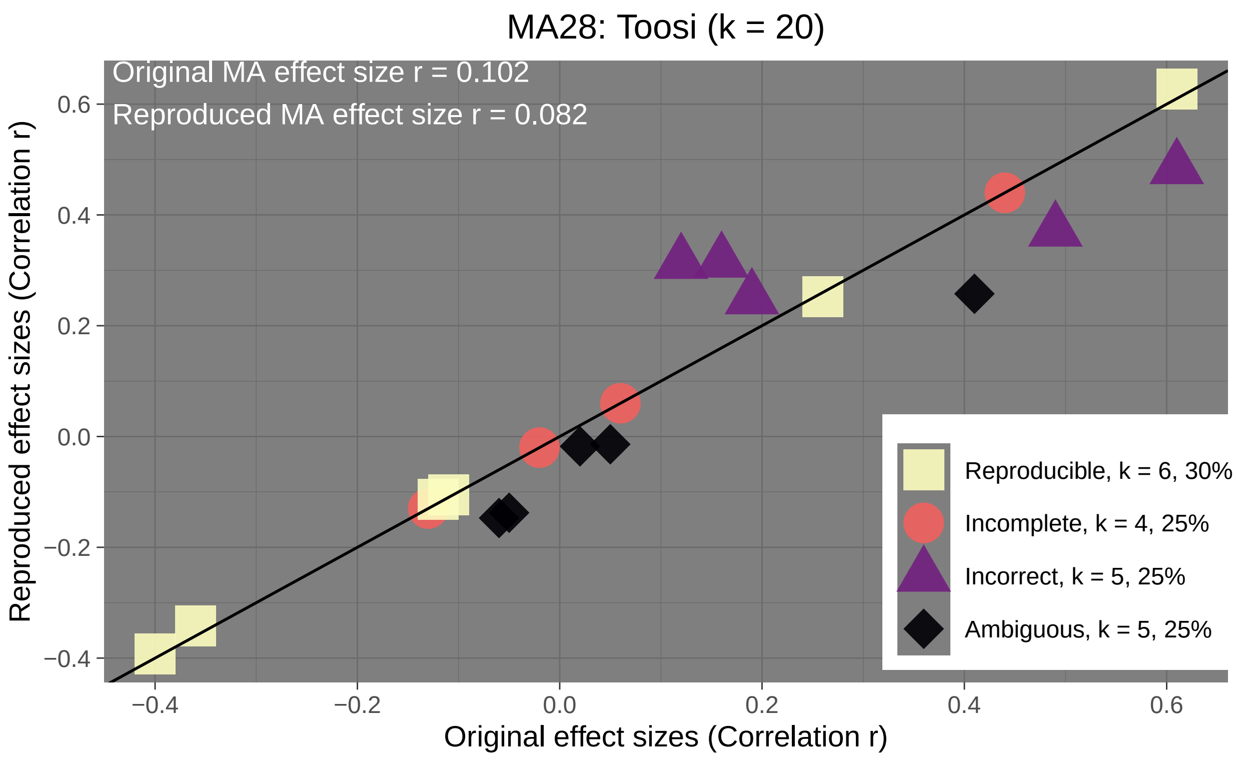
MA28: Toosi (k = 20)
Original MA effect size r = 0.102
Reproduced MA effect size r = 0.082
Reproducible, k = 6, 30%
Incomplete, k = 4, 25%
Incorrect, k = 5, 25%
Ambiguous, k = 5, 25%
Reproduced effect sizes (Correlation r)
Original effect sizes (Correlation r)

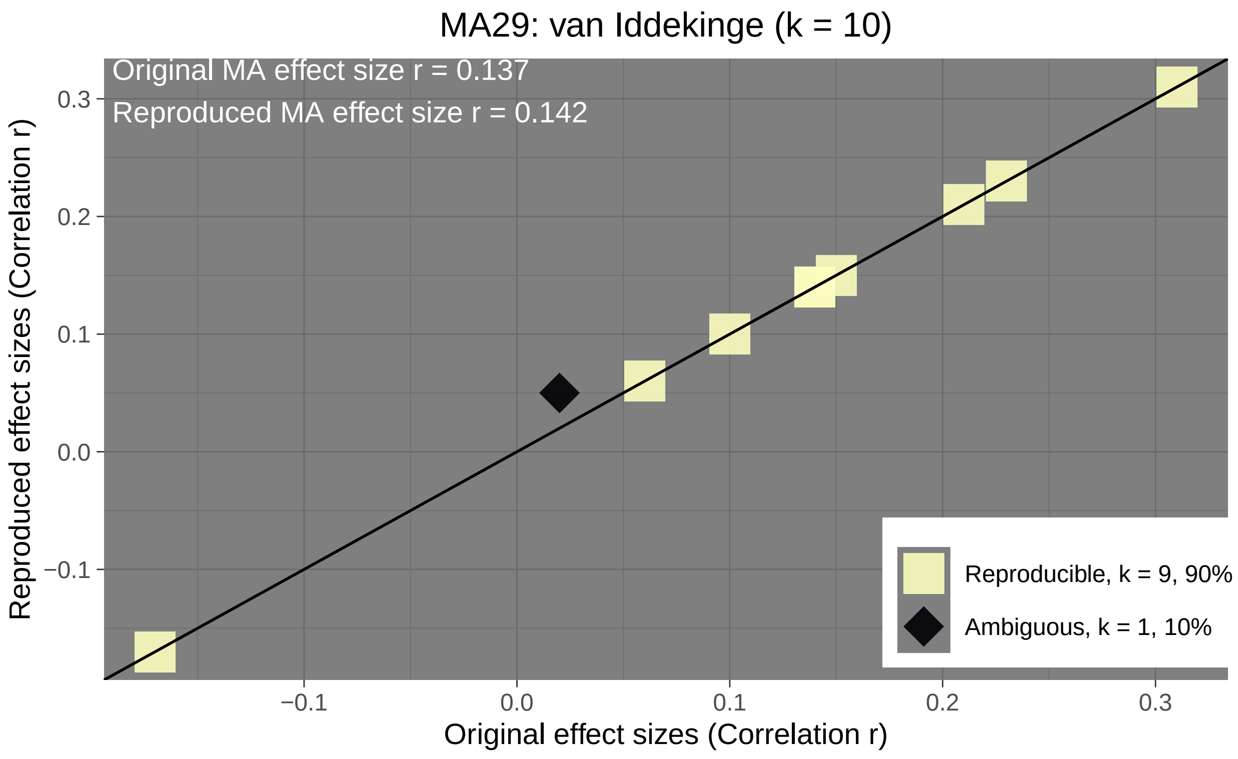
MA29: van Iddekinge (k = 10)
Original MA effect size r = 0.137
Reproduced MA effect size r = 0.142
Reproducible, k = 9, 90%
Ambiguous, k = 1, 10%
Reproduced effect sizes (Correlation r)
Original effect sizes (Correlation r)

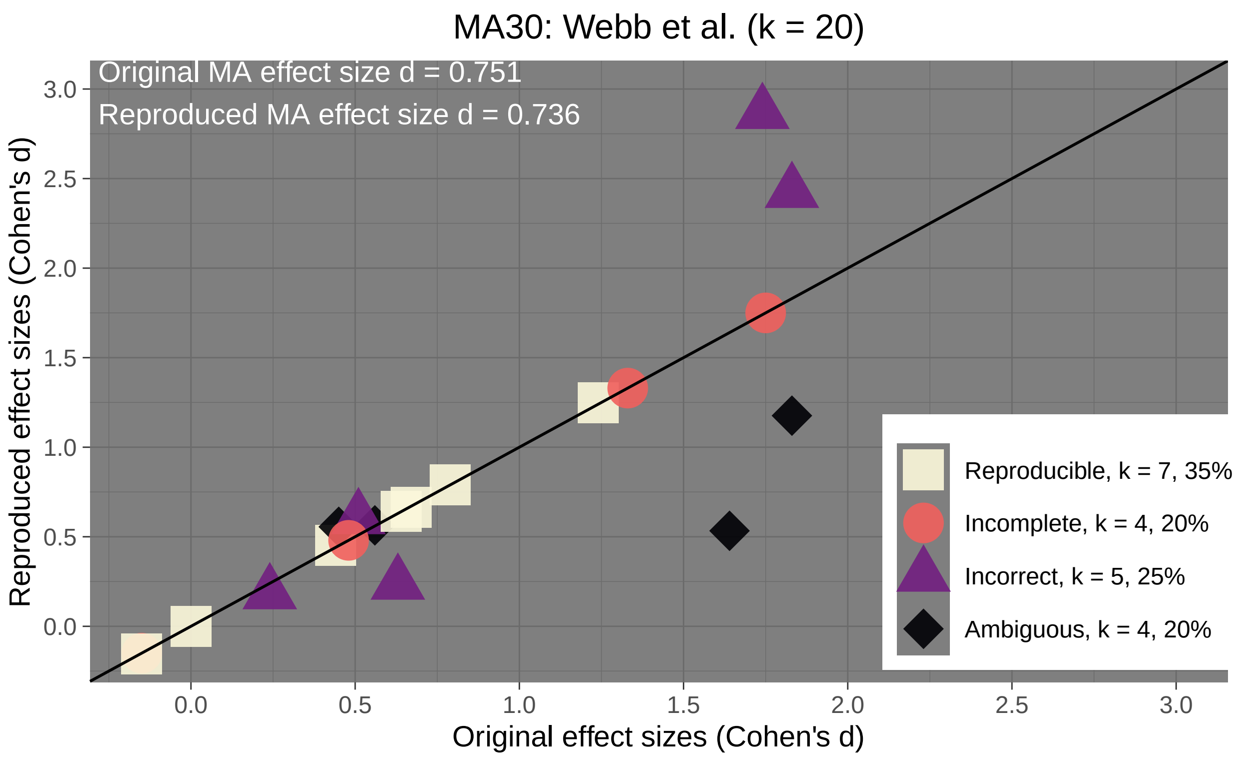
MA30: Webb et al. (k = 20)
Original MA effect size d = 0.751
Reproduced MA effect size d = 0.736
Reproducible, k = 7, 35%
Incomplete, k = 4, 20%
Incorrect, k = 5, 25%
Ambiguous, k = 4, 20%
Reproduced effect sizes (Cohen's d)
Original effect sizes (Cohen's d)

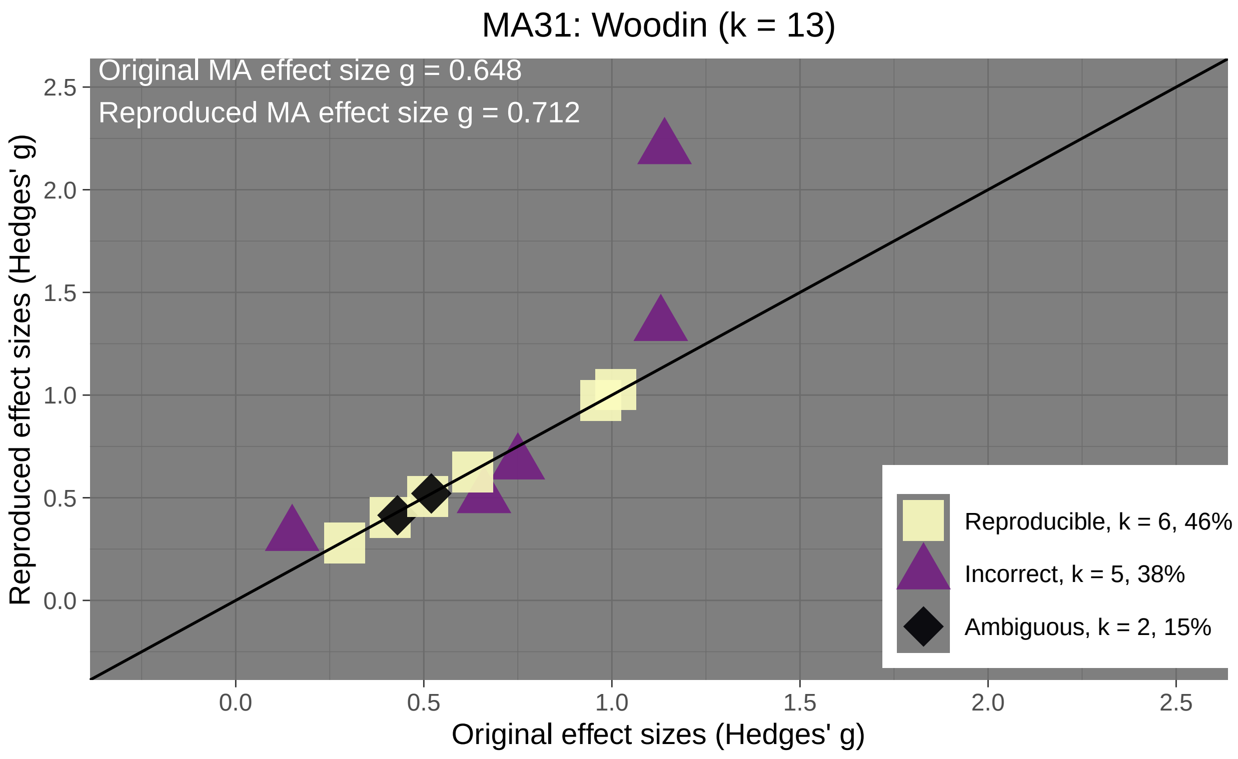
MA31: Woodin (k = 13)
Original MA effect size g = 0.648
Reproduced MA effect size g = 0.712
Reproducible, k = 6, 46%
Incorrect, k = 5, 38%
Ambiguous, k = 2, 15%
Reproduced effect sizes (Hedges' g)
Original effect sizes (Hedges' g)

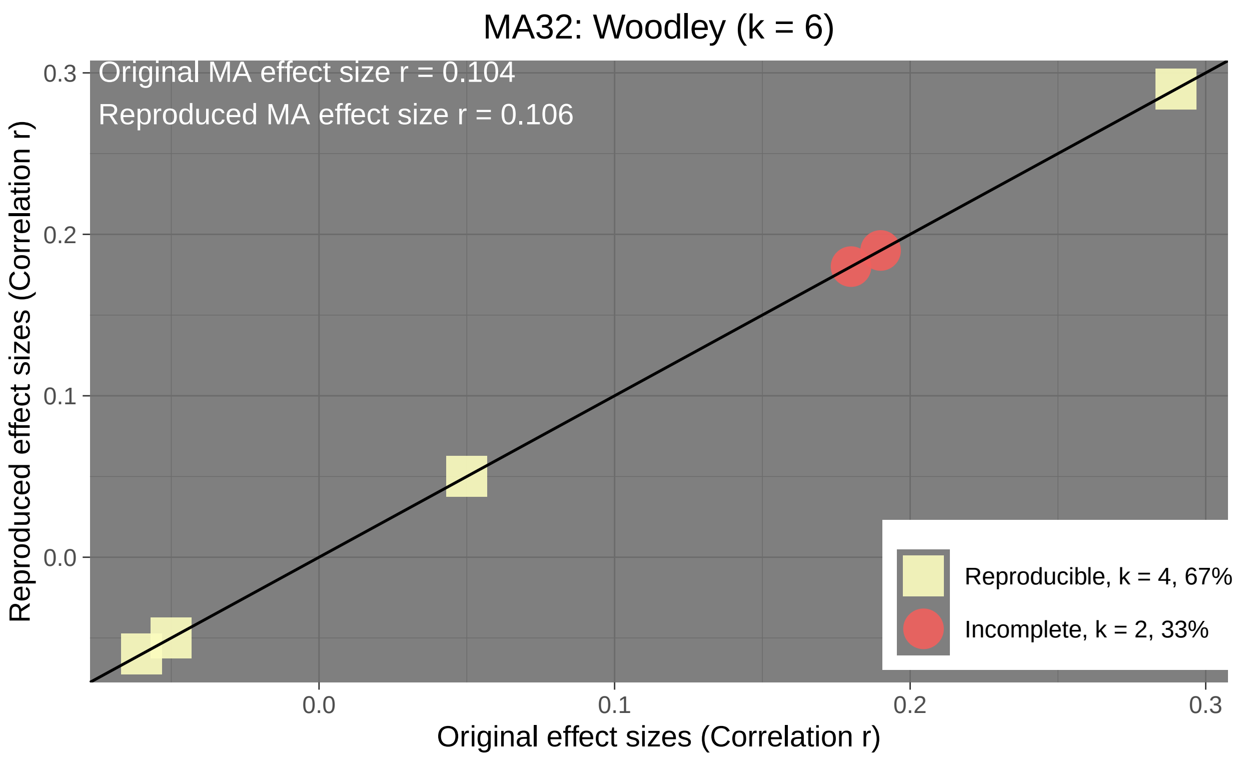
MA32: Woodley (k = 6)
Original MA effect size r = 0.104
Reproduced MA effect size r = 0.106
Reproducible, k = 4, 67%
Incomplete, k = 2, 33%
Reproduced effect sizes (Correlation r)
Original effect sizes (Correlation r)
0.3
0.2
0.1
0.0
0.0
0.1
0.2
0.3

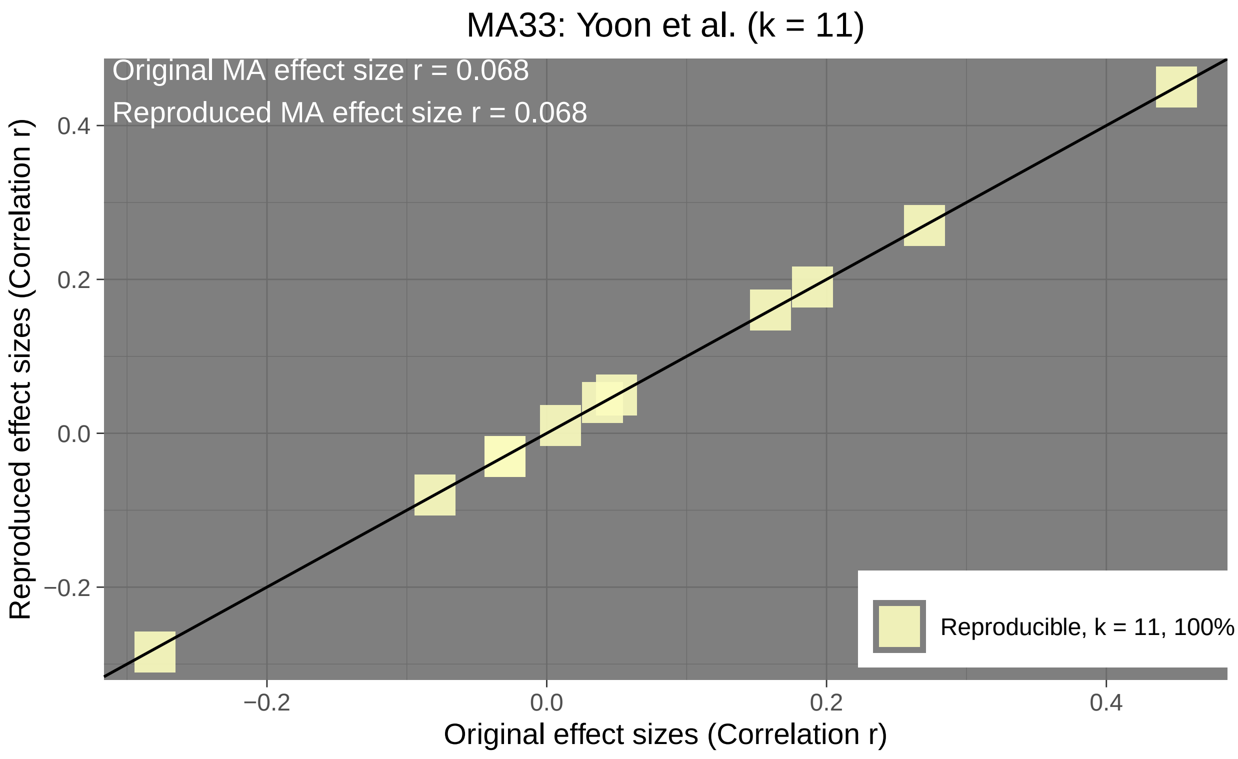
MA33: Yoon et al. (k = 11)
Original MA effect size r = 0.068
Reproduced MA effect size r = 0.068
Reproducible, k = 11, 100%
Reproduced effect sizes (Correlation r)
Original effect sizes (Correlation r)
0.4
0.2
0.0
−0.2
−0.2
0.0
0.2
0.4
