# Supplementary material for: Reproducibility of individual effect sizes in meta-analyses in psychology
Source: PLoS One. 2020 May 27;15(5):e0233107. doi: 10.1371/journal.pone.0233107 (PMC7252608; doi:10.1371/journal.pone.0233107)
Supplement: S5 File — https://osf.io/u2j3z/. (PDF) [file pone.0233107.s005.pdf]

Reproducibility of Individual Effect Sizes in Meta-Analyses in Psychology
Supplement E
Post stratification calculations

Supplement E contains a detailed explanation on how we corrected for the oversampling of outliers.

Some data errors in meta-analyses are likely to result in outliers, which inflate variance estimates (Hunter and Schmidt, 2004, p.66). Because of this, we oversampled outlier primary studies to investigate whether outliers were more likely to be erroneous compared to non-outlier primary studies.

Table 1.*Population distribution of (non-)outlier primary studies.*

	no. of primary studies	
**non-outlier**	1382	70%
**outlier**	596	30%
Total	1978	100%

Table 2.*Sample distribution of (non-)outlier primary studies.*

	no. of primary studies	
**non-outlier**	303	61%
**outlier**	197	39%
Total	500	100%

Because we oversampled outliers, our sample is not representative for the population. Presented in Table 1 and Table 2 are the percentages of primary studies that have effect sizes classified as (non-)outliers in the population (our sample of 33 meta-analyses) and sample (our sample of 500 primary studies). In the population, 30% of effect sizes classified as an outlier, compared to 39% of our sample, meaning our sample contained too many outlier primary study effect sizes, and too few non-outliers. In order to estimate the probability that any given effect size in a certain meta-analysis is incorrect, we need to deal with this overrepresentation of outliers by design. We adjusted the sample proportions for each meta-analysis separately, because the relative number of (non-)outlier primary studies differs across meta-analyses, as do the number of selected studies. After adjustment, the sample proportions of each meta-analysis are in line with their corresponding population proportions. Meta-analysis 1 will be used as an example throughout this document.

As presented in Table 3, 35% of primary study effect sizes in meta-analysis 1 were outliers, but Table 4 shows outlier primary study effect sizes take up 50%

Table 3. *Population distribution of (non-)outlier primary studies in meta-analysis 1.*

	no. of primary studies in MA1	
**non-outlier**	37	65%
**outlier**	20	35%
Total	57	100%

Table 4. *Sample distribution of (non-)outlier primary studies and frequency distribution of (non-)errors found in meta-analysis 1.*

	no error	error	
**non-outlier**	5	5	50%
**outlier**	7	3	50%
Total	12	8	20 (100%)

of the sample. Note that errors in Table 4 are classified as either a differently calculated effect, an effect that did not contain enough statistical information to reproduce, or an ambigious effect. To correct for the oversampling of outliers, we first calculated correction weights using type of effect size (outlier or non-outlier) as the auxiliary variable. By multiplying our sample frequencies with this weight, the proportion of (non-)outlier primary study effect sizes in the sample becomes the same as the proportion of (non-)outlier primary study effect sizes in the population. We first calculate g_h:

$$g_h = \frac{\frac{N_h}{N}}{\frac{n_h}{n}}, \text{ Bethlehem, 2011, f.8.6}$$

where we have two strata (h_1 and h_2, corresponding to respectively non-outlier and outlier primary study effect sizes), uppercase Ns refer to population sizes (either per stratum or in total), and lowercase ns to sample sizes. The correction weights for meta-analysis 1 are:

$$g_1 = \frac{\frac{N_1}{N}}{\frac{n_1}{n}} = \frac{\frac{37}{57}}{\frac{10}{20}} = 1.2982456$$

$$g_2 = \frac{\frac{N_2}{N}}{\frac{n_2}{n}} = \frac{\frac{20}{57}}{\frac{10}{20}} = 0.7017544$$

Since we do not have any information on the number of errors in the population, we assume the same weights for non-errors and errors within each of the strata. As such, we multiply the first row of Table 4 with g_1, and the second row of Table 4 with g_2, of which the results are presented in Table 5.

As Table 5 shows, the sample distribution of meta-analysis 1 is now proportional to the population distribution of meta-analysis 1. We used the estimates from Table 5 to calculate the (conditional) probabilities of finding an error (i.e., either a different, incomplete, or ambiguous effect) in a primary study in meta-

Table 5.*Sample distribution of (non-)outlier primary studies and frequency distribution of (non-)errors found in meta-analysis 1, weighted frequency count.*

	no error	error	
**non-outlier**	6.49	6.49	65%
**outlier**	4.91	2.11	35%
Total	11.40	8.60	20 (100%)

analysis 1, given that you either have a primary study effect size that is classified as a non-outlier or outlier.

$$P_{(error)} = \frac{8.60}{20} = 0.43$$

$$P_{(error|non-outlier)} = \frac{P_{(non-outlier\ and\ err)}}{P_{(non-outlier)}} = \frac{\frac{6.49}{20}}{\frac{(6.49+6.49)}{20}} = 0.50$$

$$P_{(error|outlier)} = \frac{P_{(outlier\ and\ err)}}{P_{(outlier)}} = \frac{\frac{2.11}{20}}{\frac{(4.91+2.11)}{20}} = 0.30$$

For all 33 meta-analyses we calculated the abovementioned three (conditional) probabilities. In order to obtain the chance of any given effect size in a meta-analysis being irreproducible, we weighed the probability estimate (i.e., *P(error)* with the number of total primary study effect sizes in that specific meta-analysis. For meta-analysis 1, this meant multiplying the probability estimate above with 57 (i.e., 0.43 × 57). After summing the weighted probability estimates of all 33 meta-analyses together and dividing by the total number of primary study effect sizes (1978), we estimated that across all meta-analyses, the chance of any randomly chosen primary study effect size being erroneous is 0.37.

References

Bethlehem, J. (2011). *Handbook of Nonresponse in Household Surveys.* Wiley-Blackwell.

Hunter, J. E., & Schmidt, F. L. (2004). *Methods of Meta-Analysis: Correcting Error and Bias in Research Findings.* New York City, NY: SAGE Publications, Inc.
